# Impaired Molecular Mechanisms Contributing to Chronic Pain in Patients with Hidradenitis Suppurativa: Exploring Potential Biomarkers and Therapeutic Targets

**DOI:** 10.3390/ijms26031039

**Published:** 2025-01-25

**Authors:** Uppala Radhakrishna, Murali R. Kuracha, Iltefat Hamzavi, Nazia Saiyed, Jignesh Prajapati, Rakesh M. Rawal, Lavanya V. Uppala, Giovanni Damiani, Uppala Ratnamala, Swapan K. Nath

**Affiliations:** 1 Department of Anesthesiology and Perioperative Medicine, University of Pittsburgh, Pittsburgh, PA 15261, USA; 2 Department of Internal Medicine, University of Nebraska Medicine, Omaha, NE 68198, USA; muralibabu@gmail.com; 3 Department of Dermatology, Henry Ford Hospital, Detroit, MI 48202, USA; ihamzavi@hamzavi.com; 4 Department of Obstetrics and Gynecology, Corewell Health William Beaumont University Hospital, Royal Oak, MI 48076, USA; nazia.saiyed110@gmail.com; 5 Department of Biochemistry & Forensic Sciences, Gujarat University, Ahmedabad 380009, India; jigneshprajapati7297@gmail.com; 6 Department of Botany, Bioinformatics and Climate Change Impacts Management, School of Science, Gujarat University, Ahmedabad 380006, India; rakesh.rawal@gbu.edu.in; 7 Peter Kiewit Institute, College of Information Science & Technology, The University of Nebraska at Omaha, Omaha, NE 68182, USA; uppala821@gmail.com; 8 Department of Biomedical, Surgical and Dental Sciences, University of Milan, 20122 Milan, Italy; dr.giovanni.damiani@gmail.com; 9 Italian Center of Precision Medicine and Chronic Inflammation, University of Milan, 20122 Milan, Italy; 10 Department of Life Sciences, School of Sciences, Gujarat University, Ahmedabad 380009, India; ratnapavuluri21@gmail.com; 11 Arthritis and Clinical Immunology Program, Oklahoma Medical Research Foundation, Oklahoma City, OK 73104, USA; swapan-nath@omrf.org

**Keywords:** epigenetics, hidradenitis suppurativa, biomarkers, chronic pain, bioinformatics

## Abstract

Hidradenitis suppurativa (HS) is a chronic skin condition that primarily affects areas with dense hair follicles and apocrine sweat glands, such as the underarms, groin, buttocks, and lower breasts. Intense pain and discomfort in HS have been commonly noted, primarily due to the lesions’ effects on nearby tissues. Pain is a factor that can influence DNA methylation patterns, though its exact role in HS is not fully understood. We aim to identify molecular markers of chronic pain in HS patients. We performed DNA methylome of peripheral blood DNA derived from a group of 24 patients with HS and 24 healthy controls, using Illumina methylation array chips. We identified 253 significantly differentially methylated CpG sites across 253 distinct genes regulating pain sensitization in HS, including 224 hypomethylated and 29 hypermethylated sites. Several genes with pleiotropic roles include transporters (*ABCC2*, *SLC39A8*, *SLC39A9*), wound healing (*MIR132*, *FGF2*, *PDGFC*), ion channel regulators (*CACNA1C*, *SCN1A*), oxidative stress mediators (*SCN8A*, *DRD2*, *DNMT1*), cytochromes (*CYP19A*, *CYP1A2*), cytokines (*TGFB1*, *IL4*), telomere regulators (*CSNK1D*, *SMAD3*, *MTA1*), circadian rhythm (*IL1R2*, *ABCG1*, *RORA*), ultradian rhythms (*PHACTR1*, *TSC2*, *ULK1*), hormonal regulation (*PPARA*, *NR3C1*, *ESR2*), and the serotonin system (*HTR1D*, *HTR1E*, *HTR3C*, *HTR4*, *TPH2*). They also play roles in glucose metabolism (*POMC*, *IRS1*, *GNAS*) and obesity (*DRD2*, *FAAH*, *MMP2*). Gene ontology and pathway enrichment analysis identified 43 pathways, including calcium signaling, cocaine addiction, and nicotine addiction. This study identified multiple differentially methylated genes involved in chronic pain in HS, which may serve as biomarkers and therapeutic targets. Understanding their epigenetic regulation is crucial for personalized pain management and could enhance the identification of high-risk patients, leading to better preventative therapies and improved maternal and neonatal outcomes.

## 1. Introduction

Hidradenitis suppurativa (HS) is a chronic skin disease marked by recurrent painful nodules, abscesses, and sinus tracts in apocrine gland-rich areas, such as the armpits, groin, and buttocks, often accompanied by severe comorbidities [1]. HS is a multifactorial condition with an unclear exact cause, but it is widely accepted to result from a combination of genetic and epigenetic factors, environmental and lifestyle triggers, chronic inflammation, and immune system dysfunction, which collectively underlie its pathogenesis [2,3,4,5,6,7]. HS often goes undiagnosed [8], due to the absence of biological, pathological, or specific diagnostic tests [9]. Pain is a central aspect of living with HS, with intense discomfort commonly caused by the lesions’ effects on surrounding tissues [10]. Abscesses, inflammation, and scarring contribute to varying levels of pain, from mild to severe, depending on the disease’s stage and severity [11]. These symptoms profoundly impact the quality of life, leading to discomfort, social isolation, and stigmatization [12].

Pain management is crucial in HS, as both acute and chronic pain have a significant impact on the well-being of HS patients [13]. Psychological factors and lifestyle-related conditions, including stress, anxiety, depression, poor sleep, obesity, and diabetes, are common comorbidities of HS and significantly influence pain perception [14,15,16,17]. Acute pain occurs during flare-ups triggered by abscesses, inflammation, infection, or post-surgical trauma and is often accompanied by anxiety or emotional distress, which can usually be diagnosed and treated [18]. Chronic pain, which persists even between flare-ups, is often caused by scar tissue formation, nerve involvement, and the development of sinus tracts under the skin [13,19]. This persistent pain is exacerbated by factors like malodorous discharge and scarring and can lead to depression, anxiety, fatigue, irritability, muscle weakness, sleep disruptions, and social isolation [10,20]. Acute pain, however, can become chronic, and both acute and chronic pain significantly impact the quality of life for individuals with HS [13].

DNA methylation, a fundamental epigenetic mechanism, is pivotal in gene regulation and has been linked to various conditions that cause pain, including chronic pain, post-surgical complications, obesity, cancer, fibromyalgia, neuropathic pain disorders, arthritis, migraines, and endometriosis [21]. Although HS-related pain has been studied extensively, including in large population-based studies [13,22,23,24], the mechanisms of this condition are complex and multifactorial, involving interactions among genetic, environmental, and epigenetic factors [25]. Earlier studies have shown that DNA methylation in genes related to ion channels, transporters, cytochromes, cytokines, non-coding RNAs, circadian rhythms, telomeres, glucose metabolism, and neural pathways plays a significant role in pain perception across various diseases including HS [26,27,28,29,30,31,32,33,34]. Furthermore, several pain-associated genes, including *OPRM1*, *ADRB2*, *CACNA2D3*, *GNA12*, *LPL*, *NAXD*, and *ASPHD1*, exhibit methylation variations [35]. Despite these findings, the precise mechanism of pain perception remains unclear, with HS-related pain yet to be evaluated in the context of genome-wide DNA methylation changes.

This study examines the link between DNA methylation and chronic pain in HS patients to identify epigenetic biomarkers of pain severity. By analyzing differentially methylated CpG sites in pain-related genes, we reveal the pathways involved in pain sensitization, potentially guiding personalized treatments and new pain management strategies for HS.

## 2. Results

We identified 253 significantly differentially methylated genes in HS patients, which are implicated in pain mechanisms, by leveraging a pain-associated published database. These genes, linked to pain sensitization, were analyzed for their biological roles using public datasets and compared with genome-wide data (Supplementary Table S3). Table 1 highlights 224 hypomethylated and 29 hypermethylated CpG sites (Also provided Supplementary Table S1) tab, including genes involved in transport (*ABCC2*, *SLC39A8*, *SLC39A9*); apocrine gland genes (*DOCK4*, *TUG1*); dopamine-related genes (*DDC*, *DRD2*, and *SLC6A3*); serotonin-associated genes (*HTR1D*, *HTR1E*, *HTR3C*, *HTR4*, *TPH2*); endorphin-associated genes (*BDNF MORC2 OPRM1 POMC*); oxytosin-related (*OXTR CD38*); risk of suicide (*DRD2*, *SLC6A3*, *BDNF*, *OPRM1*); wound healing (*MIR132*, *FGF2*, *PDGFC*), autophagy genes (*ZCCHC14*, *ULK1*, *MAP1LC3B*, *ESR2*); ion channel regulators (*CACNA1C*, *SCN1A*); oxidative stress mediators (*SCN8A*, *DNMT1*, *NR3C1*, *NOS3*), cytochromes (*CYP19A*, *CYP1A2*), cytokines (*TGFB1*, *IL4*), telomere regulators (*DNMT1*, *AHR*), circadian rhythm (*IL1R2*, *ABCG1*, *RORA GNA11*), ultradian rhythms (*PHACTR1*, *TSC2*, *ULK1*, *CLCN6*); hypoxia related (*MMP3*, *ACE*, *CX3CR1*, *NOTCH4*); hormonal regulation (*PPARA*, *NR3C1*, *ESR2*), glucose metabolism (*POMC*, *NR3C1*, *IRS1*, *GNAS*), and obesity (*DRD2*, *FAAH*, *MMP2*, *OPRM1*).

### 2.1. Heatmaps and Principal Component Analysis

**Figure 1:**
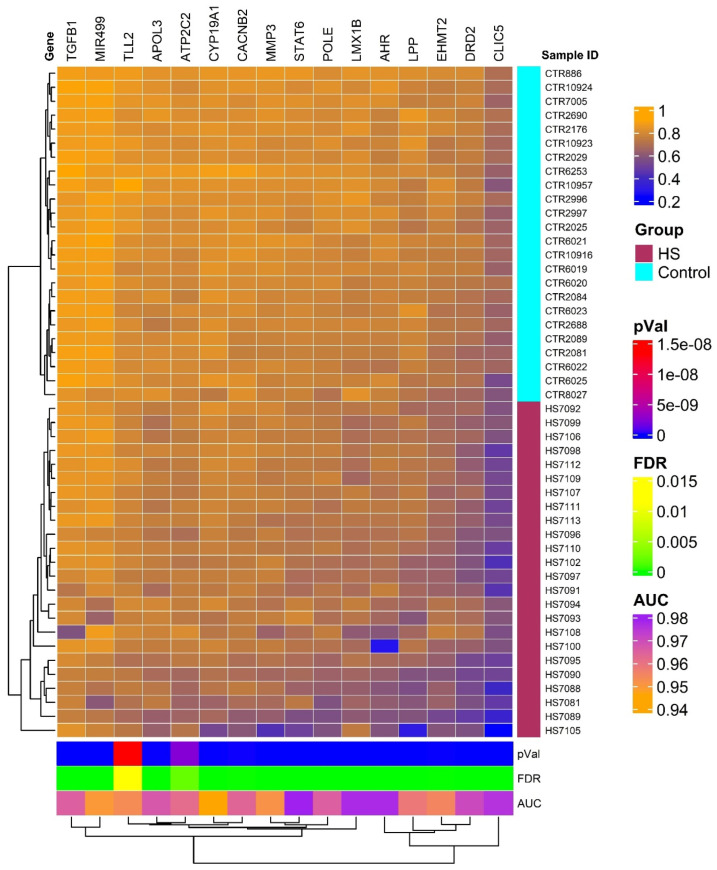
Hierarchical clustering and heatmap of methylation data of 16 distinct genes with significant methylation differences between HS patients and controls based on AUC and FDR filters. The heatmap colors indicate levels of methylation as detailed in the color legend, with patients exhibiting the HS phenotype shown in maroon and normal controls in cyan. This clear segregation enhances our understanding of the potential biological mechanisms underlying HS-related pain and may facilitate the development of targeted therapies.

The heatmap generated from our analysis delineates two distinct clusters of genes with differential methylation profiles, one representing individuals with HS and another healthy controls. This visualization is depicted in Figure 1 and underscores the substantial methylation differences between the two groups, reflecting the pathophysiological alterations in HS patients compared to unaffected individuals. The genes selected for this heatmap all showed high discriminative power, with AUC values > 0.9 and FDR *p*-values ≤ 0.05, confirming their relevance in the methylation patterns associated with HS. The hierarchical clustering used in this analysis further supports the robustness of these methylation markers in distinguishing between affected and non-affected individuals.

### 2.2. Principal Component Analysis

**Figure 2:**
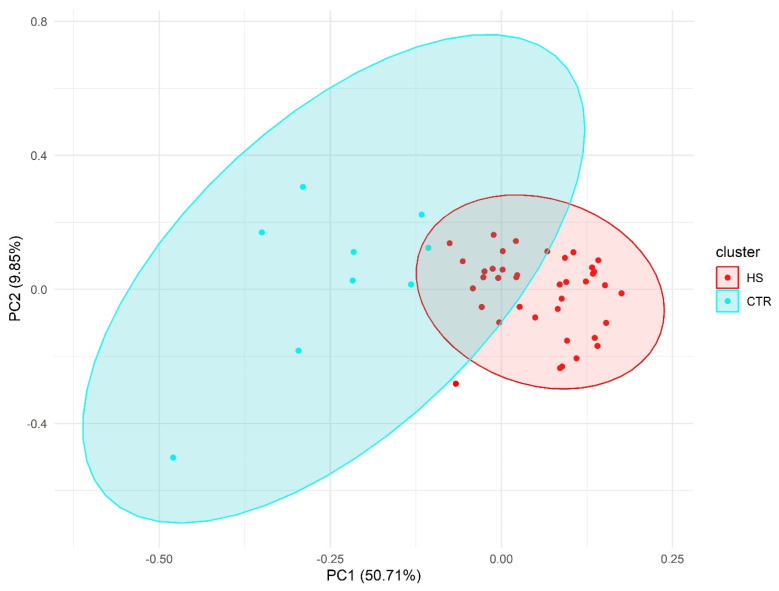
Principal component analysis (PCA) using pain-associated genetic markers.

PCA results showed a clear separation between HS individuals and the control group (Figure 2).

### 2.3. Network Analysis

**Figure 3:**
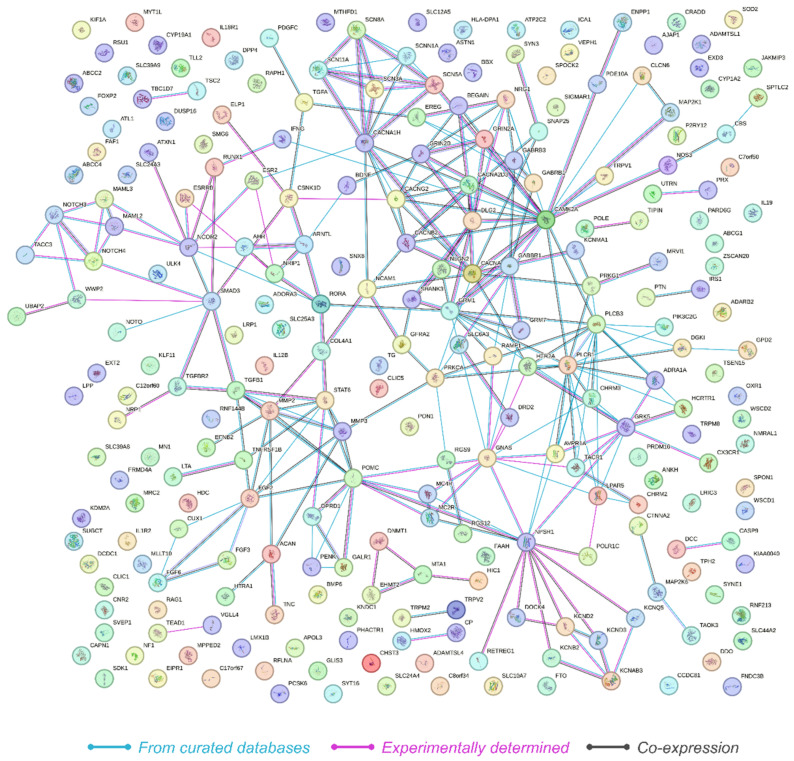
Illustration of the protein–protein interaction network derived from STRING analysis, showing 249 interconnected genes with significant biological coherence, as indicated by a PPI enrichment *p*-value of <1.0 × 10^−16^. Nodes represent genes, which are color-coded to denote the source of interaction data, with lines reflecting connectivity.

The protein–protein interaction (PPI) network constructed from the 249 genes revealed significant interaction dynamics as visualized in the PPI network (Figure 3). The network consisted of 249 nodes and 268 edges, with an average node degree of 2.15, indicating that on average, each protein interacts with approximately two other proteins. The average local clustering coefficient was calculated to be 0.321, suggesting a moderate level of clustering within the network. Importantly, the analysis revealed that the expected number of edges in a random network of similar size would be 110. The observed network had 268 edges, with a PPI enrichment *p*-value below 1.0 × 10^−16^. This highly significant enrichment indicates that the network has substantially more interactions than expected by chance, suggesting that the proteins are more interconnected and potentially involved in shared biological processes related to HS.

### 2.4. Gene Ontology (GO) and KEGG Pathway Analysis

**Figure 4:**
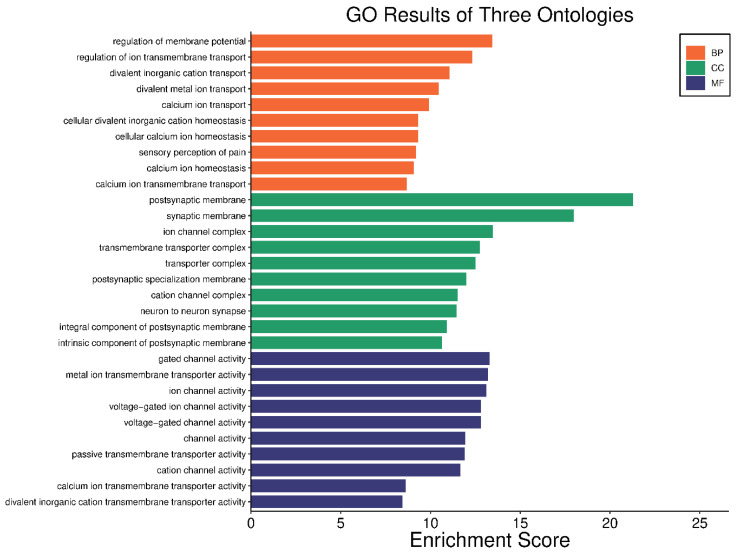
Visual representation of the top ten GO terms; biological processes, cellular components, and molecular functions. Each bar represents the enrichment score of the terms, illustrating the relative significance and impact of each term.

In our comprehensive gene ontology (GO) analysis, we identified significant enrichments across 688 biological processes (BP) terms, 64 cellular components (CC) terms, and 70 molecular functions (MF) terms, all with adjusted *p*-values < 0.05. For clarity and focus, the top ten teams from each category are visually represented in Figure 4. In the BP category, terms such as “regulation of membrane potential” and “regulation of ion transmembrane transport” were prominent, highlighting the importance of ion transport and membrane potential regulation in the pathophysiology of HS. Similarly, CC terms like “postsynaptic membrane” and “synaptic membrane” suggest alterations in synaptic structures that could play a critical role in the disease. In the MF category, terms such as “ion channel activity” and “voltage-gated ion channel activity” point to disruptions in ion channel functions, which are crucial for proper cell signaling and function. The complete list of enriched GO terms is available in Supplementary Tables S4–S6, providing a detailed breakdown of these categories.

### 2.5. KEGG Pathway Analysis

**Figure 5:**
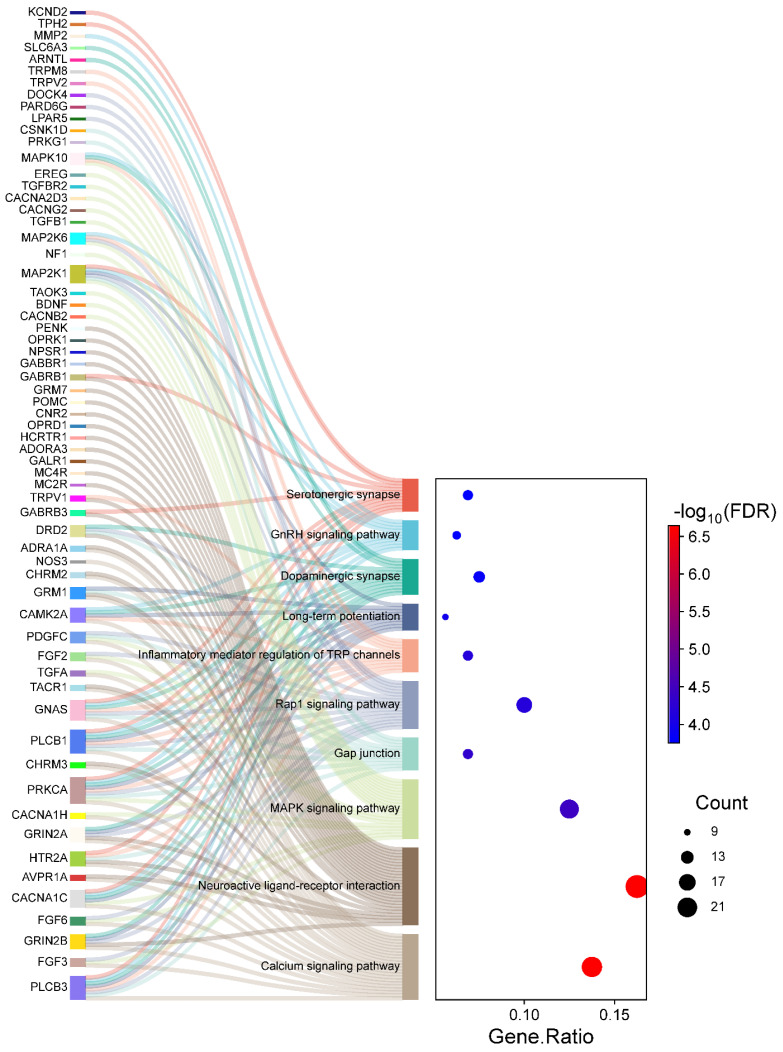
Sankey diagram and dot plot depicting the mapping of genes to significantly enriched KEGG pathways in HS. The left section (Sankey diagram) illustrates the flow from individual genes to their respective pathways. The right section (dot plot) quantifies pathway enrichment, where dot sizes correspond to the number of genes associated with each pathway, and the dot colors represent the statistical significance (−log10 FDR).

The KEGG pathway analysis identified 76 significantly enriched pathways, again with adjusted *p*-values < 0.05. For clarity and focus, the top ten pathways are visually represented in Figure 5. The top significantly enriched pathways identified in the KEGG analysis include the *Calcium signaling pathway*, *Glutamatergic synapse*, *Long-term potentiation*, and *Circadian entrainment*, all of which are integral to neuronal communication and inflammatory responses in HS. Notably, pathways related to neurotransmitter signaling and ion channel regulation, such as *Calcium signaling* and *Glutamatergic synapse*, underscore the role of disrupted neuronal processes in chronic pain mechanisms. As depicted in Figure 5, the left section (Sankey plot) visualizes the genes distributed across these enriched pathways, while the right section (dot plot) quantifies gene involvement. Specifically, the gene ratio measures the proportion of genes associated with each pathway relative to the total input genes, allowing for direct comparison of pathway involvement. Larger dots indicate higher gene counts within a pathway, and dot colors represent the statistical significance of the enrichment (FDR). Complete details of all enriched pathways are provided in Supplementary Table S7.

We observed that pathways like *Calcium signaling* and *Glutamatergic synapse* exhibit high gene counts and strong enrichment significance, indicating their prominent role in pain sensitization and inflammatory dynamics in HS patients. Similarly, pathways like *Long-term potentiation* and *Circadian entrainment* suggest broader implications in neuronal plasticity and circadian rhythm disruptions, which may exacerbate pain perception and other neurological symptoms. These findings highlight the functional relevance of these pathways and the identified genes, offering insights into their potential as therapeutic targets for managing chronic pain in HS.

## 3. Discussion

**Figure 6:**
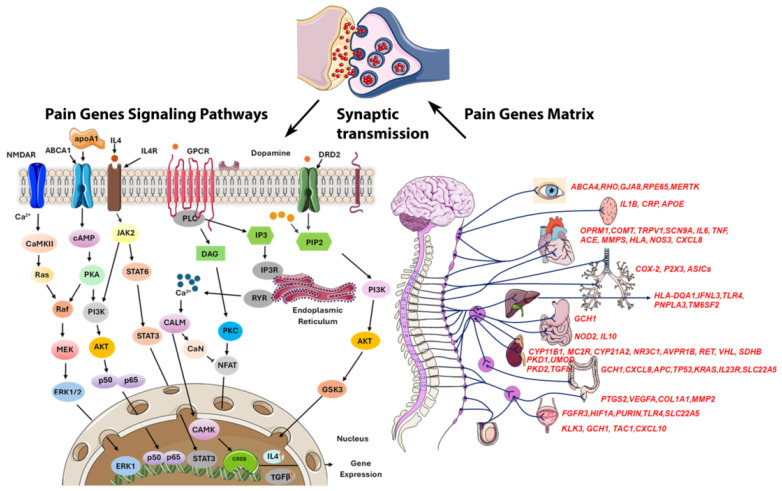
Schematic representation of pain genes and their regulatory pathways showing that most pain transmission is mediated by noxious stimuli via synaptic transmission in the synaptic cleft. Several pain-related genes are commonly expressed across all organs, while different organs utilize specific genes to modulate pain perception through distinct pathways. In the brain, genes like *TRPV1* and *SCN9A* are critical for detecting and modulating pain signals, while COMT influences pain sensitivity through neurotransmitter regulation. Inflammatory pain responses are prominent in all organs, with genes such as *IL6*, *TNF*, *IL1B*, *IL10*, and *IFNL3* playing key roles. Overall, these genes modulate pain through inflammatory responses, nerve sensitivity, and tissue damage across various organs.

Managing pain in HS is challenging due to its chronic nature, frequent flare-ups, and complex pain mechanisms like inflammation, infection, and mechanical stress, complicating personalized treatment [13]. Epigenetics are crucial in pain perception, with future research aiming at gene therapies due to the link between DNA methylation and chronic pain [36]. We identified 253 differentially methylated pain-related genes, many of which exhibit pleiotropy, contributing to a wide range of biological functions, including circadian rhythm regulation, inflammation, telomere maintenance, glucose metabolism, autophagy, cytochrome activity, neurotransmission, oxidative stress, and obesity (Figure 6). Among these, 224 CpGs were hypomethylated, and 29 CpGs were hypermethylated. Notably, hypomethylation appears to exacerbate pain to a greater extent than hypermethylation, though the effects are highly context-dependent, varying by individual and condition [37].

**Autophagy and Chronic Pain:** Recent evidence links dysregulated autophagy to chronic inflammation and pain [38]. We identified five key autophagy genes associated with pain: *BDNF*, *ESR2*, *LMX1B*, *PRKCA*, and *ULK4*. *BDNF*, which plays a role in brain signaling and synaptic plasticity, is proposed as a marker of nociception in chronic pain, particularly in the hippocampus, prefrontal cortex, and mesocorticolimbic system, potentially worsening chronic pain in HS by amplifying nociceptive signaling [39]. Estrogen receptor beta (*ESR2*) influences inflammation and pain perception, with signaling changes potentially increasing pain sensitivity in severe HS, especially in women with estrogen imbalances, highlighting the role of hormonal regulation in managing HS pain [40]. *LMX1B* influences skin development and inflammation, and its dysregulation could impact pain by affecting skin barrier integrity [41]. The *PRKCA* gene, encoding protein kinase C alpha (PKCα), plays a key role in signal transduction, inflammation, and pain modulation. It regulates pain signals, especially in inflammatory and neuropathic pain, by modulating ion channels like *TRPV1* and voltage-gated calcium channels, both crucial for pain transmission [42]. *ULK4* is crucial for the initiation and regulation of autophagy; disruptions in these kinases can lead to altered autophagic responses, exacerbating inflammation and pain [43].

**Telomeres and Chronic Pain:** Emerging studies suggest that telomere-related genes may impact pain perception and chronic pain severity [44]. We identified 31 dysregulated genes linked to telomere maintenance [44]. *DNMT1*, primarily known for its role in DNA methylation, shows emerging evidence linking it to pain modulation through epigenetic mechanisms [45]. Vitamin B12 deficiency can alter DNA methylation, potentially leading to *DNMT1* dysregulation [46]. Our genome-wide study identified four dysregulated genes: *CUBN* (vitamin B12 absorption), *AMN* (works with cubilin for absorption), *TCN1* (binds and protects vitamin B12), and *SLC12A5* (cobalt carrier crucial for vitamin B12 production and various biological functions). Vitamin B12 may be a complementary treatment for pain in HS. [47]. *CSNK1D* is associated with pain sensitivity, influencing cellular processes through protein phosphorylation [48]. *SMAD3*, part of the *TGF-β* pathway, influences inflammation and tissue repair, impacting pain conditions [49]. *DLG2*, associated with synaptic function, plays a role in chronic pain by regulating neuronal pain signaling [50]. Other telomere-associated genes involved in pain regulation, such as *CAMK2A*, *ESR2*, *AHR*, and *NOS3*, are detailed elsewhere.

**Circadian Rhythm and Chronic Pain:** The circadian rhythm, a natural internal process regulating the sleeping–waking cycle and other physiological functions, significantly impacts pain perception, with sensitivity typically peaking in the late afternoon and being lowest in the early morning [51]. Additionally, ultradian rhythms, which occur more frequently than once per day, also influence pain sensitivity. Circadian rhythm-related genes play a key role in regulating processes such as sleep, hormone release, and inflammation. Disruption of these genes can alter pain perception, increasing sensitivity and exacerbating chronic pain by disturbing the body’s natural rhythms and affecting pain pathways and immune responses. We identified 22 dysregulated genes associated with circadian rhythms and 9 genes implicated in ultradian rhythms linked to pain regulation [52]. Disruptions, like shift work, contribute to chronic pain, which is worsened by poor sleep. Sleep disturbances and psychological stress, including depression and anxiety, can worsen pain and promote a sedentary lifestyle and overeating. This rhythm affects pain-modulating substances like melatonin. Chronic pain disrupts sleep, creating a cycle where pain and sleep issues reinforce each other, with individual variations influencing susceptibility and the effectiveness of relief.

**Obesity contributes to chronic pain:** Obesity is linked to chronic inflammation, which exacerbates pain and conditions like rheumatoid arthritis and fibromyalgia [52]. It also raises the risk of metabolic syndrome, cardiovascular issues, nerve damage, and neuropathic pain [53]. Obese individuals often require higher pain medication doses, complicating management. Reduced activity weakens muscles and increases the risk of chronic conditions, worsening pain. We identified 31 genes linked to both obesity and pain, including *MC4R* (appetite regulation and obesity), *CYP19A1* (estrogen synthesis affecting body fat distribution), *IRS1* (insulin signaling linked to obesity), *FAAH* (endocannabinoid system in appetite regulation), and *DRD2* (dopamine in reward and pleasure affecting food consumption) [54], which play key roles in pain, obesity, and appetite regulation.

**Glucose Metabolism and Chronic Pain:** Glucose metabolism, which is vital for energy production, intricately influences pain perception through complex mechanisms [55]. We identified 19 genes related to glucose metabolism that may influence pain perception, including *AHR*, *CACNA1C*, *CACNA1H*, *CP*, *CYP19A1*, *ENPP1*, *EXT2*, *GLIS3*, *GNAS*, *GPD2*, *IRS1*, *KLF11*, *MC2R*, *MC4R*, *MMP2*, *NOTCH3*, *POMC*, *STAT6*, and *TGFA*. The interconnectedness of these genes with inflammatory, hormonal, and neural processes suggests a complex interplay between glucose metabolism and pain perception, emphasizing the need for further research [56]. Genes like *CACNA1C* (calcium regulation) and *AHR* (inflammation) may influence pain pathways, while *NOTCH3* is involved in immune responses and stress regulation, and anxiety may contribute to pain modulation.

**Chronic Pain Can Elevate Suicide Risk:** Persistent pain increases suicide risk by causing distress, lowering quality of life, and triggering depression and anxiety. We identified 57 dysregulated pain-related genes linked to suicide, affecting neurotransmission, inflammation, sensory processing, stress response, and hormonal regulation. *BDNF*, which is crucial for neuronal survival and plasticity, is linked to mood disorders and suicide risk, while *DRD2*, involved in dopamine signaling, affects mood regulation and susceptibility to depression and suicidal tendencies [57]. Glutamate receptors, including *GRIN2A* and *GRIN2B*, are vital for glutamatergic neurotransmission, impacting mood and being associated with suicidal behavior [58]. Serotonin-related genes, including the serotonin receptor gene *HTR2A*, play a crucial role in neuropsychiatric processes by modulating serotonin signaling in the brain. *TPH2* is vital for serotonin synthesis, ensuring adequate serotonin levels, while the serotonin transporter gene *SLC6A4* regulates serotonin reuptake and mood stability. Disruptions in these genes are associated with mood disorders and an increased risk of suicide.

The *FAAH*, part of the endocannabinoid system, also contributes to mood regulation and susceptibility to mood disorders and suicidal behavior [59]. The *POMC* gene, which regulates stress and mood, is linked to depression and suicidal tendencies when dysregulated [60]. Genetic variations in *NOS3*, affecting nitric oxide production, and *TGFB1*, involved in inflammation and tissue repair, further illustrate the complex interactions between genetic predispositions and mental health outcomes, both potentially contributing to increased suicide risk if dysregulated [61,62].

**Calcium and Chronic Pain:** Calcium ions are crucial for pain management, influencing sensitivity, signal transmission, and keratinocyte function; their dysregulation can heighten pain and delay healing. [63]. We identified 23 dysregulated calcium-associated genes involved in pain in the HS cohort. The *CACNA1C, CACNA2D3, CACNA1H*, and *CACNG2* genes encode various components and regulatory subunits of calcium channels vital for calcium signaling, affecting neurotransmitter release and excitability. *CACNA1C* and *CACNA1H* encode L-type and T-type calcium channels, respectively, which are key in pain signal transmission [64]. *CACNA2D3* and *CACNG2* also affect calcium signaling, and their dysfunction can exacerbate pain and emotional distress [65,66]. *CAMK2A*, involved in pain pathway sensitization, may perpetuate pain hypersensitivity due to persistent inflammation in HS. *CHRM2* modulates pain via acetylcholine signaling, and disruptions in cholinergic signaling in HS could worsen pain perception. Additionally, studies in animals have shown that *CHRM3* is essential for epidermal development, improving basal cell adhesion and inhibiting their proliferation [67]. NMDA receptor *GRIN2A* plays critical roles in synaptic plasticity and pain perception, with chronic pain in HS potentially associated with NMDA receptor-mediated central sensitization. *GRM1* modulates pain through G-protein signaling and calcium mobilization, and its persistent activation in HS could enhance pain states, *HCRTR1* influences pain sensitivity and stress-induced analgesia, and its dysregulation in HS could alter pain perception [68]. Serotonin receptor *HTR2A* modulates pain perception and analgesic efficacy [69].

**Potassium channels:** Pain perception in hidradenitis suppurativa may be significantly influenced by the dysregulation of potassium channels, including genes such as *KCNMA1*, *KCNQ5*, *KCNB2*, *KCND2*, *KCND3*, and *KCNAB3*, all of which were identified in this study. *KCNMA1* encodes a large-conductance calcium-activated potassium channel crucial for regulating neuronal excitability and pain signal transmission [70]. Dysregulation of *KCNMA1* can alter neuronal excitability, potentially worsening pain sensitivity in HS. *KCNB2*, *KCND2*, and *KCND3* are critical for voltage-gated potassium channels, which maintain normal neuronal function [71]. *KCNB2* regulates action potentials and neuronal excitability, impacting pain signal propagation. *KCND2* controls action potentials and firing rates, affecting neuronal responsiveness and pain perception. *KCND3* is vital for neuronal excitability and firing patterns, influencing pain processing. *KCNQ5*, also plays a role in voltage-gated potassium channels, further affecting neuronal activity and pain perception [72].

**Sodium channels:** We identified five dysregulated sodium channel genes: *SCN11A*, *SCN3A*, *SCN5A*, *SCN8A*, and *SCN11A*. They are mainly in peripheral neurons and nociceptors and transmit pain signals, with variations affecting pain thresholds [73]. *SCN11A* encodes a sodium channel essential for pain perception and neuronal excitability, with variants potentially disrupting pain signaling and exacerbating chronic pain [74]. *SCN3A*, upregulated in response to nerve injury, contributes to pain pathway development and hyperexcitability [75]. The *SCN5A* gene mainly regulates cardiac conduction but also affects sensory neurons and pain mechanisms, particularly in conditions with both cardiac and sensory symptoms [76]. *SCN8A* gene encodes the Naᵥ1.6 sodium channel, which is essential for action potential generation in neurons [77]. Variations in *SCN8A* can increase neuronal excitability, heightening pain sensitivity (hyperalgesia) or causing pain from non-painful stimuli (allodynia). The *SCN11A* gene encodes the Na_v_1.9 sodium channel, which is vital for pain perception. *SCN11A* variants are notably associated with familial episodic pain syndrome (FEPS), a severe pain disorder in early childhood.

**Transporters and chronic pain:** Transporters regulating ion and lipid balance are crucial for inflammation and pain. In HS, dysregulation of 12 key transporters, including *ABCC2*, *ABCG1*, and SLC genes, worsens chronic pain and inflammation. Genes such as *ABCC2* and *ABCC4* play a crucial role in the efflux of inflammatory mediators and drugs, and their dysregulation may lead to impaired drug metabolism and persistent inflammation, contributing to inadequate pain control in HS patients. Similarly, *ABCG1* is pivotal in regulating lipid homeostasis and macrophage function, with its disruption potentially exacerbating inflammation and chronic pain [78]. *SLC10A7* impacts inflammatory pathways and cellular homeostasis, while *SLC12A5*, also known as (*KCC2*), influences neuronal excitability, potentially leading to neuropathic pain due to the dysregulated inflammatory response in HS [79]. The *SLC24A3* and *SLC24A4* regulate intracellular sodium and calcium levels, with their dysregulation potentially disrupting ion balance and cellular signaling, contributing to inflammation and pain in HS. Mitochondrial function, as mediated by *SLC25A3*, is another critical factor, where impaired ATP production and increased oxidative stress may contribute to chronic pain and fatigue in HS [80]. Furthermore, *SLC39A8* and *SLC39A9* are key in zinc transport and immune regulation, with their dysregulation amplifying inflammatory responses and sustaining pain. *SLC44A2* and *SLC6A3* influence cholinergic and dopaminergic signaling, respectively, where alterations in these pathways may lead to heightened pain perception and chronic pain due to central sensitization in HS.

**Addiction Caused by Chronic Pain:** Opioids are commonly prescribed for post-surgical, cancer-related, and chronic pain management, but their use carries a substantial risk of addiction, particularly in those with chronic pain. We identified ten genes—*BDNF*, *DRD2*, *CNR2*, *FAAH, GABBR1*, *SLC6A3*, *GRM1*, *OPRD1*, and *CHRM2*—that may play crucial roles in neurotransmission and reward pathways, influencing susceptibility and response to substances. *BDNF* is essential for brain plasticity and the reward system, with certain variants linked to higher addiction risk [81]. *DRD2* plays a key role in dopamine signaling, which influences reward, motivation, and pain perception, with variants influencing addiction and drug response [82]. *CNR2*, part of the endocannabinoid system, affects reward pathways and addiction [83]. *FAAH* modulates endocannabinoid metabolism and reward systems [84]. *GABBR1* is involved in GABAergic neurotransmission, with variants potentially affecting addiction susceptibility [85]. *SLC6A3* (*DAT1*) controls dopamine reuptake, influencing dopamine levels, mood, and pain sensitivity, with dysregulation often seen in chronic pain conditions, impacting addiction risk [82]. *GRM1* and *OPRD1* contribute to addiction through their roles in neurotransmission and opioid signaling, while *CHRM2* influences cholinergic signaling, affecting reward and addiction pathways [86].

**Immunomodulators and Chronic Pain:** The immune system is crucial for maintaining homeostasis and responding to injury. Immunomodulators, including cytokines and chemokines, regulate the balance between pro-inflammatory and anti-inflammatory processes. Disruptions in these pathways can lead to chronic inflammation, a major factor in chronic pain in HS [52]. We identified 27 dysregulated genes in HS that act as immunomodulators, each playing distinct roles in regulating immune responses and pain perception. Notably, genes such as *PRKCA, POMC, BDNF*, and *DNMT1* are involved in signaling pathways that modulate pain sensitivity and inflammatory responses [42,45,87]. *SOD2*, *TGFB1*, *IFNG*, and *SMAD3* contribute to the management of oxidative stress and the regulation of both pro-inflammatory and anti-inflammatory cytokine production [52,88]. *CX3CR1* and *NCAM1* are associated with immune cell recruitment and neural activity, influencing inflammation and pain pathways [89,90]. Furthermore, *RUNX1*, *MMP2*, *AHR*, *STAT6*, *TGFA*, *LTA*, *TNFRSF1B*, *IL1R2*, and *NRG1* play critical roles in immune regulation, cell survival, and inflammation, all of which impact chronic pain conditions. The involvement of *EREG*, *FGF2*, *IL12B*, *IL19*, *IL18R1*, *DPP4*, *TNC*, and *TGFBR2* underscores the complex interplay between immune modulation and pain in HS, affecting various levels of gene expression and signaling cascades.

**Endocrine Functions and Pain:** Chronic pain in HS may be influenced by dysregulated hormonal and endocrine genes. We identified four genes involved in endocrine functions that could potentially play roles in pain mechanisms in HS. *POMC* produces peptides like ACTH and beta-endorphin, with beta-endorphins playing a key role in pain modulation [91]. Dysregulation of *POMC* can result in abnormal beta-endorphin levels, affecting pain sensitivity and inflammation in HS patients. *TG* is a glycoprotein essential for thyroid hormone synthesis. Although *TG* itself is not directly linked to pain, thyroid dysfunction, reflected by abnormal *TG* levels, can affect systemic inflammation and pain perception. Thyroid disorders linked to inflammatory skin conditions suggest that *TG* dysregulation may worsen pain in HS by affecting thyroid function and inflammation [92]. *MC2R*, a receptor for *ACTH*, regulates adrenal function and stress responses. *MC2R* signaling influences cortisol release, which in turn modulates inflammation and pain [93]. *CYP19A1 (Aromatase)* converts androgens to estrogens, which are known to modulate pain sensitivity [94]. Dysregulation of *CYP19A1* can lead to imbalances in estrogen levels, affecting pain perception and inflammatory responses.

**Metal-Linked Genes in Chronic Pain:** Dysregulations in metal-associated genes could contribute to the chronic pain experienced by HS patients. Several metal-associated genes were found to be dysregulated in the study, including six zinc-related *SLC39A8*, *SLC39A9*, *MIR132*, *MMP2*, *MMP3*, and *CBS.* Zinc deficiency can lead to liver and pancreatic dysfunction, kidney impairment, chronic inflammation, delayed wound healing, and a weakened immune response, increasing vulnerability to various diseases. Dysregulation of *SLC39A8* and *SLC39A9* disrupts zinc balance, impairing immunity and raising oxidative stress. *MIR132* heightens inflammation and pain sensitivity, while matrix metalloproteinases (MMPs), specifically *MMP-2* and *MMP-3*, significantly impact the pathogenesis of hidradenitis suppurativa (HS). These enzymes are involved in the breakdown of extracellular matrix (ECM) components, such as collagen, which is crucial for maintaining skin integrity. The overexpression of *MMP2* can lead to excessive ECM degradation, resulting in tissue destruction and the formation of sinus tracts. Elevated *MMP3* levels can further exacerbate inflammation by processing pro-inflammatory cytokines, such as *TNF-α* and *IL-1β*, leading to a chronic inflammatory state. *CBS* dysfunction worsens oxidative stress and impairs cellular repair, all contributing to persistent pain in HS. Iron is crucial in HS by regulating oxidative stress, immune function, and inflammation. An imbalance in iron can exacerbate tissue damage, impair healing, and worsen HS symptoms. Dysregulated *SOD2* increases oxidative stress and inflammation, *CACNG2* and *CACNA2D3* impair calcium channels and heighten pain sensitivity, BMP6 disruption hampers tissue repair and aggravates inflammation, and CP (ceruloplasmin) disruption worsens iron metabolism, further escalating oxidative stress and HS severity. As detailed elsewhere, manganese-related (*SLC39A8*), cobalt-linked (*SLC12A5*), and lithium-associated (*CACNG2*) were found to be dysregulated in the study. Manganese supports manganese superoxide dismutase (MnSOD), an enzyme that neutralizes oxidative stress. Since oxidative stress is central to chronic inflammation in HS, sufficient manganese levels are essential for managing oxidative damage in HS lesions. Cobalt is crucial for vitamin B12, which supports DNA synthesis and cellular metabolism. Imbalances in cobalt can disrupt these processes and potentially worsen inflammatory conditions like HS. Lithium affects mood, neuronal health, and cellular signaling, potentially influencing inflammatory and immune responses in HS, though its direct link to the condition is not well established.

**Limitations:** The study identifies potential pain markers for HS but has limitations, including the use of blood samples instead of lesion tissues. Blood samples were chosen for ease of access and minimal invasiveness [95], allowing longitudinal studies and capturing broad epigenetic changes linked to inflammation and immune responses. While lesion tissues might offer more localized data, blood profiles still reflect key molecular alterations associated with pain and disease severity. Future research should include tissue-specific analyses. Additionally, the study’s focus on Asian Indian HS patients limits geographic diversity, and broader populations should be included for generalizability. Although the identified differentially methylated CpG sites are promising, further validation is required to confirm their specificity for HS-related pain.

**Conclusions:** This study represents the first genome-wide analysis demonstrating that distinct DNA methylation patterns directly influence the pain modulatory system in HS. Our findings introduce a novel epigenetic mechanism, highlighting specific gene methylation signatures that are closely associated with pain perception and sensitivity in HS patients. These pain-related epigenetic modifications offer insights into the biological pathways underlying chronic pain in HS and hold promise as biomarkers. They could pave the way for targeted therapies, enabling personalized treatments that modulate pain pathways and improve outcomes. By linking epigenetic regulation to pain in HS, this research lays the groundwork for future studies to explore how these changes can be harnessed for clinical benefit, potentially leading to novel pain management strategies.

## 4. Materials and Methods

**Study design:** The Institutional Review Board of Beaumont Health System, located in Royal Oak, MI, USA, approved this research (HIC#: 2015-172). This study adhered to ethical standards by obtaining written informed consent from all participants and following the Helsinki Declaration principles. Rigorous matching criteria—such as age, gender, and body mass index (BMI)—were used to pair individuals with HS with healthy controls, ensuring the reliability and significance of the results.

### Hidradenitis Suppurativa Sample Selection

**Inclusion and exclusion criteria:** HS patients were clinically evaluated for symptoms and severity by three independent board-certified dermatologists (RR, DGS, TM) at VS Hospital in Ahmedabad, India, using a questionnaire [96], based on the European Hidradenitis Suppurativa Foundation (EHSF) guidelines [97]. The diagnostic process and sample assessment followed these guidelines, with disease severity evaluated using established scoring systems, including the Hurley score [98], the HS Severity Score System (IHS4) [5], and the Autoinflammatory Disease Damage Index (ADDI) [99]. These systems were used to evaluate the severity and impact of the condition on those affected.

**Inclusion criteria:** Adult patients (>20 years) included in the study met the following criteria: (a) diagnosed with HS for more than 5 years, (b) had at least moderate Hurley II severity, (c) scored more than 3 points on the International Hidradenitis Suppurativa Severity Score System (IHS4), (d) scored less than 3 points on the Autoinflammatory Disease Damage Index (ADDI), (e) newly diagnosed (<3 months), or (f) untreated for the past 6 months. This ensured a comprehensive understanding by including patients with a confirmed HS diagnosis for over 5 years, representing the chronic phase of the disease, to investigate long-term molecular adaptations, including epigenetic modifications and sustained gene expression changes driven by chronic inflammation and pain. Additionally, the inclusion of patients newly diagnosed within the last three months allowed us to assess early epigenetic alterations before long-term changes occurred. These inclusion criteria encompass both chronic and early-stage disease dynamics, offering valuable insights into the spectrum of molecular changes in HS.

**Exclusion criteria**: The exclusion criteria were as follows: (a) syndromic HS defined by Van der Zee and Jemec clinical phenotypes, (b) smoking, (c) fasting regimens or specific diets other than an omnivore, (d) alcohol abuse (Alcohol Use Disorders Identification Test (AUDIT) > 7 points), (e) drug addiction, (f) use of concurrent medications, including contraceptives, and TRG (taste receptor gene) inducers such as grapefruits which inhibit *CYP3A4* and alter drug metabolism and treatment outcomes in HS (and also affect inflammation, complicating the analysis of genetic and immune markers), (g) previous treatment for HS, (h) chronic inflammatory or infectious diseases, (i) history of cancer within the past 5 years, and (j) individuals unable to provide informed consent for any reason.

**DNA extraction, bisulfite conversion:** Blood samples were obtained from 24 individuals diagnosed with HS and 24 healthy controls. Genomic DNA was extracted using the Gentra Puregene^®^ Blood Kit (Qiagen, Germany). DNA methylation patterns were analyzed after sodium bisulfite conversion of the extracted DNA, following the manufacturer’s protocol with the EZ 96-DNA Methylation Kit (Zymo Research, Irvine, CA, USA).

**Illumina Infinium Methylation EPIC BeadChip:** Genome-wide DNA methylation profiling was performed using the Infinium MethylationEPIC BeadChip (450K) array (Illumina Inc., San Diego, CA, USA) with bisulfite-treated genomic DNA, following the manufacturer’s protocol, as previously described in detail in a prior publication [100,101].

**Statistical and bioinformatic analysis:** The data were analyzed using the GenomeStudio methylation analysis software (1.9.0) from Illumina. Methylation levels (β-values) for each CpG site were calculated. Before analysis, CpG probes with missing β-values were excluded. Differential methylation was evaluated by comparing β-values for each CpG locus between HS and controls. To minimize confounding, we excluded probes linked to sex chromosomes and those containing SNPs, particularly those with dbSNP entries within 10 bp of the CpG site; as binding sites can influence interactions, only CpG targets with allele frequencies ≤ 0.05 were included for further analysis.

The most discriminating CpG sites were selected based on a preset cutoff criterion: FDR *p* < 0.05. For genes with multiple CpG sites, the site with the highest AUC ROC (≥0.75) was selected alongside the lowest *p*-value. The *p*-value for methylation differences between case and control groups at each locus was calculated as previously described [100,101]. Raw and FDR *p*-values corrected for multiple testing (Benjamini–Hochberg test) were calculated. The AUC for combinations of loci was calculated using the ‘R’ program “ROCR” package (v3.5.0).

**Selection of Candidate Genes for Chronic Pain:** Pain-associated genes were identified by utilizing a genetic database that consolidates data from genome-wide association studies (GWASs), gene expression profiles, and curated research focused on pain-related phenotypes [102,103]. We focused on genes with strong GWAS evidence linking them to pain perception or chronic pain and those documented in pain pathways, particularly in chronic pain management. Detailed information on these genes, including functions, pathways, and relevant SNPs or mutations, was extracted and cross-referenced with other databases to ensure accuracy and relevance, especially in the context of chronic pain and genetic diseases. Moreover, to identify genes potentially related to pain, relevant review articles were reviewed [104,105,106,107,108]. These articles offered crucial insights by compiling and analyzing existing studies, particularly those focused on genes tied to pain perception, chronic pain, and opioid use. Additionally, we utilized the Human Pain Genetics Database (https://diatchenko.lab.mcgill.ca/hpgdb/ (accessed on 25 August 2024)), a hand-curated resource compiling genetic associations with human pain phenotypes. Genes that overlapped between this database and the published literature were included only once. The complete list of genes used in these analyses is provided in Supplementary Table S2 (n = 897).

**Heatmap:** The heatmap was generated using the ‘ComplexHeatmap’ package (version 1.6.0) within R (version 3.2.2), designed to display the methylation patterns of CpG sites strongly associated with pain in HS. For this analysis, only genes exhibiting an AUC > 0.9 and FDR values ≤ 0.05 were included, ensuring that the displayed methylation differences were both statistically significant and highly discriminative between HS patients and controls. To assess the relationships and similarities among samples, hierarchical cluster analysis was performed. This analysis utilized Ward’s method, which minimizes the sum of squares of any two (hypothetical) clusters that can be formed at each. This method is particularly effective in identifying distinct groups by examining the methylation profiles of the CpG sites [109,110].

**Principal component analysis:** Principal component analysis (PCA) is widely utilized as a dimensionality reduction technique to improve data visualization and facilitate feature extraction by simplifying complex datasets. In this study, PCA was employed to pinpoint the key features responsible for the greatest variance across groups, which is particularly useful in fields such as epigenetics. The R function ‘prcomp’ was applied to calculate the principal components (PCs), and PC1, PC2, and PC3 were selected for visualization. A PCA distribution plot was created using the ‘ggplot2’ package in R”.

**Protein–Protein Interaction Network:** The 253 significantly differentially methylated CpG sites associated with HS were used to match corresponding genes against the STRING database (version 12.0, https://string-db.org/, accessed on 29 August 2024) to identify known and predicted protein–protein interactions. [111]. The interaction network was constructed using the full STRING network, which includes both functional and physical protein associations. Edges in the network were defined based on evidence, with line color indicating the type of interaction evidence. Interactions were considered from curated databases, experimentally determined interactions, and gene co-expression data, while sources such as text mining, neighborhood, gene fusion, and co-occurrence were excluded. A medium confidence threshold of 0.400 was applied to ensure the reliability of the interactions included in the analysis. This process resulted in 249 genes with validated interactions, which were further explored to understand their interaction dynamics.

**Gene Ontology and KEGG Pathway Analysis:** Gene ontology (GO) and Kyoto Encyclopedia of Genes and Genomes (KEGG) pathway analyses were performed on differently methylated genes. Both the GO and KEGG pathway analyses were conducted using the ‘clusterProfiler’ and ‘Pathview’ packages in R, respectively [112,113]. These tools facilitate the statistical analysis and visualization of functional profiles of genes, which are essential for interpreting the biological themes among gene clusters. This approach improves our understanding of genomic data and reveals altered pathways in HS.

To visually represent the KEGG pathway analysis results, we utilized the SRplot web server (http://www.bioinformatics.com.cn/SRplot (accessed on 25 August 2024)) to generate a Sankey diagram and a dot plot [114]. The Sankey diagram illustrates the flow and connection between enriched KEGG pathways and associated genes, providing an intuitive depiction of pathway relationships. The dot plot highlights the statistical significance and gene ratios of the enriched pathways, offering a concise overview of key insights from the analysis.

## Figures and Tables

**Table 1:** Top 25 significantly differentially methylated CpG sites associated with pain in HS cases (based on FDR-adjusted *p*-values).

Target ID	Genes	Location	*p*-Val	FDR *p*-Val	% Methylation			AUC		CI
					Cases	Control	Difference	Lower	Upper	
cg27280396	RORA	15q22.2	3.05 × 10^−39^	2.64 × 10^−33^	27.29	15.67	11.61	0.89	0.80	0.99
cg09633973	HIC1	17p13.3	6.69 × 10^−38^	5.79 × 10^−32^	19.45	11.02	8.42	0.84	0.73	0.96
cg09820084	RNF144B	6p22.3	2.98 × 10^−37^	2.58 × 10^−31^	12.15	5.36	6.80	0.79	0.66	0.92
cg16553796	PARD6G	18q23	6.72 × 10^−29^	5.82 × 10^−23^	23.84	40.87	−17.03	0.82	0.70	0.94
cg03466780	EXD3	9q34.3	3.32 × 10^−28^	2.88 × 10^−22^	38.93	62.16	−23.23	0.77	0.63	0.90
cg14984943	CACNA2D3	3p21.1-p14.3	1.59 × 10^−24^	1.38 × 10^−18^	63.36	77.64	−14.28	0.92	0.84	1.00
cg01364755	ADARB2	10p15.3	3.05 × 10^−24^	2.64 × 10^−18^	91.33	97.30	−5.97	0.85	0.73	0.96
cg14311251	COL4A1	13q34	4.25 × 10^−22^	3.68 × 10^−16^	51.37	66.76	−15.40	0.92	0.84	1.00
cg22572258	KCND2	7q31.31	2.18 × 10^−21^	1.88 × 10^−15^	48.31	63.73	−15.42	0.92	0.84	1.00
cg03666597	CUX1	7q22.1	5.58 × 10^−21^	4.82 × 10^−15^	38.87	54.45	−15.58	0.88	0.77	0.98
cg07913781	NRP1	10p11.22	1.59 × 10^−20^	1.38 × 10^−14^	86.04	93.37	−7.33	0.79	0.66	0.92
cg17978764	CLIC5	6p21.1	1.87 × 10^−20^	1.62 × 10^−14^	50.32	65.24	−14.93	0.98	0.93	1.00
cg13050884	FNDC3B	3q26.31	2.55 × 10^−20^	2.20 × 10^−14^	59.93	73.61	−13.68	0.89	0.79	0.98
cg11615758	PHACTR1	6p24.1	3.30 × 10^−20^	2.86 × 10^−14^	59.06	72.84	−13.78	0.94	0.87	1.00
cg16201095	ABCC2	10q24.2	9.14 × 10^−19^	7.90 × 10^−14^	88.21	94.72	−6.51	0.89	0.79	0.98
cg21752295	BMP6	6p24.3	2.02 × 10^−19^	1.75 × 10^−13^	63.54	76.32	−12.79	0.91	0.83	1.00
cg09601770	DPP4	2q24.2	4.93 × 10^−19^	4.26 × 10^−13^	10.64	20.92	−10.28	0.78	0.65	0.91
cg05707781	LPP	3q27.3-q28	9.41 × 10^−19^	8.14 × 10^−13^	65.84	77.99	−12.15	0.96	0.90	1.00
cg22018565	ADORA3	1p13.2	1.75 × 10^−18^	1.51 × 10^−12^	70.46	81.58	−11.12	0.76	0.62	0.90
cg16873130	CHRM2	7q33	2.58 × 10^−18^	2.23 × 10^−12^	48.61	62.88	−14.27	0.91	0.82	1.00
cg18356276	AHR	7p21.1	2.59 × 10^−18^	2.24 × 10^−12^	66.70	78.55	−11.85	0.98	0.93	1.00
cg11995490	C7orf50	7p22.3	2.70 × 10^−17^	2.34 × 10^−11^	71.69	82.21	−10.52	0.78	0.65	0.91
cg07035454	OXR1	8q23.1	3.96 × 10^−17^	3.43 × 10^−11^	57.42	70.37	−12.95	0.88	0.77	0.98
cg21189939	SMG6	17p13.3	1.14 × 10^−16^	9.83 × 10^−11^	78.66	87.36	−8.70	0.77	0.64	0.91
cg21671386	DRD2	11q23.2	1.24 × 10^−16^	1.07 × 10^−10^	61.12	73.38	−12.26	0.97	0.92	1.00

## Data Availability

The published article and its Supplementary Materials contain all the data generated during this study.

## Supplementary Materials

The following supporting information can be downloaded at: https://www.mdpi.com/article/10.3390/ijms26031039/s1.
