# Supplementary material for: Impaired Molecular Mechanisms Contributing to Chronic Pain in Patients with Hidradenitis Suppurativa: Exploring Potential Biomarkers and Therapeutic Targets
Source: Int J Mol Sci. 2025 Jan 25;26(3):1039. doi: 10.3390/ijms26031039 (PMC11817842; doi:10.3390/ijms26031039)
Supplement: Supplementary file 1 [file ijms-26-01039-s001.zip › Supplementary Table S1.pdf]

Supplementary Table S1. Significantly Differentially Methylated CpG Sites (Ranks 26-253) Associated with Pain in HS Cases.

					% Methylation			AUC		
Target ID	Genes	location	p-Val	FDR p-Val	Cases	Control	Difference	lower	upper	CI
cg03454669	KLF11	2p25.1	4.01E-16	3.47E-10	89.12	94.80	-5.68	0.76	0.62	0.89
cg23593986	MIR499	20q11.22	4.47E-16	3.87E-10	79.89	88.14	-8.25	0.95	0.88	1.00
cg18617860	SNAP25	20p12.2	6.39E-16	5.53E-10	72.20	82.21	-10.01	0.83	0.71	0.95
cg14042137	LMX1B	9q33.3	1.37E-15	1.19E-09	69.21	79.73	-10.52	0.98	0.93	1.00
cg06620397	CAMK2A	5q32	1.92E-15	1.66E-09	86.58	92.88	-6.31	0.93	0.85	1.00
cg16925177	KCNB2	8q21.11	2.52E-15	2.18E-09	53.17	65.89	-12.71	0.88	0.78	0.98
cg26848300	TRPV1	17p13.2	6.60E-15	5.71E-09	70.53	80.58	-10.04	0.83	0.71	0.95
cg00042478	ANKH	5p15.2	1.64E-14	1.42E-08	84.24	91.00	-6.76	0.78	0.64	0.91
cg02152233	OPRD1	1p35.3	1.96E-14	1.70E-08	81.51	88.96	-7.45	0.82	0.70	0.94
cg21161187	SLC6A3	5p15.33	2.41E-14	2.08E-08	85.73	92.06	-6.33	0.75	0.61	0.89
cg24182581	DLG2	11q14.1	3.17E-14	2.75E-08	61.30	72.64	-11.34	0.93	0.85	1.00
cg03224459	SNX8	6q14.2	3.74E-14	3.23E-08	90.54	95.48	-4.94	0.75	0.61	0.89
cg07306676	POLE	12q24.33	4.70E-14	4.06E-08	69.65	79.57	-9.93	0.97	0.91	1.00
cg23797439	PLCB1	20p12.3	5.96E-14	5.16E-08	14.01	7.14	6.87	0.88	0.77	0.98
cg01516887	GRK5	10q26.11	6.52E-14	5.64E-08	69.24	79.19	-9.95	0.78	0.65	0.91
cg20067272	GABBR1	6p22.1	7.10E-14	6.14E-08	83.94	90.64	-6.70	0.90	0.81	0.99
cg10635895	CACNA1C	12p13.33	7.84E-14	6.78E-08	52.80	64.90	-12.10	0.79	0.66	0.92
cg07170252	ESRRB	14q24.3	9.49E-14	8.20E-08	67.42	77.64	-10.22	0.78	0.65	0.91
cg26639039	SLC12A5	20q13.12	1.05E-13	9.11E-08	18.33	10.63	7.70	0.88	0.78	0.98
cg06760904	MYT1L	2p25.3	1.23E-13	1.07E-07	76.99	85.29	-8.30	0.80	0.67	0.93
cg19995964	DCC	18q21.2	1.61E-13	1.39E-07	79.39	87.12	-7.72	0.88	0.77	0.98
cg21913897	AJAP1	1p36.32	1.63E-13	1.41E-07	89.35	94.52	-5.17	0.86	0.76	0.97
cg11728143	TBC1D7	6p24.1	1.89E-13	1.63E-07	56.49	68.09	-11.60	0.91	0.83	1.00
cg26514793	CHST3	10q22.1	2.36E-13	2.04E-07	57.41	68.87	-11.46	0.92	0.84	1.00
cg24603573	ABCC4	13q32.1	2.83E-13	2.45E-07	69.14	78.89	-9.75	0.81	0.69	0.93
cg09489285	TGFB1	19q13.2	2.88E-13	2.49E-07	80.90	88.20	-7.30	0.97	0.91	1.00

cg17439694	CP	3q24-q25.1	2.90E-13	2.51E-07	65.05	75.47	-10.42	0.86	0.75	0.97
cg07926491	STAT6	12q13.3	3.96E-13	3.42E-07	70.71	80.13	-9.41	0.98	0.94	1.00
cg27469283	NCOR2	12q24.31	3.97E-13	3.43E-07	77.29	85.37	-8.09	0.85	0.74	0.96
cg02083376	EXT2	11p11.2	3.97E-13	3.44E-07	67.99	77.88	-9.89	0.80	0.67	0.92
cg05786348	GPD2	2q24.1	4.99E-13	4.32E-07	56.94	68.30	-11.36	0.94	0.88	1.00
cg16606123	LRP1	12q13.3	6.75E-13	5.84E-07	88.22	93.59	-5.37	0.94	0.87	1.00
cg18113270	MMP3	11q22.2	1.25E-12	1.08E-06	71.86	80.89	-9.02	0.95	0.89	1.00
cg05477920	SLC10A7	4q31.22	1.45E-12	1.25E-06	60.47	71.23	-10.76	0.88	0.79	0.98
cg20495540	MPPED2	11p14.1	1.46E-12	1.26E-06	62.90	73.35	-10.45	0.86	0.76	0.97
cg11612098	SDK1	7p22.2	2.17E-12	1.88E-06	64.95	75.04	-10.08	0.86	0.76	0.97
cg02859866	FRMD4A	10p13	2.34E-12	2.02E-06	13.35	6.99	6.36	0.78	0.65	0.92
cg02594345	JAKMIP3	10q26.3	2.41E-12	2.08E-06	16.03	9.14	6.90	0.77	0.64	0.91
cg26242708	TG	8q24.22	2.46E-12	2.13E-06	68.08	77.66	-9.58	0.84	0.72	0.95
cg01969473	IL18R1	2q12.1	2.50E-12	2.16E-06	76.70	84.67	-7.97	0.80	0.68	0.93
cg12168509	HLA-DPA1	6p21.32	2.59E-12	2.24E-06	70.76	79.87	-9.11	0.89	0.80	0.99
cg03329408	DCDC1	11p13	2.97E-12	2.57E-06	87.44	92.88	-5.44	0.87	0.77	0.97
cg05138298	CACNA1H	16p13.3	3.20E-12	2.77E-06	85.52	91.43	-5.92	0.80	0.67	0.93
cg26519745	ABCG1	21q22.3	3.26E-12	2.82E-06	10.45	4.75	5.70	0.79	0.66	0.92
cg12674145	EFNB2	13q33.3	3.59E-12	3.11E-06	76.94	84.80	-7.87	0.85	0.74	0.96
cg05059922	CRADD	12q22	3.64E-12	3.14E-06	69.34	78.64	-9.30	0.77	0.63	0.90
cg14222434	PDE10A	6q27	3.81E-12	3.29E-06	85.13	91.12	-6.00	0.80	0.68	0.93
cg04871131	PON1	7q21.3	3.88E-12	3.35E-06	66.59	76.32	-9.73	0.81	0.69	0.93
cg03184141	ACAN	15q26.1	4.13E-12	3.57E-06	14.45	7.93	6.52	0.85	0.74	0.96
cg25920665	IL1R2	2q11.2	4.27E-12	3.69E-06	75.70	83.79	-8.09	0.86	0.75	0.97
cg18776460	LRIG3	12q14.1	4.29E-12	3.71E-06	71.92	80.74	-8.82	0.79	0.66	0.92
cg06793377	SHANK3	22q13.33	4.79E-12	4.14E-06	15.09	23.94	-8.85	0.82	0.71	0.94
cg02688948	KNDC1	10q26.3	5.18E-12	4.48E-06	85.55	91.41	-5.86	0.77	0.64	0.91
cg13263104	NF1	17q11.2	5.47E-12	4.73E-06	75.00	83.20	-8.20	0.89	0.79	0.98
cg13830799	GRM1	6q24.3	6.43E-12	5.56E-06	63.94	73.97	-10.03	0.87	0.77	0.98
cg25018881	IKBKAP	9q31.3	7.15E-12	6.19E-06	73.14	81.65	-8.51	0.86	0.75	0.97

cg01567825	DOCK4	7q31.1	8.07E-12	6.98E-06	63.29	73.36	-10.07	0.82	0.70	0.94
cg02891801	SCNN1A	12p13.31	8.75E-12	7.57E-06	12.63	6.56	6.07	0.84	0.72	0.95
cg02124957	VGLL4	3p25.3-p25.2	9.04E-12	7.82E-06	64.44	74.34	-9.89	0.94	0.86	1.00
cg09806966	WWP2	16q22.1	9.16E-12	7.92E-06	72.34	80.96	-8.62	0.90	0.80	0.99
cg15329467	CYP19A1	15q21.2	9.44E-12	8.16E-06	74.75	82.91	-8.16	0.95	0.88	1.00
cg21836418	PRDM16	1p36.32	9.51E-12	8.23E-06	92.05	96.17	-4.12	0.83	0.71	0.95
cg21663431	SLC44A2	19p13.2	1.19E-11	1.03E-05	71.67	80.37	-8.70	0.82	0.70	0.94
cg07707056	APOL3	22q12.3	1.28E-11	1.10E-05	73.04	81.48	-8.44	0.97	0.91	1.00
cg16260438	DNMT1	19p13.2	1.31E-11	1.14E-05	66.47	76.00	-9.53	0.87	0.76	0.97
cg04498110	CX3CR1	3p22.2	1.36E-11	1.18E-05	49.55	60.79	-11.23	0.85	0.74	0.96
cg18778658	EHMT2	6p21.33	1.60E-11	1.38E-05	65.76	75.36	-9.60	0.95	0.89	1.00
cg00696472	FAAH	1p33	1.94E-11	1.68E-05	81.89	88.46	-6.57	0.77	0.64	0.91
cg00888479	SLC24A3	20p11.23	2.10E-11	1.81E-05	13.54	7.38	6.16	0.88	0.77	0.98
cg14962136	SLC39A8	4q24	2.12E-11	1.83E-05	77.12	84.70	-7.57	0.81	0.68	0.93
cg00801568	TIPIN	15q22.31	2.18E-11	1.88E-05	18.84	28.13	-9.29	0.80	0.67	0.93
cg17969913	KCNQ5	6q13	2.34E-11	2.03E-05	44.02	55.30	-11.28	0.83	0.72	0.95
cg08122807	NRG1	8p12	2.50E-11	2.17E-05	69.90	78.77	-8.87	0.84	0.73	0.96
cg07782479	KIF1A	2q37.3	2.72E-11	2.35E-05	89.23	94.01	-4.77	0.81	0.68	0.93
cg03749900	PTN	7q33	3.02E-11	2.61E-05	67.04	76.33	-9.29	0.85	0.73	0.96
cg22496723	CACNG2	22q12.3	3.06E-11	2.65E-05	82.03	88.51	-6.48	0.77	0.64	0.91
cg10931900	AVPR1A	12q14.2	3.31E-11	2.87E-05	67.72	76.89	-9.17	0.89	0.79	0.98
cg00001534	FAF1	1p32.3	3.64E-11	3.15E-05	79.94	86.86	-6.91	0.89	0.80	0.99
cg00929860	SPOCK2	10q22.1	3.65E-11	3.16E-05	82.06	88.51	-6.45	0.76	0.62	0.90
cg07032258	ARNTL	11p15.3	3.74E-11	3.24E-05	22.97	15.23	7.74	0.80	0.67	0.92
cg12353802	SYN3	22q12.3	3.75E-11	3.24E-05	60.76	70.84	-10.08	0.92	0.84	1.00
cg05779523	MC2R	18p11.21	4.01E-11	3.47E-05	53.96	64.67	-10.71	0.88	0.77	0.98
cg05205813	CACNB2	10p12.33-p12.31	4.03E-11	3.49E-05	73.89	81.99	-8.10	0.96	0.91	1.00
cg10262747	ATXN1	6p22.3	4.05E-11	3.51E-05	68.06	77.14	-9.08	0.85	0.74	0.96
cg20539816	WSCD1	17p13.2	4.85E-11	4.20E-05	88.45	93.37	-4.92	0.78	0.64	0.91
cg04237075	SIGMAR1	9p13.3	5.26E-11	4.55E-05	9.48	4.26	5.22	0.89	0.80	0.99

cg12631766	TSEN15	1q25.3	5.44E-11	4.70E-05	61.30	71.24	-9.94	0.86	0.75	0.97
cg04270033	TUG1	22q12.2	5.48E-11	4.74E-05	67.91	76.96	-9.05	0.91	0.83	1.00
cg00381033	BBX	3q13.12	5.61E-11	4.85E-05	69.85	78.59	-8.74	0.80	0.67	0.93
cg04851617	RSU1	10p13	6.92E-11	5.99E-05	79.44	86.37	-6.93	0.84	0.73	0.95
cg05744073	MIR132	17p13.3	7.00E-11	6.05E-05	63.96	73.52	-9.56	0.92	0.84	1.00
cg15393297	GABRB1	4p12	7.06E-11	6.10E-05	76.06	83.67	-7.60	0.83	0.71	0.95
cg04573398	CCDC81	11q14.2	7.16E-11	6.19E-05	68.45	77.37	-8.92	0.80	0.67	0.93
cg20688157	NOTCH3	19p13.12	7.48E-11	6.47E-05	67.03	76.15	-9.12	0.92	0.84	1.00
cg00848336	MTA1	14q32.33	7.84E-11	6.78E-05	77.33	84.67	-7.34	0.81	0.69	0.94
cg13555519	GLIS3	9p24.2	9.39E-11	8.12E-05	70.13	78.73	-8.60	0.94	0.87	1.00
cg01265228	KDM2A	11q13.2	9.86E-11	8.53E-05	69.43	78.13	-8.70	0.82	0.71	0.94
cg02636348	KCNMA1	10q22.3	1.05E-10	9.06E-05	49.36	60.12	-10.76	0.92	0.84	1.00
cg07111008	KCND3	1p13.2	1.22E-10	0.000105488	72.26	80.46	-8.20	0.91	0.83	1.00
cg19154695	NOTCH4	6p21.32	1.22E-10	0.000105917	68.18	77.03	-8.86	0.90	0.81	0.99
cg15813570	MTHFD1	14q23.3	1.23E-10	0.000106681	11.72	6.13	5.60	0.86	0.76	0.97
cg06848802	TACR1	2p12	1.33E-10	0.000114655	80.75	87.32	-6.57	0.78	0.65	0.91
cg21774561	CSNK1D	17q25.3	1.46E-10	0.000126106	71.36	79.69	-8.32	0.86	0.75	0.97
cg26348243	LTA	6p21.33	1.52E-10	0.000131391	56.47	66.67	-10.21	0.78	0.65	0.91
cg21511036	IRS1	2q36.3	1.61E-10	0.000139089	12.39	6.69	5.70	0.82	0.70	0.94
cg08996726	MAML3	4q31.1	2.05E-10	0.000176971	65.09	74.28	-9.19	0.75	0.62	0.89
cg14219059	TEAD1	11p15.3	2.52E-10	0.000217609	66.90	75.81	-8.90	0.87	0.77	0.97
cg01368160	MAPK10	4q21.3	2.74E-10	0.000236708	66.78	75.69	-8.90	0.82	0.69	0.94
cg05856951	HMOX2	16p13.3	2.93E-10	0.000253469	69.60	78.07	-8.48	0.82	0.70	0.94
cg21556683	SCN5A	3p22.2	3.06E-10	0.000264461	73.36	81.21	-7.85	0.89	0.80	0.99
cg06474219	POLR1C	6p21.1	3.18E-10	0.000275296	62.67	72.07	-9.41	0.77	0.63	0.90
cg05061886	MN1	22q12.1	3.33E-10	0.000287943	76.74	83.97	-7.23	0.87	0.77	0.97
cg17244098	TNFRSF1B	1p36.22	3.36E-10	0.000290912	67.77	76.49	-8.72	0.92	0.84	1.00
cg23426002	BDNF	11p14.1	3.51E-10	0.000303592	66.08	75.03	-8.95	0.92	0.84	1.00
cg08133699	MMP2	16q12.2	4.07E-10	0.000351644	76.19	83.49	-7.30	0.79	0.66	0.92
cg00140361	CLIC1	6p21.33	4.10E-10	0.000354815	79.98	86.55	-6.57	0.94	0.87	1.00

cg15951188	KCNAB3	17p13.1	4.40E-10	0.000380823	72.45	80.39	-7.94	0.77	0.64	0.91
cg16792002	MAML2	11q21	4.67E-10	0.000403629	68.29	76.87	-8.58	0.78	0.65	0.92
cg18880390	KIAA0040	1q25.1	5.41E-10	0.000467874	9.97	4.89	5.08	0.87	0.76	0.97
cg25485084	TAOK3	12q24.23	7.04E-10	0.000608916	11.68	6.29	5.39	0.80	0.67	0.92
cg14847514	TPH2	12q21.1	7.15E-10	0.000618684	80.16	86.61	-6.45	0.86	0.75	0.97
cg16237565	PCSK6	15q26.3	8.69E-10	0.000751837	74.75	82.18	-7.43	0.80	0.67	0.92
cg07307789	FGF6	12p13.32	9.08E-10	0.000785197	72.22	80.07	-7.85	0.88	0.78	0.98
cg16625916	CTNNA2	2p12	9.21E-10	0.000796945	91.76	95.59	-3.83	0.85	0.73	0.96
cg27484541	GNAS	20q13.32	9.26E-10	0.000800628	70.90	78.96	-8.05	0.87	0.76	0.97
cg07368817	TRPV2	17p11.2	9.41E-10	0.00081403	9.86	4.86	5.00	0.91	0.82	1.00
cg19787508	SPTLC2	14q24.3	1.04E-09	0.000902239	74.26	81.74	-7.48	0.84	0.73	0.95
cg05224770	IFNG	12q15	1.17E-09	0.001016023	51.09	61.17	-10.08	0.84	0.72	0.95
cg11794440	CLCN6	1p36.22	1.21E-09	0.001050414	76.91	83.89	-6.99	0.92	0.84	1.00
cg01690182	GABRB3	15q12	1.24E-09	0.001068363	16.87	10.62	6.25	0.76	0.63	0.90
cg21881330	SLC24A4	14q32.12	1.31E-09	0.001134014	69.29	77.52	-8.23	0.85	0.73	0.96
cg11132979	GFRA2	8p21.3	1.41E-09	0.001215377	79.85	86.26	-6.41	0.91	0.82	1.00
cg17047033	C8orf34	8q13.2	1.41E-09	0.001221192	69.29	77.51	-8.22	0.77	0.64	0.91
cg13001097	UTRN	6q24.2	1.44E-09	0.0012476	77.86	84.65	-6.78	0.84	0.73	0.95
cg00925831	SVEP1	9q31.3	1.46E-09	0.001260712	49.51	59.61	-10.10	0.80	0.68	0.93
cg23688111	TRPM8	2q37.1	1.47E-09	0.001269164	49.57	59.66	-10.09	0.80	0.68	0.93
cg25228562	PRKCA	17q24.2	1.52E-09	0.001317608	69.17	77.39	-8.22	0.82	0.70	0.94
cg20187047	SYNE1	6q25.2	1.52E-09	0.001318705	74.48	81.86	-7.38	0.94	0.86	1.00
cg20549250	OPRK1	8q11.23	1.55E-09	0.001339293	83.15	88.88	-5.73	0.89	0.79	0.98
cg04509559	SLC39A9	14q24.1	1.59E-09	0.001374679	23.02	15.87	7.16	0.75	0.62	0.89
cg08251685	NPSR1	7p14.3	1.60E-09	0.001386991	75.77	82.92	-7.14	0.90	0.80	0.99
cg13195164	MAP2K1	15q22.31	1.72E-09	0.001483661	73.41	80.95	-7.54	0.80	0.67	0.92
cg25204926	LPAR5	12p13.31	1.92E-09	0.001657102	76.39	83.39	-7.00	0.92	0.84	1.00
cg13062137	NLGN2	17p13.1	2.05E-09	0.001769208	80.00	86.32	-6.32	0.90	0.81	0.99
cg15229027	ESR2	14q23.2-q23.3	2.10E-09	0.001818778	10.76	5.68	5.08	0.85	0.74	0.96
cg16777493	ATP2C2	16q24.1	2.27E-09	0.001963617	73.38	80.87	-7.49	0.96	0.91	1.00

cg09243909	FTO	16q12.2	2.27E-09	0.001967836	71.30	79.12	-7.82	0.88	0.78	0.98
cg19990483	ATL1	14q22.1	2.65E-09	0.002293402	65.91	74.46	-8.55	0.76	0.62	0.89
cg03534360	SYT16	14q23.2	2.71E-09	0.002344868	78.85	85.34	-6.50	0.88	0.78	0.98
cg24426733	ZSCAN20	1p35.1	2.83E-09	0.00244981	91.02	94.92	-3.91	0.84	0.73	0.96
cg21236655	TNC	9q33.1	2.89E-09	0.002497346	73.55	80.97	-7.42	0.85	0.74	0.96
cg16420354	NOS3	7q36.1	2.96E-09	0.002558065	66.48	74.93	-8.45	0.92	0.84	1.00
cg13659446	MRC2	17q23.2	3.15E-09	0.002722309	12.47	7.12	5.35	0.79	0.66	0.92
cg17582698	DGKI	7q33	3.22E-09	0.002784083	62.29	71.22	-8.93	0.80	0.68	0.93
cg03511766	CHRM3	1q43	3.24E-09	0.002806765	67.71	75.98	-8.27	0.85	0.74	0.96
cg21453309	FAM101A	12q24.31	3.38E-09	0.002924058	79.32	85.69	-6.37	0.91	0.83	1.00
cg11295357	HTRA1	10q26.13	3.52E-09	0.003047539	59.34	68.55	-9.21	0.80	0.67	0.92
cg15216232	IL12B	5q33.3	3.52E-09	0.003048918	69.98	77.91	-7.93	0.84	0.73	0.95
cg23329581	RUNX1	21q22.12	3.82E-09	0.00330226	80.16	86.35	-6.20	0.86	0.76	0.97
cg04920270	NCAM1	11q23.2	3.95E-09	0.003414943	9.75	4.94	4.81	0.77	0.64	0.91
cg06398735	MLLT10	10p12.31	4.14E-09	0.003579461	14.22	21.48	-7.26	0.76	0.62	0.89
cg15519237	PRX	19q13.2	4.62E-09	0.003994682	79.55	85.83	-6.28	0.75	0.62	0.89
cg25104727	ASTN1	1q25.2	5.45E-09	0.00471836	51.76	61.41	-9.65	0.77	0.63	0.90
cg14074830	TSSC1	2p25.3	5.55E-09	0.00480267	87.38	92.04	-4.66	0.77	0.64	0.91
cg25121146	MC4R	18q21.32	6.10E-09	0.005279088	63.79	72.40	-8.62	0.79	0.66	0.92
cg26419659	FAM134B	5p15.1	6.26E-09	0.005412003	63.02	71.72	-8.70	0.81	0.69	0.94
cg15258981	TRPM2	21q22.3	7.48E-09	0.006469856	84.27	89.55	-5.28	0.84	0.73	0.96
cg00510552	NMRAL1	16p13.3	7.56E-09	0.006538352	87.73	92.28	-4.55	0.77	0.63	0.90
cg11327857	FGF2	4q28.1	7.60E-09	0.006575225	39.04	48.79	-9.74	0.75	0.61	0.89
cg22352186	TACC3	4p16.3	7.69E-09	0.006653765	11.99	6.84	5.15	0.92	0.83	1.00
cg14938410	ADAMTSL4	1q21.2	7.83E-09	0.006776056	10.32	5.48	4.84	0.88	0.78	0.98
cg01788910	CBS	21q22.3	7.92E-09	0.006852629	67.60	75.69	-8.09	0.76	0.63	0.90
cg20807790	POMC	2p23.3	7.97E-09	0.006894593	72.96	80.28	-7.32	0.85	0.74	0.96
cg04365102	RAMP1	2q37.3	8.13E-09	0.007030841	47.65	57.35	-9.70	0.77	0.63	0.90
cg02027079	HTR2A	13q14.2	8.21E-09	0.007101469	65.11	73.51	-8.39	0.84	0.73	0.95
cg19420968	HCRTR1	1p35.2	8.58E-09	0.007420118	74.40	81.47	-7.07	0.88	0.78	0.98

cg11610346	PENK	8q12.1	9.78E-09	0.008461104	13.15	7.82	5.32	0.80	0.67	0.92
cg05232889	FOXP2	7q31.1	1.03E-08	0.008949066	54.46	63.78	-9.32	0.86	0.75	0.97
cg06500883	ADAMTSL1	9p22.2-p22.1	1.04E-08	0.008992357	78.89	85.16	-6.27	0.94	0.87	1.00
cg17522897	WSCD2	12q23.3	1.05E-08	0.00905492	63.99	72.46	-8.47	0.85	0.74	0.96
cg08249385	TSC2	16p13.3	1.18E-08	0.010218458	70.43	78.05	-7.62	0.93	0.86	1.00
cg07576222	SMAD3	15q22.33	1.19E-08	0.010266692	39.64	49.27	-9.63	0.79	0.66	0.92
cg10769507	PIK3C2G	12p12.3	1.19E-08	0.010332797	67.58	75.58	-8.00	0.92	0.84	1.00
cg18143869	P2RY12	3q25.1	1.23E-08	0.010635691	60.74	69.51	-8.77	0.77	0.64	0.91
cg05402976	PRKG1	10q11.23-q21.1	1.23E-08	0.010654573	78.26	84.61	-6.35	0.79	0.66	0.92
cg01845051	ULK4	3p22.1	1.24E-08	0.010698496	82.99	88.46	-5.47	0.75	0.62	0.89
cg01846046	PLCB3	11q13.1	1.25E-08	0.010847387	85.85	90.74	-4.89	0.83	0.71	0.95
cg11175977	TLL2	10q24.1	1.32E-08	0.011402174	76.60	83.23	-6.63	0.95	0.89	1.00
cg02587120	HDC	15q21.2	1.34E-08	0.0116084	74.09	81.13	-7.04	0.83	0.71	0.95
cg22895463	VEPH1	3q25.31-q25.32	1.43E-08	0.012401794	72.08	79.42	-7.33	0.81	0.69	0.94
cg00230450	C12orf60	12p13.1-p12.3	1.46E-08	0.012600076	72.87	80.08	-7.21	0.79	0.66	0.92
cg19859993	IL19	1q32.1	1.46E-08	0.012649902	64.70	73.01	-8.31	0.84	0.72	0.95
cg15724876	TGFBR2	3p24.1	1.47E-08	0.01273597	72.61	79.86	-7.25	0.79	0.66	0.92
cg14692920	RAPH1	2q33.2	1.52E-08	0.013132424	69.66	77.33	-7.67	0.76	0.63	0.90
cg22449980	PDGFC	4q32.1	1.69E-08	0.014610662	71.15	78.58	-7.44	0.77	0.64	0.91
cg12078872	DDO	6q21	1.86E-08	0.016051899	70.46	77.98	-7.52	0.90	0.81	0.99
cg18731202	TGFA	2p13.3	1.86E-08	0.016130772	61.85	70.41	-8.56	0.75	0.61	0.89
cg00712106	NRIP1	21q11.2-q21.1	1.93E-08	0.016658466	76.09	82.73	-6.64	0.83	0.71	0.95
cg18422371	RAG1	11p12	2.01E-08	0.017357929	49.94	59.31	-9.37	0.80	0.67	0.92
cg17154602	GALR1	18q23	2.08E-08	0.017985359	64.97	73.17	-8.19	0.80	0.68	0.93
cg24458329	CASP9	1p36.21	2.09E-08	0.01809463	87.77	92.19	-4.42	0.87	0.76	0.97
cg23019936	GRIN2B	12p13.1	2.10E-08	0.01817917	59.10	67.88	-8.78	0.81	0.68	0.93
cg11464873	ENPP1	6q23.2	2.15E-08	0.01858432	73.56	80.58	-7.03	0.86	0.76	0.97
cg08649765	EREG	4q13.3	2.31E-08	0.019994478	69.51	77.11	-7.60	0.83	0.71	0.95
cg26815617	FAM183B	7p14.1	2.50E-08	0.021589692	76.08	82.68	-6.60	0.79	0.66	0.92
cg10002977	SOD2	6q25.3	2.58E-08	0.022292836	11.62	6.70	4.92	0.81	0.69	0.94

cg16378117	GRIN2A	16p13.2	2.58E-08	0.022315179	67.40	75.25	-7.85	0.86	0.75	0.97
cg05081694	SCN3A	2q24.3	2.70E-08	0.023388477	68.07	75.83	-7.75	0.76	0.62	0.89
cg03043834	RGS12	4p16.3	2.72E-08	0.02349423	81.90	87.46	-5.56	0.87	0.77	0.97
cg17219740	GRM7	3p26.1	2.83E-08	0.024512397	69.62	77.16	-7.54	0.89	0.80	0.99
cg08253824	SCN8A	12q13.13	2.84E-08	0.024529457	72.83	79.91	-7.08	0.76	0.63	0.90
cg01199408	MRVI1	11p15.4	2.88E-08	0.024907465	72.26	79.42	-7.16	0.87	0.76	0.97
cg24182584	CYP1A2	15q24.1	3.02E-08	0.026095051	88.05	92.36	-4.32	0.80	0.68	0.93
cg12023999	C17orf67	17q22	3.07E-08	0.026531927	61.28	69.77	-8.49	0.91	0.82	1.00
cg16895486	ADRA1A	8p21.2	3.35E-08	0.028999533	54.47	63.47	-9.00	0.83	0.71	0.95
cg24542230	RNF213	17q25.3	3.39E-08	0.02932653	84.94	89.88	-4.94	0.84	0.73	0.96
cg13313430	ICA1	7p21.3	3.40E-08	0.029399725	77.03	83.41	-6.38	0.79	0.66	0.92
cg12742785	CAPN1	11q13.1	3.48E-08	0.03013891	72.16	79.30	-7.14	0.88	0.78	0.98
cg08309747	SPON1	11p15.2	3.61E-08	0.031255374	66.44	74.32	-7.89	0.76	0.62	0.89
cg13690943	UBAP2	9p13.3	3.63E-08	0.031402148	75.84	82.40	-6.56	0.90	0.81	0.99
cg27155504	SLC25A3	12q23.1	4.03E-08	0.034844538	67.64	75.36	-7.72	0.79	0.66	0.92
cg05511685	RGS9	17q24.1	4.11E-08	0.035580973	77.81	84.02	-6.22	0.88	0.78	0.98
cg17373716	SCN11A	3p22.2	4.44E-08	0.038377256	79.67	85.55	-5.88	0.91	0.82	1.00
cg00660272	CNR2	1p36.11	5.03E-08	0.043467555	14.14	8.88	5.25	0.79	0.66	0.92
cg17583957	MAP2K6	17q24.3	5.43E-08	0.046927144	71.57	78.70	-7.13	0.81	0.68	0.93
cg03509899	FGF3	11q13.3	5.44E-08	0.047026433	20.55	14.35	6.20	0.78	0.65	0.91
cg10036840	C7orf10	7p14.1	5.50E-08	0.047582856	74.54	81.23	-6.68	0.77	0.64	0.91
cg01670789	BEGAIN	14q32.2	5.53E-08	0.047861566	72.32	79.34	-7.01	0.84	0.73	0.95
