# Supplementary material for: Impaired Molecular Mechanisms Contributing to Chronic Pain in Patients with Hidradenitis Suppurativa: Exploring Potential Biomarkers and Therapeutic Targets
Source: Int J Mol Sci. 2025 Jan 25;26(3):1039. doi: 10.3390/ijms26031039 (PMC11817842; doi:10.3390/ijms26031039)
Supplement: Supplementary file 1 [file ijms-26-01039-s001.zip › Supplementary Table S2.pdf]

Supplementary Table S2. Comprehensive list of pain-associated genes retrieved from published data and relevant references, compared with findings from our study.

Gene Pain associated	Reference
ABAT	PMID:30277654
ABCB1	PMID:31755291
ABCB1B	
ABCC2	PMID:22454423
ABCC4	PMID:22454423
ABCG1	
AC005592.2	PMID:34450027
AC005592.3	PMID:34450027
ACAN	PMID:28742099
ACE	PMID:19298544
ACE2	PMID:24275226
ACO1	PMID:25144566
ADAMTS4	PMID:28081267
ADAMTS5	PMID:28081267
ADAMTSL1	PMID:22678113
ADAMTSL4	
ADARB2	PMID:22678113
ADCYAP1	
ADCYAP1R1	PMID:25169732
ADH1B	PMID:30371560
ADORA1	
ADORA2A	
ADORA2A-AS1	PMID:17257240
ADORA3	
ADRA1A	PMID:19565482
ADRA1D	PMID:30904518
ADRA2A	PMID:27805929
ADRA2C	
ADRB1	PMID:26694722
ADRB2	PMID:31285095
AGTR2	
AHR	
AJAP1	
ALDH1A2	PMID:34450027
ALOX12	
AMIGO3	PMID:32587327
ANAPC4	PMID:31194737

ANKH	
ANKK1	PMID:22875483
ANKTM1	
ANRIL	
AOC1	PMID:34450027
APOA1BP	
APOE	PMID:21453125
APOL3	
APP	
AQP2	PMID:32130259
AQP4	
AQP5-AS1	PMID:32130259
AQPEP	PMID:34450027
AR	
ARID4A	PMID:32632093
ARL14EP	PMID:32121467
ARL14EP-DT	PMID:32121467
ARMC12	PMID:23707272
ARMS2	
ARNTL	
ARRB2	PMID:23167532
ARVCF	PMID:31285095
ASIC1	
ASIC3	
ASTN1	PMID:22678113
ASTN2	PMID:34450027
ATAT1	
ATF4	
ATG5	
ATL1	
ATP1A2	
ATP2C1	PMID:33926923
ATP2C2	PMID:18676988
ATP5B	
ATXN1	PMID:18403122
AVIL	
AVPR1A	PMID:24373571
B2M	PMID:25144566
BACE1	
BAHCC1	PMID:28081371
BBX	PMID:31194737

BDKRB1	
BDKRB2	
BDNF	PMID:32736598
BDNF-AS	PMID:19517061
BECN1	
BEGAIN	
BHLHA9	
BMP5	PMID:34450027
BMP6	PMID:34450027
BRWD1	PMID:34450027
BUD23	PMID:22250207
C12orf60	PMID:34450027
C12orf65	PMID:34450027
C17orf67	PMID:34450027
C1orf177	PMID:34450027
C21orf91	
C2orf40	PMID:34450027
C2orf41	PMID:34450027
C6orf106	PMID:33830993
C7orf10	
C7orf50	PMID:25169732
C8orf34	PMID:34450027
CA10; snoZ178	PMID:33830993
CACNA1A	PMID:19429006
CACNA1B	
CACNA1C	
CACNA1E	PMID:28573794
CACNA1F	
CACNA1H	
CACNA1I	
CACNA1S	
CACNA2D2	PMID:23402298
CACNA2D3	PMID:22678113
CACNB2	PMID:18676988
CACNB3	
CACNG2	PMID:30371558
CALCA	PMID:29148033
CALCRL	PMID:25169732
CAMK2A	
CAMK4	PMID:22074755
CAMKIV	

CAPN1	
CARF	PMID:32046629
CASC16	PMID:31706190
CASP9	
CBR3-AS1	PMID:31056713
CBS	PMID:21402364
CCDC190	PMID:21905019
CCDC81	
CCKBR	
CCL2	
CCM2L	
CCR2	PMID:19559392
CCT5	
CD247	PMID:26872611
CDCA3	PMID:20102604
CDH12	PMID:22678113
CDH18	PMID:28051079
CDK5R1	
CDKL5	
CEP120	PMID:31194737
CFAP418-AS1	PMID:31903573
CFDP1	PMID:32632093
CFTR	
CGRP	
CHRM2	PMID:24275226
CHRM3	PMID:21570824
CHRNA1	PMID:22678113
CHRNA3	PMID:31362771
CHRNA5	PMID:31362771
CHST3	PMID:34450027
CLCN6	
CLIC1	
CLIC4	
CLIC5	PMID:18676988
CLOCK	PMID:25169732
CN5A	
CNR1	PMID:19539700
CNR2	
COL11A1	PMID:34450027
COL27A1	PMID:34450027
COL4A1	PMID:32632093

COLGALT2	PMID:34450027
COMT	PMID:22718527
COX1	
COX2	
CP	PMID:25144566
CPQ	
CRADD	PMID:34450027
CRHBP	PMID:26447706
CRHR2	PMID:22074755
CRIP2	
CSF1	
CSF2	
CSNK1D	
CTC-537E7.1	PMID:34450027
CTD-2334D19.1;AC008565.1	PMID:33830993
CTD-2623N2.11	PMID:34450027
CTNNA2	PMID:31194737
CTSB	
CTSG	PMID:26270939
CTSS	
CUX1	PMID:34450027
CX3CL1	
CX3CR1	
CXCL8	
CYP19A1	PMID:22511967
CYP1A2	PMID:20652353
CYP2B6	PMID:31056713
CYP2D6	
CYP3A4	PMID:30381583
DAN	
DAO	
DBH	PMID:19152006
DBH-AS1	PMID:26868704
DCC	PMID:30261039
DCDC1	PMID:29995844
DDO	
DGKI	PMID:34450027
DICER1	
DLG2	
DMD	PMID:28081371

DMPIEZO	
DNAJA3	PMID:32632093
DNMT1	
DOCK4	
DPP4	
DRD1	
DRD2	PMID:31144779
DRD3	PMID:19464960
DRD4	
DYNC1I1	PMID:31194737
EAR2	
ECM1	PMID:32587327
EDN1	
EDNRA	PMID:25169732
EDNRB	PMID:19661472
EFNB1	
EFNB2	PMID:33021770
EGR1	
EHBP1L1	PMID:34450027
EHBP1L2	PMID:34450027
EHMT2	
EIF2	
EIF2AK2	
EIF2AK3	
EIF4E	
EIF4EBP1	
ELAC2	PMID:21402364
ENPP1	PMID:27519661
ENT1	
EREG	PMID:24275226
ERG	PMID:34450027
ESR1	PMID:24698360
ESR2	PMID:19093296
ESRRB	PMID:26584852
EXD3	PMID:33830993
EXT2	
F2	PMID:25158985
F2RL1	
F5	PMID:25158985
FAAH	PMID:31335650
FABP3P2	PMID:34450027

FAF1	PMID:33830993
FAF2	PMID:32587327
FAM101A	PMID:34450027
FAM134B	
FAM183B	
FANCL	PMID:34450027
FGF10	PMID:31574782
FGF13	PMID:31574782
FGF18	PMID:34450027
FGF2	
FGF3	PMID:31574782
FGF6	
FHL5	PMID:32632093
FILIP1	PMID:34450027
FKBP5	PMID:30150364
FNDC3B	PMID:31903573
FOXP2	PMID:31194737
FPR1	
FRMD4A	
FSHR	PMID:19093296
FSTL4	
FTO	PMID:34450027
FUT9	
GABBR1	
GABRA3	PMID:24040174
GABRB1	PMID:17655760
GABRB2	PMID:31194737
GABRB3	
GABRG2	
GABRR1	PMID:28699326
GAL	
GALR1	
GALR2	
GAS5	
GBP1	
GCH1	PMID:17057711
GDF5	PMID:34450027
GDF6	PMID:34450027
GDF7	PMID:34450027
GFAP	
GFRA2	

GJA1	
GLIS3	PMID:34450027
GLIS4	PMID:34450027
GNA11	
GNAO1	
GNAQ	
GNAS	PMID:17388805
GNAZ	
GPD2	PMID:28051079
GPR149	
GPR34	
GPR55	
GRIA1	PMID:32046629
GRIA3	PMID:24275226
GRIN1	
GRIN2A	PMID:24275226
GRIN2B	
GRK2	
GRK3	PMID:30904518
GRK5	PMID:24275226
GRM1	
GRM5	
GRM7	PMID:22678113
GSDMC	PMID:30747904
GSTM1	
H1F0	PMID:34450027
H2TRA	
HAMLET	
HCN2	
HCRTR1	PMID:21344296
HCRTR2	PMID:26289589
HDAC9	PMID:34450027
HDC	
HEY2	
HFE	PMID:34450027
HIC1	PMID:32632093
HLA-B	
HLA-DPA1	PMID:34450027
HLA-DQ	
HLA-DRB1	
HMOX2	

HOTAIR	
HOXB8	
HPSE2	PMID:32632093
HRH1	
HRH2	
HSN2	
HTR1A	PMID:30904518
HTR2A	PMID:33171011
HTR2C	PMID:21614492
HTR3A	PMID:30904518
HTR3B	PMID:21570824
HTR7	
HTRA1	PMID:30747904
IAPP	
ICA1	
ICA1L	PMID:32632093
ICAM1	PMID:25145994
IFNG	PMID:28651128
IFNGR	
IFRD1	PMID:24275226
IGSF9B	
IKBKAP	
IL10	PMID:22074755
IL10R1	
IL10RB	PMID:23852407
IL11	PMID:34450027
IL12B	PMID:32620160
IL13	PMID:25304131
IL16	PMID:20662556
IL18R1	
IL18RAP	PMID:23522322
IL19	PMID:29636026
IL1A	PMID:28081267
IL1B	PMID:27649267
IL1R1	PMID:22515947
IL1R2	PMID:24411993
IL1RN	PMID:25207923
IL23	
IL23R	
IL37	PMID:23317890
IL4	PMID:32141366

IL6	PMID:27048515
IL6-AS1	PMID:15733644
IL6ST	
IL8	
IL9	PMID:19559392
ILRUN	PMID:31194737
INSR	PMID:18455362
IQGAP1	PMID:31903573
IRAG1	PMID:32632093
IRF2BP1	PMID:34450027
IRS1	
ITGB2	
ITGB5	PMID:32632093
ITIH1	PMID:34450027
ITIH2	PMID:34450027
ITPK1	
JAG1	
JAKMIP3	PMID:30747904
KCNA1	PMID:25599232
KCNA2	
KCNAB3	
KCNB2	PMID:18676988
KCND3	PMID:31194737
KCNG4	PMID:32697988
KCNJ2	
KCNJ3	PMID:25599232
KCNJ6	PMID:31269327
KCNK18	
KCNK2	PMID:24275226
KCNK5	PMID:27322543
KCNK9	PMID:25599232
KCNMA1	PMID:34450027
KCNN3	PMID:22030984
KCNQ2	
KCNQ3	PMID:18676988
KCNQ4	
KCNQ5	
KCNS1	PMID:24392765
KDM2A	PMID:34450027
KDR	PMID:31118800
KIAA0040	PMID:32632093

KIF1A	
KLF11	
KNDC1	PMID:31194737
LAMB3	PMID:26566055
LDLR	
LEMD2	PMID:33830993
LEP	
LEPREL1	PMID:34450027
LINC00342	PMID:32632093
LINC00568;RP11-54A4.2	PMID:33830993
LINC00842	PMID:31903573
LINC01347	PMID:31194737
LINC01572	PMID:32246137
LINC02029	PMID:31903573
LMX1B	
LOC100287329	PMID:30129153
LOC100506	
LOC101448202	PMID:25896984
LOC101926964	PMID:31903573
LOC101927025	PMID:31903573
LOC101927066	PMID:22103325
LOC101927995	PMID:27322543
LOC101929309	PMID:23707272
LOC102724058	PMID:24275226
LOC105369501	PMID:22875483
LOC105369944	PMID:25724697
LOC105370032	PMID:29470314
LOC105370955	PMID:21448238
LOC105371394	PMID:28051079
LOC105371720	PMID:27043930
LOC105371818	PMID:28081371
LOC105372112	PMID:21448238
LOC105373313	PMID:15147464
LOC105373370	PMID:24040174
LOC105373786	PMID:25169732
LOC105373891	PMID:31903573
LOC105375078	PMID:18676988
LOC105375350	PMID:19368856
LOC105375457	PMID:22074755
LOC105375567	PMID:25612138
LOC105375629	PMID:23793025

LOC105375630	PMID:25388962
LOC105375655	PMID:24674449
LOC105375836	PMID:22730276
LOC105375897	PMID:18676988
LOC105376225	PMID:32218487
LOC105376360	PMID:22678113
LOC105377013	PMID:32046629
LOC105377703	PMID:28051079
LOC105377864	PMID:17417740
LOC105377951	PMID:21622719
LOC105377986	PMID:32046629
LOC105378525	PMID:32046629
LOC105378606	PMID:32046629
LOC105378841	PMID:21905019
LOC105379109	PMID:31194737
LOC105379318	PMID:24974787
LOC107985507	PMID:25599232
LOC107986832	PMID:33021770
LOC107986931	PMID:22683712
LOC112267867	PMID:26872611
LOC112267956	PMID:23707272
LOC112268294	PMID:16960721
LOC349160	PMID:22074755
LPAR1	
LPAR5	
LPP	PMID:34450027
LRFN5	PMID:31748543
LRIG3	PMID:34450027
LRP1	PMID:32632093
LRRIQ3	
LTA	PMID:24959879
LTBP1	PMID:34450027
LTBP2	PMID:34450027
MACC1-AS1	PMID:21448238
MAKP14	
MALAT1	
MAML2	PMID:34450027
MAML3	PMID:31194737
MAOA	
MAOB	
MAOB	PMID:16807522

MAP1LC3B	
MAP2K1	PMID:23867732
MAP2K6	PMID:34450027
MAPK1	PMID:26872611
MAPK10	
MAPK14	
MAPK3	
MAPK8	
MAPK9	
MARCHF4	PMID:22683712
MC1R	
MC2R	
MC4R	
MDK	
MECP2	
MED14	
MEF2D	PMID:32632093
MEFV	PMID:23010357
MGC4859	PMID:29486785
miR1	
miR124	
miR132	
miR16	
miR183	
miR195	
miR199a-3p	
miR200	
miR206	
miR21	
miR212	
miR221	
miR222	
miR23a/b	
miR29	
miR30	
miR339-3p	
miR34	
miR34a	
miR34b	
miR365	
miR431	

miR451	
MIR4713HG	PMID:22511967
miR499	
miR504	
miR551b-3p	
miR7	
miR9	
MKK3	
MKK6	
MLLT10	PMID:31194737
MME	
MMP1	
MMP13	
MMP16	
MMP17	PMID:22683712
MMP2	
MMP24	
MMP3	
MMP9	PMID:31455415
MN1	PMID:34450027
MNK1	
MNK2	
MNSOD	
MPDZ	PMID:24275226
MPPED2	PMID:32632093
MPZ	
MRC2	
MRGPRE	
MRVI1	
MSC-AS1	PMID:24752136
MSH2	PMID:18676988
MTA1	
MTDH	PMID:21448238
MTHFD1	PMID:21615938
MTHFR	PMID:11121176
MTHR	
MTR	PMID:23430981
MTRR	PMID:21615938
MYD88	PMID:26332828
MYT1L	PMID:24582949
N4BP1	

NA	PMID:33830993
NACA2	PMID:34450027
NBR1	
NCAM1	PMID:30747904
NCOR2	PMID:32632093
NDN	
NEAT1	
NEFM	PMID:34450027
NF1	PMID:22678113
NFKB1A	
NFKBIA	PMID:19773451
NGF	
NGF-AS1	PMID:22074755
NGFB	
NGFR	PMID:21448238
NGR2	
NLGN2	
NMRAL1	PMID:34450027
NMT1	PMID:31194737
NNMT	PMID:27726107
NOG	PMID:34450027
NOS1	PMID:22074755
NOS2	PMID:22234503
NOS3	PMID:26098763
NOTCH3	PMID:20813781
NOTCH4	
NPM1; FGF18	PMID:33830993
NPSR1	PMID:25091462
NPSR1-AS1	PMID:25091462
NPTX1	
NPY	
NPY1R	
NR3C1	PMID:22074755
NRG1	
NRIP1	PMID:25315199
NRXN3	
NSRP1	PMID:19845785
NTRK1	PMID:23223113
NTSR1	PMID:24275226
NTSR2	
NUMB	PMID:31194737

OPRD1	PMID:28084056
OPRK1	PMID:29120944
OPRM1	PMID:12357145
OSM	
OXR1	
OXT	
P2RX3	
P2RX4	
P2RX7	PMID:24934217
P2RY12	
P2X3	
PACERR	PMID:23357220
PANX1	
PARD6G	PMID:34450027
PATJ	PMID:24275226
PATL2	PMID:25144566
PAX5,LOC105376032	PMID:29995844
PCP2	PMID:29884837
PCSK5	
PCSK6	PMID:22440827
PDE10A	
PDGFC	PMID:26872611
PDYN	
PDYN-AS1	PMID:22730276
PENK	
PER2	
PGAM1P1;PLK2	PMID:33830993
PGK1	
PGR	PMID:25494303
PHACTR1	PMID:32632093
PIK3C2G	PMID:26872611
PIK3C3	_
PIP5K1C	
PIRT	
PJA2	PMID:31903573
PLCB1	
PLCB3	
PLCE1	PMID:32632093
PLP1	
PNOC	
PNPLA3	

POC5	PMID:32632093
POLD3	PMID:34450027
POLD4	PMID:34450027
POLE	PMID:24275226
POLR1C	PMID:20482220
POMC	PMID:19723618
PON1	PMID:20407783
PPARA	
PPP1R14C	
PRDM12	
PRDM16	PMID:32632093
PRKAR1B	
PRKCA	PMID:28051079
PRKCA	
PRKCB	
PRKCD	
PRKCG	
PRKCG	
PRKCQ	
PRKCZ	
PRKG1	
PRKN	PMID:22993228
PRLR	
PROK2	
PROKR1	
PRRT2	
PRRX1	
PRSS1	
PRX	
PTGER1	
PTGER3	
PTGIR	
PTGS1	PMID:24275226
PTGS2	PMID:26081267
PTN	
PTPN5	
PTPRJ	PMID:34450027
RAB7A	
RAG1	
RALGPS1	PMID:34450027
RAMP1	PMID:25169732

RAPH1	PMID:34450027
REST	
RETN	PMID:29649030
RGS12	PMID:22683712
RGS9	
RHBDF2	
RLUA-1	
RLUA-2	
RNF123	PMID:31194737
RNF144B	PMID:34450027
RNF213	PMID:32632093
RNU2-17P	PMID:34450027
RNU2-40P	PMID:34450027
RNU6-815P	PMID:34450027
RNU6-962P	PMID:34450027
RNU6-996P	PMID:34450027
RORA	PMID:29884837
RP11-115J23.1	PMID:34450027
RP11-123K19.1	PMID:34450027
RP11-274M4.1	PMID:34450027
RP11-281A20.2	PMID:34450027
RP11-284G10.1	PMID:34450027
RP11-290F24.3	PMID:33830993
RP11-332M4.1	PMID:34450027
RP11-35O15.1	PMID:34450027
RP11-501E14.1	PMID:34450027
RP11-95P13.1	PMID:34450027
RP11-993B23.1	PMID:34450027
RP11-993B23.2	PMID:34450027
RP11-993B23.3	PMID:34450027
RP1-228P16.4	PMID:34450027
RPL19P11	PMID:34450027
RSU1	PMID:31903573
RTP4	PMID:34450027
RUNX1	
RUNX2	PMID:34450027
S100A10	
SARM1	
SCH1	
SCN10A	PMID:31642403
SCN11A	PMID:28953656

SCN12A	
SCN1A	PMID:24275226
SCN1A-AS1	PMID:20212137
SCN2A	
SCN3A	
SCN4A	
SCN5A	PMID:18676988
SCN7A	
SCN8A	
SCN9A	PMID:26752484
SCN9A,SCN1A-AS1	PMID:26752484
SCNN1A	PMID:25169732
SDK1	PMID:31194737
SEPT9	
SERPINA1	PMID:34450027
SERPINA6	PMID:19723618
SFRP1	PMID:30431558
SHANK3	
SHMT1	PMID:21615938
SIGMAR1	PMID:30266269
SLC10A7	PMID:33021770
SLC11A2	PMID:25144566
SLC12A2	
SLC12A5	
SLC17A6	
SLC17A8	PMID:22678113
SLC24A3	PMID:32632093
SLC24A3;Â AL121761.1	PMID:33830993
SLC24A4	
SLC25A3	PMID:34854908
SLC27A6	PMID:34450027
SLC2A1	
SLC39A8	PMID:34450027
SLC39A9	PMID:34450027
SLC44A2	PMID:34450027
SLC6A2	PMID:29723560
SLC6A3	PMID:29723560
SLC6A4	PMID:30904518
SLCO1A2	
SMAD3	PMID:34450027
SMAD4	PMID:34450027

SMAL	
SMG6	PMID:34450027
SMO	PMID:34450027
SNAP25	PMID:24885975
SNAP47	PMID:34450027
SNX8	PMID:29884837
SOD2	PMID:25818327
SORCS3	PMID:31194737
SORT1	
SOX10	
SOX11	PMID:33830993
SOX5	PMID:34450027
SOX6	PMID:34450027
SP4	PMID:31194737
SPAG17	PMID:34450027
SPARC	
SPINK1	
SPOCK2	PMID:33021770
SPON1	PMID:21622719
SPP1	
SPTLC1	
SPTLC2	
SQSTM1	
ST8SIA1	
STAG1	PMID:31194737
STAT6	PMID:23793025
STX1A	PMID:25169732
SUGCT	PMID:32632093
SUV39H2	PMID:26220684
SVEP1	PMID:22678113
SYN3	PMID:28081267
SYNE1	PMID:25315199
SYT16	PMID:25169732
TAAR1	PMID:21905019
TAAR2	PMID:21905019
TAC1	PMID:24275226
TAC4	
TACC3	PMID:34450027
TACR1	PMID:23167532
TAOK3	PMID:30031856
TBC1D7	

TBK1	
TCL1A	PMID:22405131
TCP11	PMID:34450027
TDAG8	
TEAD1	PMID:34450027
TF	PMID:25144566
TFRC	PMID:29351172
TG	PMID:28051079
TGFA	PMID:34450027
TGFB1	PMID:34450027
TGFB2	PMID:34450027
TGFBR2	
TH	PMID:30904518
THRB	
TIPIN	PMID:23867732
TLR2	PMID:27649267
TLR4	PMID:23023380
TMEM16F	
TMEM18	PMID:34450027
TMEM35A	
TNC	PMID:34450027
TNF	PMID:25315199
TNFA	
TNFRSF11B	PMID:26798969
TNFRSF1A	PMID:30075559
TNFRSF1B	PMID:30075559
TPH1	PMID:17194593
TPH2	PMID:20740293
TPSAN9	
TRMT9B	PMID:21666692
TRPA1	PMID:29620434
TRPC1	PMID:22162417
TRPC4	PMID:22162417
TRPC5	
TRPC7-AS2	PMID:22162417
TRPM2	
TRPM6	PMID:22162417
TRPM8	PMID:31873179
TRPV1	PMID:31014225
TRPV2	PMID:27079220
TRPV3	PMID:22162417

TRPV4	PMID:22162417
TSC2	
TSEN15	PMID:34450027
TSKU	PMID:34450027
TSPAN2	
TSPO	PMID:25582579
TSSC1	PMID:34924555
TUG1	
TXNRD2	PMID:22337325
UBAP2	PMID:34450027
UFL1-AS1	PMID:22683712
UGT2B7	PMID:31607718
ULK1	
ULK4	
USP8	PMID:34450027
USP9	PMID:34450027
UTRN	PMID:31194737
UTS2	PMID:27090416
VDR	PMID:23984350
VDR	PMID:29467039
VEPH1	PMID:25604633
VGLL4	PMID:34450027
VGLUT2	
WNK1	
WSCD1	
WSCD2	PMID:34450027
WWP2	PMID:32587327
XIST	
YAP1	PMID:32632093
ZCCHC14	
ZNF429	PMID:19207018
ZNF555	PMID:22678113
ZNF618	
ZSCAN20	PMID:26629533
ZSCAN25	PMID:31269327
