# Supplementary material for: Impaired Molecular Mechanisms Contributing to Chronic Pain in Patients with Hidradenitis Suppurativa: Exploring Potential Biomarkers and Therapeutic Targets
Source: Int J Mol Sci. 2025 Jan 25;26(3):1039. doi: 10.3390/ijms26031039 (PMC11817842; doi:10.3390/ijms26031039)
Supplement: Supplementary file 1 [file ijms-26-01039-s001.zip › Supplementary Table S3.pdf]

Supplementary Table S3. Pain-Related Genes in HS and their biological roles.

Genes	Gene involved	Gene involved	Gene involved	Gene involved	Gene involved	Gene involved
BDNF	Anorexia;Appetite regulation ;Bulimia nervosa' Lithium; selenium; Impulsivity	Stroke associated	Addiction	Immunomodulator; Autophagy	Obesity linked	Suicide linked
DRD2	Opioid Receptors	Bulimia nervosa	Addiction	Impulsivity/ Compulsivity	Obesity linked	Suicide linked
CNR2			Addiction		Obesity linked	
GABBR1	Opioid Receptors		Addiction			
RUNX1	Asthma		Asthma	Immunomodulator		
SOD2	Cadmium; Iron; Manganese	Telomelength	Circadian	Immunomodulator	Obesity linked	Suicide linked
IFNG			Circadian	Immunomodulator		Suicide linked
IL1R2			Circadian	Immunomodulator		
DNMT1	TET Enzymers	Telomelength	Circadian / Ultradian	Immunomodulator	Obesity linked	Suicide linked
COL4A1	Wound healing	Stroke associated	Extracellular matrix			
TGFA	Wound healing	Depression	Glucose metabolism	Immunomodulator		
STAT6			Glucose metabolism	Immunomodulator		
CACNA1C	Sudden Cardiac Death	Calcium	Hypertension	Glucose metabolism	Circadian	Suicide linked
AHR	Arylhydrocarbon receptor	Telomelength	Immunomodulator	Glucose metabolism		
PRKCA	Pkcα modulates TRPV1; Hypoxia related	Calcium	Impulsivity	Immunomodulator; Autophagy	Circadian / Ultradian	Suicide linked
RORA	Stress and Trauma genes	Hypoxia related ; Hormones	Impulsivity	Circadian		
NRG1		Neural plasticity Genes	Impulsivity	Immunomodulator		
TPH2	Serotonin	Calcium	Impulsivity			Suicide linked

HTR2A	Stress and Trauma; Serotonin /smoking	Calcium	Impulsivity/ Compulsivity		Obesity linked	Suicide linked
CP	Hypoxia related		Iron	Glucose metabolism		
CLIC1			Telomelength	Chloride ion channel		
CSNK1D			Telomelength	Circadian		
NOS3	Stroke associated	Calcium	Telomelength	Hypertension	Obesity linked	Suicide linked
SMAD3		Stroke associated	Telomelength	Immunomodulator		Suicide linked
ABCC2			Telomelength	Transporters		
ABCC4			Telomelength	Transporters		
SMG6	Wound healing		Telomelength	Ultradian		
TEAD1	Wound healing		Telomelength	Ultradian		
FOXP2			Telomelength		Obesity linked	
PLCB1		Calcium	Telomelength			Suicide linked
HIC1			Telomelength			
POLE			Telomelength			
IKBKAP			Telomelength			
VGLL4			Telomelength			
SIGMAR1			Telomelength			
MTA1			Telomelength			
POLR1C			Telomelength			
UTRN			Telomelength			
PRX			Telomelength			
PIK3C2G			Telomelength			
NRIP1			Telomelength			
CLCN6			Ultradian	Chloride ion channel		
PHACTR1	Wound healing		Ultradian			
MMP2	Glucose metabolism	Metalloproteinases	Zinc	Immunomodulator	Obesity linked	

SLC39A9			Zinc	Transporters		
MMP3	Hypoxia related;		Zinc			
SLC39A8			Zinc / Manganese	Transporters	Obesity linked	
CBS	Appetite regulation	Stroke associated	Zinc / Selenium	Circadian	Compulsivity	
TGFB1	Woundhealing	Depression		Immunomodulator; Circadian	Obesity linked	Suicide linked
POMC	Appetite regulation; endocrine gland-related	Depression		Immunomodulator; Glucose metabolism	Obesity linked	Suicide linked
SLC6A3		Addiction		Impulsivity; Compulsivity; Transporters	Obesity linked	Suicide linked
ESR2	Anorexia/ hormones	Telomelength		Autophagy	Obesity linked	Suicide linked
LMX1B				Autophagy	Obesity linked	
ULK4				Autophagy		
CLIC5				Chloride ion channel		
DOCK4	Apocrine gland genes			Circadian	Apocrineglands	
CYP19A1	Endocrine gland Genes	Impulsivity Glucose metabolism		Cytochrome	Obesity linked	Suicide linked
CYP1A2	Arylhydrocarbon receptor			Cytochrome		
KLF11				Glucose metabolism	Circadian	
EXT2				Glucose metabolism	Circadian	
GNAS	Thyrodism;<br>Sudden Cardiac Death	Calcium		Glucose metabolism	Obesity linked	Suicide linked
ENPP1		Stroke associated		Glucose metabolism	Obesity linked	
MC4R	Anorexia / Appetite regulation			Glucose metabolism	Obesity linked	
GLIS3	Thyrodism			Glucose metabolism	Obesity linked	
IRS1				Glucose metabolism	Obesity linked	
NOTCH3		Stroke associated		Glucose metabolism		Suicide linked
CACNA1H	Calcium			Glucose metabolism		

MC2R	Endocrine gland Genes			Glucose metabolism		
GPD2				Glucose metabolism		
CHRM2		Calcium		Hypertension	Addiction	
CACNB2	Sudden Cardiac Death	Calcium		Hypertension	Circadian	Suicide linked
PDE10A				Hypertension		
TNC	Woundhealing	Extracellular matrix		Immunomodulator	Apocrineglands	
TGFBR2		Stroke associated		Immunomodulator	Apocrineglands	
LTA				Immunomodulator	Obesity linked	
TNFRSF1B				Immunomodulator	Obesity linked	
CX3CR1		Hypoxia related		Immunomodulator		Suicide linked
NCAM1	Woundhealing			Immunomodulator		Suicide linked
EREG		Calcium		Immunomodulator		
FGF2	Woundhealing			Immunomodulator		
DPP4				Immunomodulator		
IL18R1				Immunomodulator		
IL12B				Immunomodulator		
IL19				Immunomodulator		
NOTCH4		Hypoxia related		Impulsivity		Suicide linked
PRKG1	Stroke associated			Impulsivity		
MAPK10				Impulsivity		
CTNNA2				Impulsivity		
KCNMA1				Potassium channels	Obesity linked	
KCNQ5				Potassium channels		Suicide linked
KCND3	Sudden Cardiac Death			Potassium channels		
KCND2				Potassium channels		

KCNB2				Potassium channels		
KCNAB3				Potassium channels		
SCN8A				Sodium Channel		Suicide linked
SCN5A		Stroke associated		Sodium Channel		
SCN11A				Sodium Channel		
SCNN1A				Sodium channel		
SCN3A				Sodium Channel		
CAMK2A	Calcium			Telomelength		
PLCB3	Calcium			Telomelength		
ABCG1				Transporters	Circadian	Suicide linked
SLC24A3				Transporters		Suicide linked
SLC44A2		Stroke associated		Transporters		
SLC12A5	Cobalt			Transporters		
SLC10A7				Transporters		
SLC24A4				Transporters		
SLC25A3				Transporters		
TRPV1	Heat and pain from spicy foods.			TRP channels		
TRPV2				TRP channels		
TRPM8				TRP channels		
TRPM2				TRP channels		
TSC2	Woundhealing	Stroke associated		Ultradian		Suicide linked
MAML2	Woundhealing			Ultradian		Suicide linked
NF1	Woundhealing	Stroke associated		Ultradian		
GRM1	Opioid Receptors	Calcium			Addiction	Suicide linked
SYNE1	Apocrine gland genes	Telomelength			Apocrineglands	

TUG1	Apocrine gland genes					Apocrineglands	
SPON1		Depression				Circadian	Suicide linked
P2RY12		Stroke associated				Circadian	Suicide linked
ADAMTSL4		Disintegrin				Circadian	
RNF213		Stroke associated				Circadian	
LRIG3						Circadian	
ARNTL						Circadian	
RAMP1						Circadian	
GRIN2B	Opioid Receptors	Neural plasticity Genes				Compulsivity	Suicide linked
FTO	Anorexia					Obesity linked	Suicide linked
FAAH		Addiction				Obesity linked	Suicide linked
CHRM3		Calcium				Obesity linked	
HCRTR1		Calcium				Obesity linked	
PON1		Stroke associated				Obesity linked	
MYT1L						Obesity linked	
SDK1						Obesity linked	
CACNA2D3	Iron	Calcium					Suicide linked
CACNG2	Lithium	Calcium					Suicide linked
GRIN2A	Opioid Receptors; Neural plasticity	Calcium					Suicide linked
TACR1		Calcium					Suicide linked
ADRA1A		Calcium					Suicide linked
TG	Thyrodism	Endocrine gland Genes					Suicide linked

HTRA1		Stroke associated				Suicide linked
OPRD1	Opioid Receptors	Addiction				Suicide linked
BMP6		Iron				Suicide linked
GABRB3	Opioid Receptors	Telomelength				Suicide linked
DLG2		Telomelength				Suicide linked
PRDM16		Telomelength				Suicide linked
DCC		Impulsivity				Suicide linked
FGF3	Woundhealing					Suicide linked
EXD3						Suicide linked
ADARB2						Suicide linked
NRP1						Suicide linked
SNX8						Suicide linked
EFNB2						Suicide linked
WWP2						Suicide linked
FAM134B						Suicide linked
RAPH1						Suicide linked
GABRB1	Opioid Receptors	Essentialtremor				
MAP2K1		Stroke associated				
MIR132	Woundhealing	Zinc				

SNAP25	Calcium					
AVPR1A	Calcium					
ADAMTSL1	Disintegrin					
PDGFC	Woundhealing					
FGF6	Woundhealing					
ESRRB	Hormones					
CASP9	Hypoxia related					
CHST3	Hypoxia related					
OPRK1	Opioid Receptors					
GRM7	Opioid Receptors					
HMOX2						
OXR1						
RNF144B						
PARD6G						
CUX1						
FNDC3B						
LPP						
ADORA3						
C7orf50						
MIR499						
ANKH						
GRK5						
AJAP1						
TBC1D7						
NCOR2						
LRP1						
MPPED2						
FRMD4A						

JAKMIP3						
HLA-DPA1						
DCDC1						
CRADD						
ACAN						
SHANK3						
KNDC1						
APOL3						
EHMT2						
TIPIN						
KIF1A						
PTN						
FAF1						
SPOCK2						
SYN3						
ATXN1						
WSCD1						
TSEN15						
BBX						
RSU1						
CCDC81						
KDM2A						
MTHFD1						
MAML3						
MN1						
KIAA0040						
TAOK3						
PCSK6						

SPTLC2						
GFRA2						
C8orf34						
SVEP1						
NPSR1						
LPAR5						
NLGN2						
ATP2C2						
ATL1						
SYT16						
ZSCAN20						
MRC2						
DGKI						
FAM101A						
MLLT10						
ASTN1						
TSSC1						
NMRAL1						
TACC3						
PENK						
WSCD2						
TLL2						
HDC						
VEPH1						
C12orf60						
DDO						
RAG1						
GALR1						

FAM183B						
RGS12						
MRVI1						
C17orf67						
ICA1						
CAPN1						
UBAP2						
RGS9						
MAP2K6						
C7orf10						
BEGAIN						
