# Supplementary material for: Impaired Molecular Mechanisms Contributing to Chronic Pain in Patients with Hidradenitis Suppurativa: Exploring Potential Biomarkers and Therapeutic Targets
Source: Int J Mol Sci. 2025 Jan 25;26(3):1039. doi: 10.3390/ijms26031039 (PMC11817842; doi:10.3390/ijms26031039)
Supplement: Supplementary file 1 [file ijms-26-01039-s001.zip › Supplementary Table S4.pdf]

Supplementary Table S4. Comprehensive List of Gene Ontology (GO) Terms for Biological Processes (BP)

ID	Description	Gene ID	p-value	p.adjust
GO:0042391	Regulation of membrane potential	CACNB2/KCNMA1/BDNF/DRD2/GRIN2B/CACNA1C/SCN8A/GABRB3/GRIN2A/CACNA1H/WWP2/NLGN2/TRPV1/KCND3/OPRD1/CNR2/CACNG2/SHANK3/SCN3A/SCN11A/SCN5A/GABRB1/SLC39A8/CLIC1/GABBR1/GRM1/KCND2/DGKI/PTN/ADRA1A	3.54156E-14	1.36102E-10
GO:0034765	Regulation of ion transmembrane transport	CACNB2/KCNMA1/CAPN1/DLG2/DRD2/GRIN2B/CACNA1C/SCN8A/IFNG/GRIN2A/CACNA1H/WWP2/KCNAB3/NLGN2/RGS9/KCND3/CHRM3/CACNG2/SHANK3/SCN3A/CACNA2D3/SCN11A/SCN5A/CLIC5/CLIC1/KCNQ5/UTRN/NPSR1/KCND2/KCNB2	4.62612E-13	8.88908E-10
GO:0072511	Divalent inorganic cation transport	CACNB2/ABCC2/DRD2/GRIN2B/CACNA1C/HTR2A/SLC39A9/SLC24A4/GRIN2A/CACNA1H/ATP2C2/TRPV2/TRPV1/RGS9/OPRD1/SLC24A3/TRPM2/CACNG2/TRPM8/RAMP1/CACNA2D3/P2RY12/SLC39A8/FGF2/CAMK2A/NPSR1/NOS3/ADRA1A	8.90267E-12	1.14043E-08
GO:0070838	Divalent metal ion transport	CACNB2/DRD2/GRIN2B/CACNA1C/HTR2A/SLC39A9/SLC24A4/GRIN2A/CACNA1H/ATP2C2/TRPV2/TRPV1/RGS9/OPRD1/SLC24A3/TRPM2/CACNG2/TRPM8/RAMP1/CACNA2D3/P2RY12/SLC39A8/FGF2/CAMK2A/NPSR1/NOS3/ADRA1A	3.52379E-11	3.38548E-08
GO:0006816	Calcium ion transport	CACNB2/DRD2/GRIN2B/CACNA1C/HTR2A/SLC24A4/GRIN2A/CACNA1H/ATP2C2/TRPV2/TRPV1/RGS9/OPRD1/SLC24A3/TRPM2/CACNG2/TRPM8/RAMP1/CACNA2D3/P2RY12/FGF2/CAMK2A/NPSR1/NOS3/ADRA1A	1.20401E-10	9.254E-08
GO:0072503	Cellular divalent inorganic cation homeostasis	CACNB2/DRD2/GRIN2B/CACNA1C/LRP1/AVPR1A/HTR2A/SLC24A4/SMAD3/GRIN2A/ATP2C2/TRPV2/TRPV1/GALR1/HCRTR1/SLC24A3/TRPM2/TACR1/TRPM8/CX3CR1/SLC39A8/FGF2/SLC10A7/GRM1/NPSR1/ADRA1A	4.845E-10	2.74833E-07
GO:0006874	Cellular calcium ion homeostasis	CACNB2/DRD2/GRIN2B/CACNA1C/LRP1/AVPR1A/HTR2A/SLC24A4/SMAD3/GRIN2A/ATP2C2/TRPV2/TRPV1/GALR1/HCRTR1/SLC24A3/TRPM2/TACR1/TRPM8/CX3CR1/FGF2/SLC10A7/GRM1/NPSR1/ADRA1A	5.00606E-10	2.74833E-07
GO:0019233	Sensory perception of pain	DLG2/HTR2A/GRIN2A/NLGN2/TRPV1/OPRD1/CNR2/SCN11A/IL12B/GRM1/KCND2/OPRK1/PENK	6.41864E-10	3.08335E-07

GO:0055074	Calcium ion homeostasis	CACNB2/DRD2/GRIN2B/CACNA1C/LRP1/AVPR1A/HTR2A/SLC24A4/SMAD3/GRIN2A/ATP2C2/TRPV2/TRPV1/GALR1/HCRTR1/SLC24A3/TRPM2/TACR1/TRPM8/CX3CR1/FGF2/SLC10A7/GRM1/NPSR1/ADRA1A	8.56482E-10	3.65718E-07
GO:0070588	Calcium ion transmembrane transport	CACNB2/DRD2/GRIN2B/CACNA1C/HTR2A/SLC24A4/GRIN2A/CACNA1H/ATP2C2/TRPV2/TRPV1/RGS9/SLC24A3/TRPM2/CACNG2/TRPM8/CACNA2D3/P2RY12/FGF2/NPSR1	2.10455E-09	7.82233E-07
GO:0050804	Modulation of chemical synaptic transmission	CACNB2/BDNF/DRD2/GRIN2B/HTR2A/BEGAIN/GRIN2A/NLGN2/NF1/DCC/CNR2/SNAP25/PLCB1/CACNG2/SYN3/SHANK3/CX3CR1/GRM7/CAMK2A/GRM1/CHRM2/DGKI/PTN/ADRA1A	2.33896E-09	7.82233E-07
GO:0099177	Regulation of trans-synaptic signaling	CACNB2/BDNF/DRD2/GRIN2B/HTR2A/BEGAIN/GRIN2A/NLGN2/NF1/DCC/CNR2/SNAP25/PLCB1/CACNG2/SYN3/SHANK3/CX3CR1/GRM7/CAMK2A/GRM1/CHRM2/DGKI/PTN/ADRA1A	2.44257E-09	7.82233E-07
GO:0007215	Glutamate receptor signaling pathway	CAPN1/DLG2/GRIN2B/IFNG/GRIN2A/NLGN2/RGS9/PLCB1/CACNG2/SHANK3/GRM7/GRM1	4.2923E-09	1.26887E-06
GO:0007187	G protein-coupled receptor signaling pathway, coupled to cyclic nucleotide second messenger	GRK5/DRD2/HTR2A/PRKCA/MC2R/MC4R/GALR1/OPRD1/CNR2/CHRM3/GNAS/RAMP1/GRM7/P2RY12/GABBR1/CHRM2/ADRA1A/OPRK1	4.97717E-09	1.32595E-06
GO:0007188	Adenylate cyclase-modulating G protein-coupled receptor signaling pathway	GRK5/DRD2/PRKCA/MC2R/MC4R/GALR1/OPRD1/CNR2/CHRM3/GNAS/RAMP1/GRM7/P2RY12/GABBR1/CHRM2/ADRA1A/OPRK1	5.17545E-09	1.32595E-06
GO:0051480	Regulation of cytosolic calcium ion concentration	CACNB2/DRD2/GRIN2B/CACNA1C/LRP1/AVPR1A/HTR2A/SMAD3/GRIN2A/TRPV2/TRPV1/GALR1/HCRTR1/TRPM2/TACR1/CX3CR1/FGF2/GRM1/NPSR1/ADRA1A	2.06636E-08	4.96313E-06
GO:0007626	Locomotory behavior	DRD2/MTA1/NLGN2/OPRD1/ASTN1/SNAP25/DPP4/ANKH/SLC6A3/GRM1/KCND2/NRG1/OPRK1/PENK/OXR1	2.56084E-08	5.78901E-06
GO:0060078	Regulation of postsynaptic membrane potential	BDNF/DRD2/GRIN2B/GABRB3/GRIN2A/NLGN2/TRPV1/SHANK3/GABRB1/GABBR1/GRM1/KCND2/DGKI	2.80663E-08	5.99216E-06
GO:0001659	Temperature homeostasis	ARNTL/DRD2/STAT6/HTR2A/FTO/TRPV2/TRPV1/MAP2K6/PRDM16/GNAS/TRPM2/IL18R1/TRPM8/PDGFC	3.54221E-08	6.80636E-06
GO:0010469	Regulation of signaling receptor activity	NRP1/CAPN1/DLG2/GRIN2B/IFNG/ESR2/BEGAIN/GRIN2A/NLGN2/RGS9/CACNG2/SHANK3/TGFA/EREG	3.54221E-08	6.80636E-06
GO:1903522	Regulation of blood circulation	CACNB2/DRD2/CACNA1C/AVPR1A/HTR2A/CACNA1H/TRPV1/ADORA3/KCND3/CHRM3/CACNG2/CACNA2D3/SCN5A/DOCK4/KCND2/CHRM2/NOS3/ADRA1A	4.45709E-08	8.1312E-06

GO:0099601	Regulation of neurotransmitter receptor activity	CAPN1/DLG2/GRIN2B/IFNG/BEGAIN/GRIN2A/NLGN2/RGS9/CACNG2/SHANK3	4.65486E-08	8.1312E-06
GO:0048511	Rhythmic process	BDNF/ARNTL/KDM2A/DRD2/TPH2/NCOR2/MTA1/RORA/MAP2K6/CSNK1D/NRIP1/GABRB1/EREG/MAPK10/AHR/KCND2/PTN/NOS3	4.92606E-08	8.23081E-06
GO:0019932	Second-messenger-mediated signaling	PRKG1/DRD2/GRIN2B/CACNA1C/AVPR1A/GRIN2A/PRKCA/MC2R/MC4R/GALR1/CNR2/CHRM3/GNAS/TRPM2/RAMP1/CX3CR1/P2RY12/PDE10A/AHR/NOS3/NRG1/ADRA1A	5.7412E-08	9.19309E-06
GO:0003012	Muscle system process	CACNB2/PRKG1/KCNMA1/DRD2/CACNA1C/HTR2A/SMAD3/CACNA1H/TRPV1/PRKCA/MAP2K6/KCND3/TNFRSF1B/CHRM3/TACR1/SCN5A/UTRN/DOCK4/CHRM2/NOS3/ADRA1A/KCNB2	8.71124E-08	1.29229E-05
GO:1900449	Regulation of glutamate receptor signaling pathway	CAPN1/DLG2/GRIN2B/IFNG/GRIN2A/NLGN2/RGS9/CACNG2/SHANK3	8.74305E-08	1.29229E-05
GO:0006939	Smooth muscle contraction	PRKG1/KCNMA1/DRD2/HTR2A/TRPV1/CHRM3/TACR1/DOCK4/CHRM2/ADRA1A/KCNB2	1.06776E-07	1.46645E-05
GO:0050808	Synapse organization	NRP1/CACNB2/SPOCK2/BDNF/DLG2/DRD2/GRIN2B/EFNB2/COL4A1/GABRB3/NLGN2/CACNG2/SHANK3/CTNNA2/KIF1A/CX3CR1/UTRN/SDK1/PTN/NRG1/TNC	1.06845E-07	1.46645E-05
GO:0007204	Positive regulation of cytosolic calcium ion concentration	CACNB2/DRD2/GRIN2B/CACNA1C/LRP1/AVPR1A/HTR2A/GRIN2A/TRPV2/TRPV1/GALR1/TRPM2/TACR1/CX3CR1/FGF2/GRM1/NPSR1/ADRA1A	1.11661E-07	1.4797E-05
GO:0009914	Hormone transport	ABCC2/ARNTL/DRD2/CACNA1C/LRP1/IFNG/CYP19A1/NLGN2/PRKCA/MC4R/GALR1/SNAP25/GNAS/POMC/DPP4/IRS1/BMP6/ICA1	1.16967E-07	1.49835E-05
GO:0007611	Learning or memory	RAG1/BDNF/DRD2/GRIN2B/HTR2A/GRIN2A/NF1/SNAP25/PLCB1/SLC12A5/SHANK3/CX3CR1/ATXN1/FOXP2/DGKI/PTN	1.58372E-07	1.96331E-05
GO:0032228	Regulation of synaptic transmission, gabaergic	BDNF/DRD2/NLGN2/NF1/CNR2/SYN3/ADRA1A	2.00632E-07	2.40946E-05
GO:0046883	Regulation of hormone secretion	ARNTL/DRD2/CACNA1C/LRP1/IFNG/CYP19A1/NLGN2/PRKCA/GALR1/SNAP25/GNAS/POMC/DPP4/IRS1/BMP6/ICA1	2.27298E-07	2.64698E-05
GO:0086010	Membrane depolarization during action potential	CACNB2/CACNA1C/SCN8A/CACNA1H/SCN3A/SCN11A/SCN5A	3.00789E-07	3.30266E-05
GO:2000273	Positive regulation of signaling receptor activity	NRP1/IFNG/RGS9/CACNG2/SHANK3/TGFA/EREG	3.00789E-07	3.30266E-05
GO:0006813	Potassium ion transport	KCNMA1/DRD2/HTR2A/SLC24A4/WWP2/KCNAB3/KCND3/SLC24A3/SNAP25/SLC12A5/P2RY12/KCNQ5/KCND2/NOS3/KCNB2	3.55751E-07	3.79764E-05
GO:0046879	Hormone secretion	ARNTL/DRD2/CACNA1C/LRP1/IFNG/CYP19A1/NLGN2/PRKCA/MC4R/GALR1/SNAP25/GNAS/POMC/DPP4/IRS1/BMP6/ICA1	3.99106E-07	4.14531E-05

GO:0030198	Extracellular matrix organization	SPOCK2/TLL2/HTRA1/CAPN1/MMP3/LRP1/COL4A1/SMAD3/ACAN/MMP2/NF1/TGFB1/TNFRSF1B/ADAMTSL4/DPP4/SLC39A8/FGF2/ADAMTSL1/TNC	5.01365E-07	5.07039E-05
GO:0043062	Extracellular structure organization	SPOCK2/TLL2/HTRA1/CAPN1/MMP3/LRP1/COL4A1/SMAD3/ACAN/MMP2/NF1/TGFB1/TNFRSF1B/ADAMTSL4/DPP4/SLC39A8/FGF2/ADAMTSL1/TNC	5.20917E-07	5.13304E-05
GO:0060047	Heart contraction	CACNB2/DRD2/CACNA1C/AVPR1A/CACNA1H/TRPV1/MAP2K6/ADORA3/KCND3/CACNG2/CACNA2D3/SCN5A/KCND2/CHRM2/NOS3/ADRA1A	5.99671E-07	5.76134E-05
GO:0006936	Muscle contraction	CACNB2/PRKG1/KCNMA1/DRD2/CACNA1C/HTR2A/CACNA1H/TRPV1/MAP2K6/KCND3/CHRM3/TACR1/SCN5A/UTRN/DOCK4/CHRM2/ADRA1A/KCNB2	6.27023E-07	5.8772E-05
GO:0007200	Phospholipase C-activating G protein-coupled receptor signaling pathway	DRD2/HTR2A/OPRD1/TACR1/CX3CR1/P2RY12/GRM1/CHRM2/ADRA1A/OPRK1	6.44314E-07	5.89548E-05
GO:0008016	Regulation of heart contraction	CACNB2/DRD2/CACNA1C/AVPR1A/CACNA1H/TRPV1/ADORA3/KCND3/CACNG2/CACNA2D3/SCN5A/KCND2/CHRM2/NOS3/ADRA1A	7.2773E-07	6.35606E-05
GO:0090257	Regulation of muscle system process	PRKG1/KCNMA1/CACNA1C/SMAD3/PRKCA/TNFRSF1B/CHRM3/TACR1/SCN5A/UTRN/DOCK4/CHRM2/NOS3/ADRA1A/KCNB2	7.2773E-07	6.35606E-05
GO:0003015	Heart process	CACNB2/DRD2/CACNA1C/AVPR1A/CACNA1H/TRPV1/MAP2K6/ADORA3/KCND3/CACNG2/CACNA2D3/SCN5A/KCND2/CHRM2/NOS3/ADRA1A	9.43792E-07	8.05998E-05
GO:0001508	Action potential	CACNB2/CACNA1C/SCN8A/CACNA1H/KCND3/CNR2/SCN3A/SCN11A/SCN5A/KCND2/ADRA1A	1.17003E-06	9.6113E-05
GO:0050890	Cognition	RAG1/BDNF/DRD2/GRIN2B/HTR2A/GRIN2A/NF1/SNAP25/PLCB1/SLC12A5/SHANK3/CX3CR1/ATXN1/FOXP2/DGKI/PTN	1.17547E-06	9.6113E-05
GO:0051781	Positive regulation of cell division	FGF3/DRD2/FGF6/TGFB1/TGFA/EREG/FGF2/PDGFC/PTN	1.27308E-06	0.000101926
GO:0006940	Regulation of smooth muscle contraction	PRKG1/KCNMA1/CHRM3/TACR1/DOCK4/CHRM2/ADRA1A/KCNB2	1.32159E-06	0.000103651
GO:0032355	Response to estradiol	ABCC2/NCOR2/ESR2/CYP19A1/CYP1A2/RGS9/TGFB1/CASP9/NRIP1/PTN/PENK	1.44792E-06	0.000111287
GO:0032922	Circadian regulation of gene expression	ARNTL/KDM2A/DRD2/MTA1/RORA/CSNK1D/NRIP1/AHR	1.48802E-06	0.000112126
GO:0050806	Positive regulation of synaptic transmission	CACNB2/DRD2/GRIN2B/GRIN2A/NLGN2/NF1/SNAP25/CACNG2/SHANK3/CX3CR1/PTN/ADRA1A	1.57547E-06	0.000116433

GO:0048660	Regulation of smooth muscle cell proliferation	PRKG1/IFNG/ABCC4/MMP2/NOTCH3/DNMT1/TGFBR2/EREG/FGF2/IL12B/SOD2/NOS3	1.67465E-06	0.000121428
GO:1904062	Regulation of cation transmembrane transport	CACNB2/CAPN1/DLG2/DRD2/GRIN2B/CACNA1C/IFNG/GRIN2A/WWP2/KCNAB3/NLGN2/RGS9/CACNG2/SHANK3/SCN5A/UTRN/NPSR1	1.71776E-06	0.000122247
GO:0030900	Forebrain development	NRP1/PRKG1/DRD2/LRP1/AVPR1A/NF1/NOTCH3/PLCB1/SHANK3/KIF1A/SCN5A/P2RY12/TACC3/SLC6A3/PHACTR1/FOXP2/PTN/NRG1	1.88928E-06	0.000124839
GO:0048659	Smooth muscle cell proliferation	PRKG1/IFNG/ABCC4/MMP2/NOTCH3/DNMT1/TGFBR2/EREG/FGF2/IL12B/SOD2/NOS3	1.88972E-06	0.000124839
GO:0006814	Sodium ion transport	DRD2/SCNN1A/SCN8A/SLC24A4/CACNA1H/SLC24A3/TRPM2/SCN3A/SCN11A/SCN5A/SLC10A7/SLC6A3/UTRN/NOS3	1.90164E-06	0.000124839
GO:0007612	Learning	RAG1/DRD2/GRIN2A/NF1/SNAP25/SLC12A5/SHANK3/ATXN1/FOXP2/DGKI/PTN	1.90782E-06	0.000124839
GO:0051932	Synaptic transmission, gabaergic	BDNF/DRD2/NLGN2/NF1/CNR2/SYN3/ADRA1A	1.91661E-06	0.000124839
GO:0060401	Cytosolic calcium ion transport	DRD2/GRIN2B/CACNA1C/HTR2A/GRIN2A/TRPV2/TRPV1/RGS9/TRPM2/FGF2/NPSR1/ADRA1A	2.25815E-06	0.000144635
GO:0016358	Dendrite development	NRP1/PRKG1/KNDC1/BDNF/DCC/SLC12A5/SHANK3/CTNNA2/KIF1A/CAMK2A/PHACTR1/SDK1/CUX1/PTN	2.53764E-06	0.000157817
GO:0007528	Neuromuscular junction development	CACNB2/COL4A1/CACNG2/SHANK3/UTRN/PTN/TNC	2.5461E-06	0.000157817
GO:0048732	Gland development	NRP1/RAG1/CAPN1/DRD2/STAT6/NCOR2/CYP19A1/MAP2K1/SMAD3/ATP2C2/NF1/TGFB1/TGFBR2/SLC6A3/NOTCH4/PTN/NRG1/TG/TNC	2.75384E-06	0.000167984
GO:0019226	Transmission of nerve impulse	SCN8A/AVPR1A/CACNA1H/CACNG2/SCN3A/SCN11A/SCN5A/KCND2	2.90978E-06	0.000172815
GO:0035249	Synaptic transmission, glutamatergic	DRD2/HTR2A/NLGN2/NF1/CACNG2/SHANK3/GRM7/GRM1/DGKI	2.92297E-06	0.000172815
GO:0051966	Regulation of synaptic transmission, glutamatergic	DRD2/HTR2A/NLGN2/CACNG2/SHANK3/GRM7/GRM1/DGKI	3.23346E-06	0.000188275
GO:0019228	Neuronal action potential	SCN8A/CACNA1H/SCN3A/SCN11A/SCN5A/KCND2	3.35048E-06	0.000192177
GO:0032409	Regulation of transporter activity	CACNB2/CAPN1/DLG2/DRD2/GRIN2B/IFNG/GRIN2A/WWP2/NLGN2/RGS9/CHRM3/CACNG2/SHANK3/UTRN/PON1	3.57564E-06	0.000202076
GO:1990845	Adaptive thermogenesis	ARNTL/STAT6/TRPV2/TRPV1/MAP2K6/MC4R/PRDM16/GNAS/IL18R1/TRPM8/PDGFC	3.65934E-06	0.000203809

GO:0060402	Calcium ion transport into cytosol	DRD2/GRIN2B/CACNA1C/HTR2A/GRIN2A/TRPV2/TRPV1/TRPM2/FGF2/NPSR1/ADRA1A	4.40539E-06	0.000240623
GO:0003018	Vascular process in circulatory system	PRKG1/KCNMA1/ABCC2/AVPR1A/HTR2A/TGFB1/CHRM3/BMP6/SOD2/DOCK4/NOS3/ADRA1A	4.44554E-06	0.000240623
GO:2000310	Regulation of NMDA receptor activity	CAPN1/DLG2/GRIN2B/IFNG/GRIN2A/RGS9	4.75331E-06	0.000253708
GO:0032412	Regulation of ion transmembrane transporter activity	CACNB2/CAPN1/DLG2/DRD2/GRIN2B/IFNG/GRIN2A/WWP2/NLGN2/RGS9/CHRM3/CACNG2/SHANK3/UTRN	5.73247E-06	0.000301779
GO:0150063	Visual system development	NRP1/BDNF/DRD2/CACNA1C/COL4A1/SMAD3/NF1/TUG1/GPD2/TGFBR2/P2RY12/FGF2/SLC6A3/BMP6/SDK1/FOXP2/PTN	7.07606E-06	0.000367477
GO:0022617	Extracellular matrix disassembly	TLL2/HTRA1/CAPN1/MMP3/LRP1/MMP2/TGFB1/DPP4	7.79393E-06	0.000399361
GO:0050708	Regulation of protein secretion	FRMD4A/ARNTL/DRD2/CACNA1C/LRP1/IFNG/NLGN2/PRKCA/TGFB1/SNAP25/ABCG1/DPP4/IRS1/IL12B/BMP6/ICA1	8.38122E-06	0.000423803
GO:0048880	Sensory system development	NRP1/BDNF/DRD2/CACNA1C/COL4A1/SMAD3/NF1/TUG1/GPD2/TGFBR2/P2RY12/FGF2/SLC6A3/BMP6/SDK1/FOXP2/PTN	8.65052E-06	0.00043174
GO:0140353	Lipid export from cell	ABCC4/CYP19A1/MAP2K6/GALR1/POMC/BMP6	8.98853E-06	0.000442858
GO:0022898	Regulation of transmembrane transporter activity	CACNB2/CAPN1/DLG2/DRD2/GRIN2B/IFNG/GRIN2A/WWP2/NLGN2/RGS9/CHRM3/CACNG2/SHANK3/UTRN	9.11637E-06	0.000443471
GO:0010959	Regulation of metal ion transport	CACNB2/DRD2/CACNA1C/IFNG/HTR2A/WWP2/ATP2C2/TRPV2/KCNAB3/RGS9/OPRD1/SCN5A/P2RY12/CAMK2A/UTRN/NPSR1/NOS3	9.24117E-06	0.000443923
GO:0051302	Regulation of cell division	DCDC1/FGF3/DRD2/FGF6/ESRRB/TGFB1/TGFA/EREG/FGF2/PDGFC/PTN	9.39317E-06	0.000445654
GO:0097553	Calcium ion transmembrane import into cytosol	DRD2/GRIN2B/CACNA1C/HTR2A/GRIN2A/TRPV2/TRPV1/TRPM2/FGF2/NPSR1	9.61757E-06	0.000450736
GO:0015698	Inorganic anion transport	SLC25A3/GABRB3/CLCN6/SLC12A5/GABRB1/SLC39A8/ANKH/CLIC5/CLIC1/ENPP1/TG	9.9273E-06	0.000459646
GO:0035637	Multicellular organismal signaling	CACNB2/CACNA1C/SCN8A/AVPR1A/CACNA1H/KCND3/CACNG2/SCN3A/CACNA2D3/SCN11A/SCN5A/KCND2	1.01451E-05	0.00046414
GO:0099565	Chemical synaptic transmission, postsynaptic	BDNF/DRD2/GRIN2B/GABRB3/GRIN2A/NLGN2/TRPV1/SHANK3/DGKI	1.0442E-05	0.000472102
GO:0035725	Sodium ion transmembrane transport	SCNN1A/SCN8A/SLC24A4/CACNA1H/SLC24A3/TRPM2/SCN3A/SCN11A/SCN5A/SLC6A3/UTRN	1.10753E-05	0.000491287

GO:0007193	Adenylate cyclase-inhibiting G protein-coupled receptor signaling pathway	DRD2/OPRD1/CHRM3/GRM7/P2RY12/GABBR1/CHRM2/OPRK1	1.1122E-05	0.000491287
GO:0033002	Muscle cell proliferation	PRKG1/IFNG/ABCC4/MMP2/NOTCH3/DNMT1/TGFBR2/VGLL4/EREG/FGF2/IL12B/SOD2/NOS3	1.13445E-05	0.000495421
GO:1901214	Regulation of neuron death	NRP1/BDNF/GRIN2B/LRP1/IFNG/EFNB2/GABRB3/NF1/DCC/CASP9/TNFRSF1B/CX3CR1/SOD2/OXR1/SIGMAR1	1.16347E-05	0.000502382
GO:0060291	Long-term synaptic potentiation	DRD2/GRIN2B/GRIN2A/NF1/SNAP25/SHANK3/CX3CR1/PTN	1.21194E-05	0.000517498
GO:0043270	Positive regulation of ion transport	CACNB2/DRD2/AVPR1A/IFNG/ATP2C2/TRPV2/RGS9/MAP2K6/CACNG2/SHANK3/SCN5A/P2RY12/CAMK2A/NPSR1	1.25876E-05	0.000531584
GO:0051897	Positive regulation of protein kinase B signaling	FGF3/FGF6/MIR132/TGFB1/TGFA/IRS1/CX3CR1/P2RY12/EREG/FGF2/NRG1	1.3722E-05	0.000573193
GO:0030073	Insulin secretion	ARNTL/DRD2/CACNA1C/LRP1/IFNG/NLGN2/PRKCA/MC4R/SNAP25/DPP4/IRS1/ICA1	1.4199E-05	0.000586722
GO:0050673	Epithelial cell proliferation	NRP1/HTRA1/STAT6/EFNB2/MAP2K1/SMAD3/MIR132/NF1/PRKCA/TGFB1/TGFA/SCN5A/EREG/FGF2/IL12B/BMP6/FOXP2/PTN	1.43513E-05	0.000586722
GO:0001503	Ossification	EXT2/SMAD3/MMP2/NF1/MRC2/TGFB1/SLC24A3/GNAS/RUNX1/MN1/ANKH/CLIC1/BMP6/ENPP1/PTN/PENK/TNC	1.54278E-05	0.000624097
GO:0050796	Regulation of insulin secretion	ARNTL/DRD2/CACNA1C/LRP1/IFNG/NLGN2/PRKCA/SNAP25/DPP4/IRS1/ICA1	1.60521E-05	0.000642587
GO:0051896	Regulation of protein kinase B signaling	FGF3/DRD2/FGF6/TSC2/MIR132/TGFB1/TGFA/IRS1/CX3CR1/P2RY12/EREG/FGF2/NRG1	1.66722E-05	0.000660527
GO:0051899	Membrane depolarization	CACNB2/CACNA1C/SCN8A/CACNA1H/CACNG2/SCN3A/SCN11A/SCN5A	1.68964E-05	0.00066258
GO:0007623	Circadian rhythm	BDNF/ARNTL/KDM2A/DRD2/TPH2/MTA1/RORA/CSNK1D/NRIP1/MAPK10/AHR/KCND2	1.79016E-05	0.000687957
GO:0071805	Potassium ion transmembrane transport	KCNMA1/SLC24A4/WWP2/KCNAB3/KCND3/SLC24A3/SNAP25/SLC12A5/P2RY12/KCNQ5/KCND2/KCNB2	1.79016E-05	0.000687957
GO:0014047	Glutamate secretion	BDNF/AVPR1A/TRPV1/NF1/SNAP25/GRM7	1.8116E-05	0.000689305
GO:0007613	Memory	BDNF/DRD2/HTR2A/GRIN2A/PLCB1/SHANK3/CX3CR1/ATXN1/PTN	1.9504E-05	0.000734572
GO:2001257	Regulation of cation channel activity	CACNB2/CAPN1/DLG2/DRD2/GRIN2B/IFNG/GRIN2A/NLGN2/RGS9/CACNG2/SHANK3	1.9688E-05	0.000734572

GO:0010863	Positive regulation of phospholipase C activity	BDNF/AVPR1A/HTR2A/P2RY12/FGF2/ADRA1A	2.062E-05	0.000761948
GO:0016202	Regulation of striated muscle tissue development	TLL2/ARNTL/FGF3/EFNB2/SMAD3/TGFB1/TGFBR2/VGLL4/FGF2/NRG1	2.09179E-05	0.000765596
GO:0001964	Startle response	DRD2/GRIN2A/CTNNA2/SLC6A3/PENK	2.14517E-05	0.000777724
GO:0002791	Regulation of peptide secretion	FRMD4A/ARNTL/DRD2/CACNA1C/LRP1/IFNG/NLGN2/PRKCA/TGFB1/SNAP25/ABCG1/DPP4/IRS1/IL12B/BMP6/ICA1	2.22705E-05	0.000799865
GO:0042698	Ovulation cycle	NCOR2/MAP2K6/NRIP1/GABRB1/EREG/PTN/NOS3	2.25408E-05	0.000802076
GO:0051047	Positive regulation of secretion	CACNB2/FRMD4A/DRD2/LRP1/AVPR1A/IFNG/CYP19A1/CACNA1H/NLGN2/TRPV1/MAP2K6/GALR1/TGFB1/ABCG1/BMP6	2.28403E-05	0.000805277
GO:0019935	Cyclic-nucleotide-mediated signaling	PRKG1/DRD2/PRKCA/MC2R/MC4R/GALR1/CNR2/GNAS/RAMP1/PDE10A/AHR/ADRA1A	2.34345E-05	0.000818716
GO:0001654	Eye development	NRP1/BDNF/DRD2/CACNA1C/COL4A1/SMAD3/NF1/TUG1/GPD2/TGFBR2/FGF2/SLC6A3/BMP6/SDK1/FOXP2/PTN	2.44979E-05	0.000842904
GO:1901861	Regulation of muscle tissue development	TLL2/ARNTL/FGF3/EFNB2/SMAD3/TGFB1/TGFBR2/VGLL4/FGF2/NRG1	2.47423E-05	0.000842904
GO:0032370	Positive regulation of lipid transport	LRP1/CYP19A1/MAP2K6/GALR1/ABCG1/BMP6/PON1	2.47848E-05	0.000842904
GO:0048634	Regulation of muscle organ development	TLL2/ARNTL/FGF3/EFNB2/SMAD3/TGFB1/TGFBR2/VGLL4/FGF2/NRG1	2.61436E-05	0.000877057
GO:1900274	Regulation of phospholipase C activity	BDNF/AVPR1A/HTR2A/P2RY12/FGF2/ADRA1A	2.64638E-05	0.000877057
GO:0048167	Regulation of synaptic plasticity	BDNF/DRD2/GRIN2B/GRIN2A/NF1/SNAP25/SHANK3/CX3CR1/CAMK2A/DGKI/PTN	2.64737E-05	0.000877057
GO:0032102	Negative regulation of response to external stimulus	NRP1/PRKG1/HTRA1/DRD2/CYP19A1/RORA/SMAD3/CNR2/TNFRSF1B/AJAP1/CTNNA2/IL1R2/DPP4/FGF2/SLC6A3/IL12B/NOS3	2.90399E-05	0.00094865
GO:0006836	Neurotransmitter transport	CACNB2/DRD2/HTR2A/NF1/SNAP25/SYN3/SLC6A3/CAMK2A/ICA1/CHRM2/DGKI/ADRA1A	2.91285E-05	0.00094865
GO:0031279	Regulation of cyclase activity	DRD2/CACNA1C/NF1/GALR1/GRM7/NOS3	2.98477E-05	0.000963906
GO:0015850	Organic hydroxy compound transport	ABCC2/DRD2/LRP1/HTR2A/CYP19A1/GALR1/ABCG1/POMC/P2RY12/SLC10A7/SLC6A3/BMP6/PON1	3.04982E-05	0.000976704
GO:0050731	Positive regulation of peptidyl-tyrosine phosphorylation	NRP1/BDNF/IFNG/HTR2A/TGFB1/TGFA/EREG/IL12B/BMP6/NRG1/ADRA1A	3.20463E-05	0.001017801

GO:0010517	Regulation of phospholipase activity	BDNF/LRP1/AVPR1A/HTR2A/P2RY12/FGF2/ADRA1A	3.26472E-05	0.001028387
GO:0050678	Regulation of epithelial cell proliferation	NRP1/HTRA1/EFNB2/SMAD3/MIR132/NF1/PRKCA/TGFB1/TGFA/SCN5A/EREG/FGF2/IL12B/BMP6/FOXP2/PTN	3.44511E-05	0.001076385
GO:0010876	Lipid localization	ABCC2/DRD2/LRP1/ABCC4/CYP19A1/FTO/MAP2K6/GALR1/NRIP1/ABCG1/APOL3/POMC/SLC10A7/BMP6/ENPP1/PON1/SIGMAR1	3.55192E-05	0.001100808
GO:0060191	Regulation of lipase activity	BDNF/LRP1/AVPR1A/HTR2A/PCSK6/P2RY12/FGF2/ADRA1A	3.61488E-05	0.001109133
GO:0042493	Response to drug	ABCC2/BDNF/DRD2/HTR2A/ABCC4/CYP1A2/GRIN2A/MAP2K6/SLC12A5/GNAS/SCN11A/TGFBR2/SLC6A3/LTA/PTN/ADRA1A	3.66028E-05	0.001109133
GO:1900451	Positive regulation of glutamate receptor signaling pathway	IFNG/RGS9/CACNG2/SHANK3	3.67208E-05	0.001109133
GO:1903532	Positive regulation of secretion by cell	CACNB2/FRMD4A/DRD2/LRP1/AVPR1A/IFNG/CYP19A1/CACNA1H/NLGN2/MAP2K6/GALR1/TGFB1/ABCG1/BMP6	3.69422E-05	0.001109133
GO:0001764	Neuron migration	NRP1/PRKG1/DRD2/DCC/ASTN1/CTNNA2/ULK4/CAMK2A/PHACTR1/NRG1	3.7997E-05	0.001131958
GO:0019933	Camp-mediated signaling	DRD2/PRKCA/MC2R/MC4R/GALR1/CNR2/GNAS/RAMP1/PDE10A/AHR/ADRA1A	3.86034E-05	0.001141175
GO:0050805	Negative regulation of synaptic transmission	BDNF/DRD2/HTR2A/NF1/CNR2/SHANK3/DGKI	3.89476E-05	0.001142562
GO:0033555	Multicellular organismal response to stress	RAG1/BDNF/LPAR5/TRPV1/TACR1/DPP4/PENK	4.24531E-05	0.001235963
GO:0070997	Neuron death	NRP1/BDNF/GRIN2B/LRP1/IFNG/EFNB2/GABRB3/NF1/DCC/CASP9/TNFRSF1B/CX3CR1/SOD2/OXR1/SIGMAR1	4.40741E-05	0.001270623
GO:0043491	Protein kinase B signaling	FGF3/DRD2/FGF6/TSC2/MIR132/TGFB1/TGFA/IRS1/CX3CR1/P2RY12/EREG/FGF2/NRG1	4.45365E-05	0.001270623
GO:0002027	Regulation of heart rate	CACNB2/DRD2/CACNA1C/AVPR1A/TRPV1/KCND3/SCN5A/ADRA1A	4.46355E-05	0.001270623
GO:0014015	Positive regulation of gliogenesis	LRP1/TGFB1/TNFRSF1B/CX3CR1/P2RY12/LTA/PTN	4.62132E-05	0.001305863
GO:0007218	Neuropeptide signaling pathway	MC2R/GALR1/HCRTR1/OPRD1/POMC/NPSR1/OPRK1/PENK	5.11689E-05	0.001424942
GO:0046928	Regulation of neurotransmitter secretion	CACNB2/DRD2/HTR2A/NF1/CAMK2A/CHRM2/DGKI/ADRA1A	5.11689E-05	0.001424942
GO:0006809	Nitric oxide biosynthetic process	IFNG/RORA/SMAD3/TRPV1/MIR132/CX3CR1/NOS3	5.45541E-05	0.001508285

GO:0007202	Activation of phospholipase C activity	BDNF/AVPR1A/HTR2A/P2RY12/ADRA1A	5.7178E-05	0.001558404
GO:1902692	Regulation of neuroblast proliferation	BDNF/DRD2/NF1/CX3CR1/PTN	5.7178E-05	0.001558404
GO:0006937	Regulation of muscle contraction	PRKG1/KCNMA1/CACNA1C/CHRM3/TACR1/SCN5A/DOCK4/CHRM2/ADRA1A/KCNB2	5.975E-05	0.001617037
GO:0007221	Positive regulation of transcription of Notch receptor target	MAML2/NOTCH3/MAML3/NOTCH4	6.05514E-05	0.001627265
GO:0009306	Protein secretion	FRMD4A/ARNTL/DRD2/CACNA1C/LRP1/IFNG/NLGN2/PRKCA/MC4R/TGFB1/SNAP25/ABCG1/DPP4/IRS1/IL12B/BMP6/ICA1	6.50174E-05	0.001720474
GO:0050679	Positive regulation of epithelial cell proliferation	NRP1/HTRA1/MIR132/NF1/PRKCA/TGFA/SCN5A/FGF2/BMP6/FOXP2/PTN	6.56622E-05	0.001720474
GO:0007269	Neurotransmitter secretion	CACNB2/DRD2/HTR2A/NF1/SNAP25/SYN3/CAMK2A/CHRM2/DGKI/ADRA1A	6.58105E-05	0.001720474
GO:0099643	Signal release from synapse	CACNB2/DRD2/HTR2A/NF1/SNAP25/SYN3/CAMK2A/CHRM2/DGKI/ADRA1A	6.58105E-05	0.001720474
GO:0035592	Establishment of protein localization to extracellular region	FRMD4A/ARNTL/DRD2/CACNA1C/LRP1/IFNG/NLGN2/PRKCA/MC4R/TGFB1/SNAP25/ABCG1/DPP4/IRS1/IL12B/BMP6/ICA1	6.67636E-05	0.001733597
GO:0030879	Mammary gland development	CAPN1/STAT6/NCOR2/CYP19A1/ATP2C2/TGFBR2/SLC6A3/NOTCH4/NRG1	6.89435E-05	0.001778188
GO:0090276	Regulation of peptide hormone secretion	ARNTL/DRD2/CACNA1C/LRP1/IFNG/NLGN2/PRKCA/SNAP25/DPP4/IRS1/ICA1	7.14722E-05	0.001831117
GO:0035296	Regulation of tube diameter	PRKG1/KCNMA1/AVPR1A/HTR2A/CHRM3/SOD2/DOCK4/NOS3/ADRA1A	7.27954E-05	0.001840478
GO:0097746	Regulation of blood vessel diameter	PRKG1/KCNMA1/AVPR1A/HTR2A/CHRM3/SOD2/DOCK4/NOS3/ADRA1A	7.27954E-05	0.001840478
GO:0007625	Grooming behavior	DRD2/AVPR1A/MC4R/DDO	7.59573E-05	0.001892595
GO:0035150	Regulation of tube size	PRKG1/KCNMA1/AVPR1A/HTR2A/CHRM3/SOD2/DOCK4/NOS3/ADRA1A	7.68267E-05	0.001892595
GO:0106106	Cold-induced thermogenesis	ARNTL/STAT6/TRPV2/MAP2K6/PRDM16/GNAS/IL18R1/TRPM8/PDGFC	7.68267E-05	0.001892595
GO:0120161	Regulation of cold-induced thermogenesis	ARNTL/STAT6/TRPV2/MAP2K6/PRDM16/GNAS/IL18R1/TRPM8/PDGFC	7.68267E-05	0.001892595
GO:0070374	Positive regulation of ERK1 and ERK2 cascade	NRP1/DRD2/LRP1/HTR2A/MAP2K1/PRKCA/TGFB1/HCRTR1/FGF2/PDGFC/ADRA1A	7.77173E-05	0.001902341

GO:0002763	Positive regulation of myeloid leukocyte differentiation	IFNG/PRKCA/TGFB1/GNAS/RUNX1/IL12B	7.87574E-05	0.001915598
GO:0071692	Protein localization to extracellular region	FRMD4A/ARNTL/DRD2/CACNA1C/LRP1/IFNG/NLGN2/PRKCA/MC4R/TGFB1/SNAP25/ABCG1/DPP4/IRS1/IL12B/BMP6/ICA1	8.01902E-05	0.001938183
GO:0046209	Nitric oxide metabolic process	IFNG/RORA/SMAD3/TRPV1/MIR132/CX3CR1/NOS3	8.09083E-05	0.001943316
GO:0070509	Calcium ion import	CACNB2/CACNA1C/CACNA1H/ATP2C2/TRPV2/TRPV1/TRPM2	8.72544E-05	0.002082724
GO:0030072	Peptide hormone secretion	ARNTL/DRD2/CACNA1C/LRP1/IFNG/NLGN2/PRKCA/MC4R/SNAP25/DPP4/IRS1/ICA1	8.87529E-05	0.002096666
GO:0045785	Positive regulation of cell adhesion	NRP1/RSU1/SPOCK2/RAG1/IFNG/EFNB2/SMAD3/PRKCA/RUNX1/DPP4/TGFBR2/P2RY12/IL12B/HLA-DPA1/UTRN/PTN	8.89296E-05	0.002096666
GO:0007189	Adenylate cyclase-activating G protein-coupled receptor signaling pathway	DRD2/PRKCA/MC2R/MC4R/GALR1/CNR2/GNAS/RAMP1/ADRA1A	9.00643E-05	0.00211047
GO:0031644	Regulation of nervous system process	AVPR1A/BEGAIN/NLGN2/OPRD1/TNFRSF1B/SHANK3/SCN11A/GRM1/NOS3	9.48813E-05	0.002198089
GO:0099003	Vesicle-mediated transport in synapse	CACNB2/DRD2/HTR2A/EFNB2/NLGN2/SNAP25/SYN3/MAPK10/CHRM2/DGKI/ADRA1A	9.54102E-05	0.002198089
GO:0021537	Telencephalon development	DRD2/LRP1/AVPR1A/NF1/PLCB1/SHANK3/SCN5A/P2RY12/TACC3/PHACTR1/FOXP2/NRG1	9.55193E-05	0.002198089
GO:2001057	Reactive nitrogen species metabolic process	IFNG/RORA/SMAD3/TRPV1/MIR132/CX3CR1/NOS3	0.000101165	0.002314146
GO:0048545	Response to steroid hormone	ABCC2/ARNTL/AVPR1A/TPH2/NCOR2/ESR2/ESRRB/TGFB1/CASP9/RUNX1/TGFBR2/GABRB1/BMP6/PTN	0.000108073	0.002457546
GO:0050730	Regulation of peptidyl-tyrosine phosphorylation	NRP1/BDNF/IFNG/HTR2A/TGFB1/TGFA/EREG/PDGFC/IL12B/BMP6/NRG1/ADRA1A	0.000110393	0.00249554
GO:0010518	Positive regulation of phospholipase activity	BDNF/AVPR1A/HTR2A/P2RY12/FGF2/ADRA1A	0.000114804	0.002580078
GO:0050769	Positive regulation of neurogenesis	NRP1/BDNF/DRD2/LRP1/IFNG/MAP2K1/TRPV2/CSNK1D/TGFB1/TNFRSF1B/SHANK3/CX3CR1/P2RY12/LTA/BMP6/CUX1/PTN	0.000117224	0.002619144
GO:0042063	Gliogenesis	LRP1/IFNG/MAP2K1/NF1/PRX/TGFB1/TNFRSF1B/CX3CR1/P2RY12/LTA/PTN/NRG1/PENK	0.000121437	0.002685561
GO:0030336	Negative regulation of cell migration	PRKG1/DRD2/LRP1/CYP19A1/MIR132/NF1/TGFB1/ADORA3/PLCB1/DPP4/CX3CR1/FGF2/PTN/NRG1	0.000121925	0.002685561

GO:0045834	Positive regulation of lipid metabolic process	AVPR1A/IFNG/HTR2A/TGFB1/ABCG1/IRS1/P2RY12/FGF2/BMP6	0.000122334	0.002685561
GO:0051588	Regulation of neurotransmitter transport	CACNB2/DRD2/HTR2A/NF1/CAMK2A/CHRM2/DGKI/ADRA1A	0.000122992	0.002685561
GO:0098661	Inorganic anion transmembrane transport	SLC25A3/GABRB3/CLCN6/SLC12A5/GABRB1/ANKH/CLIC5/CLIC1	0.000130319	0.002829462
GO:0040013	Negative regulation of locomotion	NRP1/PRKG1/DRD2/LRP1/CYP19A1/MIR132/NF1/TGFB1/ADORA3/PLCB1/DPP4/CX3CR1/FGF2/PTN/NRG1	0.000131472	0.002838469
GO:0097191	Extrinsic apoptotic signaling pathway	NRP1/IFNG/CRADD/SMAD3/NF1/DCC/TGFB1/FAF1/CASP9/TNFRSF1B/NOS3	0.000141295	0.003016656
GO:0098657	Import into cell	DRD2/IFNG/EFNB2/TRPV2/TRPV1/SNAP25/SLC12A5/TRPM2/CACNG2/SLC39A8/SLC6A3	0.000141295	0.003016656
GO:0051271	Negative regulation of cellular component movement	NRP1/PRKG1/DRD2/LRP1/CYP19A1/MIR132/NF1/TGFB1/ADORA3/PLCB1/DPP4/CX3CR1/FGF2/PTN/NRG1	0.000142756	0.003030996
GO:0001666	Response to hypoxia	KCNMA1/DRD2/RORA/SMAD3/HMOX2/MMP2/NF1/OPRD1/DPP4/TGFBR2/LTA/KCND2/PTN/PENK	0.000158837	0.003353904
GO:0035270	Endocrine system development	ARNTL/DRD2/MAP2K1/SMAD3/NF1/SLC6A3/BMP6/TG	0.000163325	0.003429825
GO:1905954	Positive regulation of lipid localization	LRP1/CYP19A1/MAP2K6/GALR1/ABCG1/BMP6/PON1	0.000164744	0.003440827
GO:0001505	Regulation of neurotransmitter levels	CACNB2/DRD2/HTR2A/NF1/SNAP25/SYN3/SLC6A3/CAMK2A/CHRM2/DGKI/ADRA1A	0.000170533	0.003542485
GO:0003073	Regulation of systemic arterial blood pressure	PLCB3/DRD2/AVPR1A/TRPV1/SOD2/NOS3/ADRA1A	0.000176006	0.003597829
GO:0070167	Regulation of biomineral tissue development	SMAD3/TGFB1/ANKH/BMP6/ENPP1/PTN/NOS3	0.000176006	0.003597829
GO:0110149	Regulation of biomineralization	SMAD3/TGFB1/ANKH/BMP6/ENPP1/PTN/NOS3	0.000176006	0.003597829
GO:0043112	Receptor metabolic process	CAPN1/DRD2/LRP1/IFNG/EFNB2/TGFB1/SNAP25/CACNG2/RAMP1/NRG1	0.000182441	0.00370963
GO:2000146	Negative regulation of cell motility	PRKG1/DRD2/LRP1/CYP19A1/MIR132/NF1/TGFB1/ADORA3/PLCB1/DPP4/CX3CR1/FGF2/PTN/NRG1	0.000188502	0.003812704
GO:0014013	Regulation of gliogenesis	LRP1/NF1/TGFB1/TNFRSF1B/CX3CR1/P2RY12/LTA/PTN	0.000192355	0.003850114
GO:1903409	Reactive oxygen species biosynthetic process	IFNG/RORA/SMAD3/CYP1A2/TRPV1/MIR132/CX3CR1/NOS3	0.000192355	0.003850114

GO:0010038	Response to metal ion	KCNMA1/ABCC2/DLG2/DRD2/TPH2/CYP1A2/CACNA1H/CASP9/TRPM2/CACNG2/SCN5A/SLC6A3/BMP6/PENK	0.000193885	0.003860618
GO:0035710	CD4-positive, alpha-beta T cell activation	STAT6/IFNG/RORA/RUNX1/IL18R1/TGFBR2/IL12B	0.000200398	0.003937583
GO:0032881	Regulation of polysaccharide metabolic process	ESRRB/TGFB1/POMC/IRS1/ENPP1	0.000201848	0.003937583
GO:1903053	Regulation of extracellular matrix organization	LRP1/SMAD3/TGFB1/TNFRSF1B/DPP4	0.000201848	0.003937583
GO:1903539	Protein localization to postsynaptic membrane	DLG2/GRIN2A/SNAP25/CACNG2/MAPK10	0.000201848	0.003937583
GO:0051235	Maintenance of location	DRD2/CACNA1C/HTR2A/FTO/TRPV1/FAF1/NRIP1/ABCG1/TRPM2/FGF2/ENPP1/SYNE1/NPSR1	0.000206386	0.004005758
GO:0006835	Dicarboxylic acid transport	ABCC2/BDNF/AVPR1A/TRPV1/NF1/SNAP25/GRM7	0.000213579	0.004124538
GO:0036293	Response to decreased oxygen levels	KCNMA1/DRD2/RORA/SMAD3/HMOX2/MMP2/NF1/OPRD1/DPP4/TGFBR2/LTA/KCND2/PTN/PENK	0.000222835	0.004281766
GO:2001222	Regulation of neuron migration	CTNNA2/ULK4/CAMK2A/PHACTR1/NRG1	0.000224815	0.00429833
GO:0018108	Peptidyl-tyrosine phosphorylation	NRP1/BDNF/IFNG/HTR2A/MAP2K1/MAP2K6/TGFB1/TGFA/EREG/PDGFC/IL12B/BMP6/NRG1/ADRA1A	0.000241934	0.004602728
GO:0015800	Acidic amino acid transport	BDNF/AVPR1A/TRPV1/NF1/SNAP25/GRM7	0.000243884	0.004616978
GO:0072593	Reactive oxygen species metabolic process	MMP3/IFNG/RORA/SMAD3/CYP1A2/TRPV1/MIR132/TGFB1/CX3CR1/TGFBR2/SOD2/NOS3	0.000256322	0.004828661
GO:0043010	Camera-type eye development	NRP1/DRD2/CACNA1C/COL4A1/SMAD3/NF1/GPD2/TGFBR2/FGF2/SLC6A3/SDK1/FOXP2/PTN	0.000261452	0.004895639
GO:0018212	Peptidyl-tyrosine modification	NRP1/BDNF/IFNG/HTR2A/MAP2K1/MAP2K6/TGFB1/TGFA/EREG/PDGFC/IL12B/BMP6/NRG1/ADRA1A	0.000262426	0.004895639
GO:0043502	Regulation of muscle adaptation	SMAD3/PRKCA/TNFRSF1B/SCN5A/UTRN/NOS3/ADRA1A	0.000273474	0.005045698
GO:0055024	Regulation of cardiac muscle tissue development	FGF3/EFNB2/TGFB1/TGFBR2/VGLL4/FGF2/NRG1	0.000273474	0.005045698
GO:0045776	Negative regulation of blood pressure	DRD2/TRPV1/SOD2/NOS3/ADRA1A	0.00027666	0.005045698
GO:0032368	Regulation of lipid transport	LRP1/CYP19A1/MAP2K6/GALR1/ABCG1/POMC/BMP6/PON1	0.000276833	0.005045698

GO:0032496	Response to lipopolysaccharide	ABCC2/CYP1A2/PRKCA/TGFB1/CNR2/CASP9/TNFRSF1B/CX3CR1/IL12B/LTA/BMP6/NOS3/PENK	0.000277034	0.005045698
GO:0009612	Response to mechanical stimulus	DRD2/CRADD/HTR2A/CLCN6/SHANK3/TGFBR2/BMP6/FOXP2/PTN/TNC	0.000281999	0.005073783
GO:1905952	Regulation of lipid localization	LRP1/CYP19A1/FTO/MAP2K6/GALR1/ABCG1/POMC/BMP6/PON1	0.00028211	0.005073783
GO:0034776	Response to histamine	DRD2/GABRB3/GABRB1	0.000285178	0.005073783
GO:0086016	AV node cell action potential	CACNB2/CACNA1C/SCN5A	0.000285178	0.005073783
GO:0086027	AV node cell to bundle of His cell signaling	CACNB2/CACNA1C/SCN5A	0.000285178	0.005073783
GO:0048661	Positive regulation of smooth muscle cell proliferation	ABCC4/MMP2/NOTCH3/DNMT1/TGFBR2/EREG/FGF2	0.000290378	0.005118916
GO:0060079	Excitatory postsynaptic potential	DRD2/GRIN2B/GRIN2A/NLGN2/TRPV1/SHANK3/DGKI	0.000290378	0.005118916
GO:0045216	Cell-cell junction organization	EFNB2/SMAD3/NLGN2/PRKCA/PARD6G/TGFB1/RUNX1/BMP6/SDK1/SVEP1	0.000292954	0.005140737
GO:0062237	Protein localization to postsynapse	DLG2/GRIN2A/SNAP25/CACNG2/MAPK10	0.000305752	0.005316773
GO:1900271	Regulation of long-term synaptic potentiation	DRD2/NF1/SHANK3/CX3CR1/PTN	0.000305752	0.005316773
GO:0007214	Gamma-aminobutyric acid signaling pathway	BDNF/GABRB3/GABRB1/GABBR1	0.000318264	0.005484709
GO:0010996	Response to auditory stimulus	DRD2/SHANK3/FOXP2/PTN	0.000318264	0.005484709
GO:0051924	Regulation of calcium ion transport	CACNB2/DRD2/CACNA1C/ATP2C2/TRPV2/RGS9/OPRD1/P2RY12/CAMK2A/NPSR1/NOS3	0.00032202	0.00552465
GO:0043523	Regulation of neuron apoptotic process	NRP1/BDNF/LRP1/GABRB3/NF1/CASP9/CX3CR1/SOD2/OXR1/SIGMAR1	0.000340398	0.005799128
GO:0007409	Axonogenesis	NRP1/BDNF/DRD2/NCAM1/LRP1/EFNB2/ATL1/MAP2K1/TRPV2/PRKCA/DCC/NOTCH3/SHANK3/CTNNA2/RAPH1/GFRA2	0.000341469	0.005799128
GO:0034329	Cell junction assembly	NRP1/SPOCK2/BDNF/DRD2/LRP1/EFNB2/GABRB3/SMAD3/NLGN2/PRKCA/PARD6G/RUNX1/SHANK3/SDK1/NRG1	0.000342545	0.005799128
GO:0046632	Alpha-beta T cell differentiation	STAT6/IFNG/RORA/RUNX1/IL18R1/TGFBR2/IL12B	0.000346188	0.005835086

GO:0060193	Positive regulation of lipase activity	BDNF/AVPR1A/HTR2A/P2RY12/FGF2/ADRA1A	0.0003537	0.00593568
GO:1903727	Positive regulation of phospholipid metabolic process	HTR2A/TGFB1/IRS1/P2RY12/FGF2	0.000370831	0.0061308
GO:0001976	Nervous system process involved in regulation of systemic arterial blood pressure	DRD2/SOD2/ADRA1A	0.000376776	0.0061308
GO:0038003	Opioid receptor signaling pathway	OPRD1/OPRK1/SIGMAR1	0.000376776	0.0061308
GO:0048148	Behavioral response to cocaine	DRD2/HTR2A/SDK1	0.000376776	0.0061308
GO:0086067	AV node cell to bundle of His cell communication	CACNB2/CACNA1C/SCN5A	0.000376776	0.0061308
GO:0048771	Tissue remodeling	CAPN1/MMP2/NF1/PRKCA/MC4R/TGFB1/IL12B/PTN/NOS3	0.000379056	0.0061308
GO:0051260	Protein homooligomerization	ATL1/TRPV1/RNF213/KCND3/TRPM2/SOD2/KCND2/KCNB2/SIGMAR1	0.000379056	0.0061308
GO:0007422	Peripheral nervous system development	BDNF/SCN8A/NF1/PRX/RUNX1/NRG1	0.000379685	0.0061308
GO:0030500	Regulation of bone mineralization	SMAD3/TGFB1/ANKH/BMP6/ENPP1/PTN	0.000379685	0.0061308
GO:0006869	Lipid transport	ABCC2/DRD2/LRP1/ABCC4/CYP19A1/MAP2K6/GALR1/ABCG1/APOL3/POMC/SLC10A7/BMP6/PON1/SIGMAR1	0.000398812	0.006412694
GO:0043367	CD4-positive, alpha-beta T cell differentiation	STAT6/IFNG/RORA/RUNX1/IL18R1/IL12B	0.000407139	0.006510016
GO:0045598	Regulation of fat cell differentiation	ARNTL/HTR2A/RORA/SMAD3/FTO/TGFB1/PRDM16/ENPP1	0.000408252	0.006510016
GO:0008361	Regulation of cell size	NRP1/KCNMA1/BDNF/LRP1/TRPV2/DCC/CLCN6/SLC12A5/SHANK3	0.000428274	0.006774251
GO:0070482	Response to oxygen levels	KCNMA1/DRD2/RORA/SMAD3/HMOX2/MMP2/NF1/OPRD1/DPP4/TGFBR2/LTA/KCND2/PTN/PENK	0.000430196	0.006774251
GO:0061448	Connective tissue development	FGF6/SPTLC2/SMAD3/ACAN/FTO/TGFB1/GNAS/RUNX1/TGFBR2/FGF2/BMP6	0.000432779	0.006774251
GO:0071867	Response to monoamine	DRD2/HTR2A/RGS9/CHRM3/GNAS/CHRM2/PENK	0.000433637	0.006774251

GO:0071869	Response to catecholamine	DRD2/HTR2A/RGS9/CHRM3/GNAS/CHRM2/PENK	0.000433637	0.006774251
GO:0043536	Positive regulation of blood vessel endothelial cell migration	NRP1/MIR132/PRKCA/TGFB1/FGF2/NOS3	0.000436118	0.006785438
GO:0051339	Regulation of lyase activity	DRD2/CACNA1C/NF1/GALR1/NOS3	0.000445866	0.006909132
GO:0043406	Positive regulation of MAP kinase activity	TAOK3/HTR2A/MAP2K1/MAP2K6/TGFB1/TGFA/MAPK10/FGF2/PDGFC/GRM1/NRG1	0.000461305	0.00709794
GO:1903034	Regulation of response to wounding	PRKG1/LRP1/MAP2K1/SMAD3/PRKCA/AJAP1/FGF2/PTN/NOS3	0.000463919	0.00709794
GO:0048678	Response to axon injury	DRD2/LRP1/MAP2K1/P2RY12/PTN/TNC	0.000466682	0.00709794
GO:2000300	Regulation of synaptic vesicle exocytosis	CACNB2/DRD2/HTR2A/CHRM2/DGKI/ADRA1A	0.000466682	0.00709794
GO:0048813	Dendrite morphogenesis	NRP1/KNDC1/SHANK3/CTNNA2/KIF1A/PHACTR1/CUX1/PTN	0.00046901	0.00709794
GO:0048638	Regulation of developmental growth	NRP1/TLL2/BDNF/DRD2/LRP1/FTO/TRPV2/DCC/PLCB1/TGFBR2/VGLL4/FGF2/SLC6A3	0.000469133	0.00709794
GO:0007416	Synapse assembly	SPOCK2/BDNF/DRD2/EFNB2/GABRB3/NLGN2/SHANK3/SDK1/NRG1	0.000482636	0.007119117
GO:0006884	Cell volume homeostasis	KCNMA1/CLCN6/SLC12A5/SHANK3	0.000482769	0.007119117
GO:0030002	Cellular anion homeostasis	ABCC2/SLC12A5/ENPP1	0.000485352	0.007119117
GO:0030320	Cellular monovalent inorganic anion homeostasis	ABCC2/SLC12A5/ENPP1	0.000485352	0.007119117
GO:0031223	Auditory behavior	DRD2/SHANK3/FOXP2	0.000485352	0.007119117
GO:0035641	Locomotory exploration behavior	NLGN2/DPP4/PENK	0.000485352	0.007119117
GO:0051481	Negative regulation of cytosolic calcium ion concentration	DRD2/LRP1/SMAD3	0.000485352	0.007119117
GO:1903977	Positive regulation of glial cell migration	LRP1/CX3CR1/P2RY12	0.000485352	0.007119117
GO:0045599	Negative regulation of fat cell differentiation	ARNTL/RORA/SMAD3/TGFB1/ENPP1	0.000487412	0.007122149

GO:0002237	Response to molecule of bacterial origin	ABCC2/CYP1A2/PRKCA/TGFB1/CNR2/CASP9/TNFRSF1B/CX3CR1/IL12B/LTA/BMP6/NOS3/PENK	0.000507955	0.007394213
GO:0008277	Regulation of G protein-coupled receptor signaling pathway	GRK5/DRD2/RGS9/PRKCA/PLCB1/RAMP1/RGS12/CAMK2A	0.000513445	0.007445924
GO:0098693	Regulation of synaptic vesicle cycle	CACNB2/DRD2/HTR2A/NLGN2/CHRM2/DGKI/ADRA1A	0.000537893	0.007771142
GO:0008217	Regulation of blood pressure	PLCB3/DRD2/AVPR1A/SMAD3/TRPV1/POMC/SOD2/NOS3/ADRA1A	0.000542545	0.007779858
GO:0048639	Positive regulation of developmental growth	NRP1/BDNF/DRD2/LRP1/TRPV2/PLCB1/TGFBR2/FGF2/SLC6A3	0.000542545	0.007779858
GO:0007517	Muscle organ development	TLL2/ARNTL/FGF3/EFNB2/SMAD3/CACNA1H/NF1/TGFB1/TGFBR2/VGLL4/FGF2/UTRN/FOXP2/NRG1	0.000563974	0.008057078
GO:0050886	Endocrine process	AVPR1A/GALR1/GNAS/POMC/BMP6/NOS3	0.000605984	0.008616552
GO:0051048	Negative regulation of secretion	FRMD4A/DRD2/NF1/TNFRSF1B/IRS1/GRM7/P2RY12/IL12B/NRG1	0.000608423	0.008616552
GO:0014854	Response to inactivity	DRD2/SCN5A/UTRN	0.000612105	0.008616552
GO:1903054	Negative regulation of extracellular matrix organization	TGFB1/TNFRSF1B/DPP4	0.000612105	0.008616552
GO:0051259	Protein complex oligomerization	GRIN2B/ATL1/TRPV1/RNF213/KCND3/TRPM2/SOD2/KCND2/KCNB2/SIGMAR1	0.000619612	0.008683098
GO:0045761	Regulation of adenylate cyclase activity	DRD2/CACNA1C/NF1/GALR1	0.000621351	0.008683098
GO:0030282	Bone mineralization	SMAD3/TGFB1/SLC24A3/ANKH/BMP6/ENPP1/PTN	0.000628451	0.008750493
GO:0035418	Protein localization to synapse	DLG2/GRIN2A/NLGN2/SNAP25/CACNG2/MAPK10	0.000645381	0.00895379
GO:0021987	Cerebral cortex development	LRP1/NF1/PLCB1/P2RY12/TACC3/PHACTR1/FOXP2	0.000661186	0.009089904
GO:0051209	Release of sequestered calcium ion into cytosol	DRD2/CACNA1C/HTR2A/TRPV1/TRPM2/FGF2/NPSR1	0.000661186	0.009089904
GO:0009266	Response to temperature stimulus	ABCC2/HTR2A/TRPV2/TRPV1/NF1/SLC12A5/TRPM2/TRPM8/CAMK2A/NOS3	0.000662288	0.009089904
GO:0062013	Positive regulation of small molecule metabolic process	AVPR1A/IFNG/HTR2A/ESRRB/ABCG1/IRS1/BMP6/NOS3	0.000667603	0.009130245

GO:0097305	Response to alcohol	DRD2/GRIN2B/AVPR1A/GRIN2A/TGFB1/TGFBR2/SLC6A3/AHR/PENK/TNC	0.000684519	0.009325518
GO:0006112	Energy reserve metabolic process	ESRRB/MC4R/GNAS/POMC/IRS1/ENPP1	0.000686735	0.009325518
GO:0051283	Negative regulation of sequestering of calcium ion	DRD2/CACNA1C/HTR2A/TRPV1/TRPM2/FGF2/NPSR1	0.00069526	0.009408042
GO:0001975	Response to amphetamine	DRD2/GRIN2A/RGS9/CNR2	0.00070026	0.009409435
GO:0043552	Positive regulation of phosphatidylinositol 3-kinase activity	TGFB1/IRS1/P2RY12/FGF2	0.00070026	0.009409435
GO:0045444	Fat cell differentiation	GRK5/ARNTL/HTR2A/RORA/SMAD3/FTO/TGFB1/PRDM16/PLCB1/ENPP1	0.000707363	0.009438877
GO:1904018	Positive regulation of vasculature development	NRP1/EFNB2/MIR132/PRKCA/RUNX1/CX3CR1/TGFBR2/FGF2/SOD2/NOS3	0.000707363	0.009438877
GO:0030512	Negative regulation of transforming growth factor beta receptor signaling pathway	HTRA1/SMAD3/TGFB1/PRDM16/TGFBR2/VEPH1	0.00073011	0.009708698
GO:0007638	Mechanosensory behavior	DRD2/SHANK3/FOXP2	0.000758179	0.009978357
GO:0060134	Prepulse inhibition	DRD2/CTNNA2/SLC6A3	0.000758179	0.009978357
GO:2000846	Regulation of corticosteroid hormone secretion	GALR1/POMC/BMP6	0.000758179	0.009978357
GO:0016079	Synaptic vesicle exocytosis	CACNB2/DRD2/HTR2A/SNAP25/CHRM2/DGKI/ADRA1A	0.000767576	0.010033319
GO:0051282	Regulation of sequestering of calcium ion	DRD2/CACNA1C/HTR2A/TRPV1/TRPM2/FGF2/NPSR1	0.000767576	0.010033319
GO:0042311	Vasodilation	PRKG1/KCNMA1/SOD2/NOS3	0.000786004	0.010194439
GO:0045907	Positive regulation of vasoconstriction	AVPR1A/HTR2A/CHRM3/ADRA1A	0.000786004	0.010194439
GO:0051216	Cartilage development	FGF6/SMAD3/ACAN/TGFB1/GNAS/RUNX1/TGFBR2/FGF2/BMP6	0.000787861	0.010194439
GO:0042752	Regulation of circadian rhythm	ARNTL/KDM2A/DRD2/MTA1/RORA/CSNK1D/MAPK10	0.000805898	0.010392842

GO:1902107	Positive regulation of leukocyte differentiation	RAG1/IFNG/PRKCA/TGFB1/GNAS/RUNX1/TGFBR2/IL12B	0.000822834	0.010545122
GO:1903845	Negative regulation of cellular response to transforming growth factor beta stimulus	HTRA1/SMAD3/TGFB1/PRDM16/TGFBR2/VEPH1	0.000823195	0.010545122
GO:0002761	Regulation of myeloid leukocyte differentiation	IFNG/NF1/PRKCA/TGFB1/GNAS/RUNX1/IL12B	0.000845716	0.010770237
GO:0099504	Synaptic vesicle cycle	CACNB2/DRD2/HTR2A/NLGN2/SNAP25/SYN3/CHRM2/DGKI/ADRA1A	0.000846373	0.010770237
GO:0001655	Urogenital system development	NRP1/BDNF/EFNB2/COL4A1/CYP19A1/SMAD3/NF1/NOTCH3/CASP9/FGF2/BMP6/TNC	0.000860623	0.010863205
GO:0019229	Regulation of vasoconstriction	AVPR1A/HTR2A/CHRM3/DOCK4/ADRA1A	0.000864986	0.010863205
GO:0061098	Positive regulation of protein tyrosine kinase activity	BDNF/TGFA/EREG/NRG1/ADRA1A	0.000864986	0.010863205
GO:0097755	Positive regulation of blood vessel diameter	PRKG1/KCNMA1/SOD2/DOCK4/NOS3	0.000864986	0.010863205
GO:1903351	Cellular response to dopamine	DRD2/HTR2A/RGS9/CHRM3/GNAS/CHRM2	0.000873039	0.010891427
GO:1901654	Response to ketone	ABCC2/AVPR1A/TGFB1/CASP9/GABRB1/AHR/FOXP2/PTN/TNC	0.000876928	0.010891427
GO:0034390	Smooth muscle cell apoptotic process	IFNG/DNMT1/IL12B/SOD2	0.000878903	0.010891427
GO:0034391	Regulation of smooth muscle cell apoptotic process	IFNG/DNMT1/IL12B/SOD2	0.000878903	0.010891427
GO:0032147	Activation of protein kinase activity	DRD2/TAOK3/MAP2K1/MAP2K6/TGFA/TGFBR2/MAPK10/FGF2/PDGFC/IL12B/GRM1/NRG1	0.000883444	0.010891427
GO:0022612	Gland morphogenesis	NRP1/CAPN1/STAT6/TGFB1/TGFBR2/PTN/TNC	0.000887072	0.010891427
GO:0051208	Sequestering of calcium ion	DRD2/CACNA1C/HTR2A/TRPV1/TRPM2/FGF2/NPSR1	0.000887072	0.010891427
GO:0035930	Corticosteroid hormone secretion	GALR1/POMC/BMP6	0.000924669	0.011251454
GO:0099170	Postsynaptic modulation of chemical synaptic transmission	DRD2/DCC/PLCB1	0.000924669	0.011251454

GO:1903350	Response to dopamine	DRD2/HTR2A/RGS9/CHRM3/GNAS/CHRM2	0.000925178	0.011251454
GO:0043500	Muscle adaptation	SMAD3/PRKCA/TNFRSF1B/SCN5A/UTRN/NOS3/ADRA1A	0.000930006	0.011273559
GO:0045843	Negative regulation of striated muscle tissue development	TLL2/FGF3/TGFB1/TGFBR2/VGLL4	0.000932863	0.011273559
GO:0031214	Biomineral tissue development	SMAD3/TGFB1/SLC24A3/ANKH/BMP6/ENPP1/PTN/NOS3	0.000966848	0.011611245
GO:0110148	Biomineralization	SMAD3/TGFB1/SLC24A3/ANKH/BMP6/ENPP1/PTN/NOS3	0.000966848	0.011611245
GO:0051402	Neuron apoptotic process	NRP1/BDNF/LRP1/GABRB3/NF1/CASP9/CX3CR1/SOD2/OXR1/SIGMAR1	0.000972406	0.01164161
GO:0001569	Branching involved in blood vessel morphogenesis	NRP1/COL4A1/TGFBR2/NOTCH4	0.000979275	0.011651246
GO:0070873	Regulation of glycogen metabolic process	ESRRB/POMC/IRS1/ENPP1	0.000979275	0.011651246
GO:0002294	CD4-positive, alpha-beta T cell differentiation involved in immune response	STAT6/IFNG/RORA/IL18R1/IL12B	0.001004622	0.011770621
GO:0007405	Neuroblast proliferation	BDNF/DRD2/NF1/CX3CR1/PTN	0.001004622	0.011770621
GO:0048635	Negative regulation of muscle organ development	TLL2/FGF3/TGFB1/TGFBR2/VGLL4	0.001004622	0.011770621
GO:0055081	Anion homeostasis	ABCC2/MIR132/SLC12A5/ABCG1/ENPP1	0.001004622	0.011770621
GO:0070527	Platelet aggregation	PRKG1/PRKCA/GNAS/P2RY12/CLIC1	0.001004622	0.011770621
GO:0072089	Stem cell proliferation	BDNF/DRD2/NF1/RUNX1/CX3CR1/FGF2/PTN	0.001068705	0.012483382
GO:0002287	Alpha-beta T cell activation involved in immune response	STAT6/IFNG/RORA/IL18R1/IL12B	0.001080401	0.012513594
GO:0002293	Alpha-beta T cell differentiation involved in immune response	STAT6/IFNG/RORA/IL18R1/IL12B	0.001080401	0.012513594
GO:0090218	Positive regulation of lipid kinase activity	TGFB1/IRS1/P2RY12/FGF2	0.00108744	0.012513594

GO:0098926	Postsynaptic signal transduction	CHRM3/PLCB1/CHRM2/NRG1	0.00108744	0.012513594
GO:1903531	Negative regulation of secretion by cell	FRMD4A/DRD2/NF1/TNFRSF1B/IRS1/GRM7/P2RY12/IL12B	0.001087572	0.012513594
GO:0045778	Positive regulation of ossification	SMAD3/TGFB1/GNAS/CLIC1/BMP6/PTN	0.001096066	0.012573673
GO:1990778	Protein localization to cell periphery	CACNB2/DLG2/LRP1/IFNG/GRIN2A/ATP2C2/TGFB1/SNAP25/CACNG2/RAMP1/MAPK10/KCNB2	0.001112529	0.012612996
GO:0010715	Regulation of extracellular matrix disassembly	LRP1/TGFB1/DPP4	0.001112622	0.012612996
GO:0015732	Prostaglandin transport	ABCC2/ABCC4/MAP2K6	0.001112622	0.012612996
GO:0045986	Negative regulation of smooth muscle contraction	PRKG1/KCNMA1/DOCK4	0.001112622	0.012612996
GO:0000041	Transition metal ion transport	ABCC2/IFNG/SLC39A9/ATP2C2/TRPM2/CP/SLC39A8	0.001118381	0.012640991
GO:0014706	Striated muscle tissue development	TLL2/ARNTL/FGF3/EFNB2/SMAD3/NF1/TGFB1/TGFBR2/VGLL4/FGF2/FOXP2/NRG1/ADRA1A	0.001147794	0.012935402
GO:1901862	Negative regulation of muscle tissue development	TLL2/FGF3/TGFB1/TGFBR2/VGLL4	0.001160335	0.013038505
GO:0043405	Regulation of MAP kinase activity	TAOK3/HTR2A/MAP2K1/NF1/MAP2K6/TGFB1/TGFA/MAPK10/FGF2/PDGFC/GRM1/NRG1	0.001169624	0.013084411
GO:0031331	Positive regulation of cellular catabolic process	LRP1/IFNG/HTR2A/ESRRB/SPTLC2/TSC2/FTO/CSNK1D/FAF1/TNFRSF1B/IRS1/VGLL4/RNF144B	0.001174635	0.013084411
GO:0042692	Muscle cell differentiation	BDNF/AVPR1A/EFNB2/RORA/CACNA1H/DNMT1/TGFB1/CTNNA2/EREG/SYNE1/SOD2/NRG1/ADRA1A	0.001174635	0.013084411
GO:0010661	Positive regulation of muscle cell apoptotic process	IFNG/CAMK2A/IL12B/SOD2	0.001203717	0.013331084
GO:0032885	Regulation of polysaccharide biosynthetic process	ESRRB/TGFB1/IRS1/ENPP1	0.001203717	0.013331084
GO:0007588	Excretion	KCNMA1/DRD2/AVPR1A/TRPV1/ADRA1A	0.001244565	0.013743861
GO:0071902	Positive regulation of protein serine/threonine kinase activity	IFNG/TAOK3/HTR2A/MAP2K1/MAP2K6/TGFB1/TGFA/MAPK10/FGF2/PDGFC/GRM1/NRG1	0.001259813	0.013872385
GO:0001101	Response to acid chemical	AVPR1A/COL4A1/MMP2/DNMT1/MAPK10/PDGFC/PTN	0.001278357	0.01400613

GO:0098659	Inorganic cation import across plasma membrane	IFNG/TRPV2/TRPV1/SLC12A5/TRPM2/SLC39A8	0.001290182	0.01400613
GO:0099587	Inorganic ion import across plasma membrane	IFNG/TRPV2/TRPV1/SLC12A5/TRPM2/SLC39A8	0.001290182	0.01400613
GO:0120162	Positive regulation of cold-induced thermogenesis	STAT6/TRPV2/PRDM16/GNAS/TRPM8/PDGFC	0.001290182	0.01400613
GO:1902476	Chloride transmembrane transport	GABRB3/CLCN6/SLC12A5/GABRB1/CLIC5/CLIC1	0.001290182	0.01400613
GO:1901342	Regulation of vasculature development	NRP1/EFNB2/MIR132/NF1/PRKCA/DNMT1/RUNX1/CX3CR1/TGFBR2/FGF2/IL12B/SOD2/PTN/NOS3	0.001302112	0.014095825
GO:0021756	Striatum development	DRD2/SHANK3/FOXP2	0.001323036	0.014141668
GO:0060004	Reflex	CACNG2/FOXP2/ADRA1A	0.001323036	0.014141668
GO:0060080	Inhibitory postsynaptic potential	BDNF/GABRB3/NLGN2	0.001323036	0.014141668
GO:2000831	Regulation of steroid hormone secretion	GALR1/POMC/BMP6	0.001323036	0.014141668
GO:0001662	Behavioral fear response	RAG1/BDNF/DPP4/PENK	0.001328426	0.014141668
GO:0086091	Regulation of heart rate by cardiac conduction	CACNB2/CACNA1C/KCND3/SCN5A	0.001328426	0.014141668
GO:0050714	Positive regulation of protein secretion	FRMD4A/DRD2/LRP1/IFNG/NLGN2/TGFB1/ABCG1/BMP6	0.001365092	0.014491849
GO:0001819	Positive regulation of cytokine production	DRD2/STAT6/IFNG/RORA/SMAD3/TGFB1/PLCB1/RUNX1/IL18R1/EREG/IL12B/POLR1C/HLA-DPA1/LTA	0.001387115	0.014685075
GO:0050918	Positive chemotaxis	NRP1/LRP1/SMAD3/TSC2/FGF2	0.00142647	0.01506023
GO:1901617	Organic hydroxy compound biosynthetic process	IFNG/TPH2/SPTLC2/CYP19A1/HDC/CACNA1H/ABCG1/FGF2/SLC6A3/BMP6	0.001433598	0.015094018
GO:0002209	Behavioral defense response	RAG1/BDNF/DPP4/PENK	0.001461884	0.015266361
GO:0030501	Positive regulation of bone mineralization	SMAD3/TGFB1/BMP6/PTN	0.001461884	0.015266361
GO:1902895	Positive regulation of pri-mirna transcription by RNA polymerase II	TEAD1/SMAD3/TGFB1/FGF2	0.001461884	0.015266361

GO:0051099	Positive regulation of binding	NRP1/BDNF/SPON1/LRP1/IFNG/TGFB1/RAMP1/PON1	0.001469173	0.015300895
GO:0045428	Regulation of nitric oxide biosynthetic process	IFNG/SMAD3/TRPV1/MIR132/CX3CR1	0.001524427	0.015790767
GO:0099072	Regulation of postsynaptic membrane neurotransmitter receptor levels	DLG2/EFNB2/SNAP25/CACNG2/MAPK10	0.001524427	0.015790767
GO:0070372	Regulation of ERK1 and ERK2 cascade	NRP1/DRD2/LRP1/HTR2A/MAP2K1/PRKCA/TGFB1/HCRTR1/FGF2/PDGFC/ADRA1A	0.001537036	0.015870092
GO:0010810	Regulation of cell-substrate adhesion	NRP1/RSU1/SPOCK2/LRP1/SMAD3/NF1/AJAP1/UTRN/PTN	0.001549173	0.015870092
GO:0007213	G protein-coupled acetylcholine receptor signaling pathway	CHRM3/PLCB1/CHRM2	0.001556863	0.015870092
GO:0034393	Positive regulation of smooth muscle cell apoptotic process	IFNG/IL12B/SOD2	0.001556863	0.015870092
GO:1903975	Regulation of glial cell migration	LRP1/CX3CR1/P2RY12	0.001556863	0.015870092
GO:2000647	Negative regulation of stem cell proliferation	BDNF/NF1/PTN	0.001556863	0.015870092
GO:0010634	Positive regulation of epithelial cell migration	NRP1/IFNG/MIR132/PRKCA/TGFB1/TGFBR2/FGF2/NOS3	0.001579457	0.016057815
GO:0042552	Myelination	SCN8A/NF1/PRX/TNFRSF1B/PTN/NRG1/TG	0.001584229	0.016063834
GO:0045639	Positive regulation of myeloid cell differentiation	IFNG/PRKCA/TGFB1/GNAS/RUNX1/IL12B	0.001588623	0.016065996
GO:0009896	Positive regulation of catabolic process	LRP1/IFNG/HTR2A/ESRRB/SPTLC2/TSC2/FTO/WWP2/CSNK1D/FAF1/TNFRSF1B/IRS1/VGLL4/RNF144B	0.001603658	0.01614068
GO:0042596	Fear response	RAG1/BDNF/DPP4/PENK	0.001604408	0.01614068
GO:0048662	Negative regulation of smooth muscle cell proliferation	PRKG1/IFNG/IL12B/SOD2/NOS3	0.001627245	0.016327682
GO:0030324	Lung development	MAP2K1/CYP1A2/TGFBR2/FGF2/FOXP2/PTN/NOS3/TNC	0.001637007	0.016382855
GO:0007272	Ensheathment of neurons	SCN8A/NF1/PRX/TNFRSF1B/PTN/NRG1/TG	0.001721415	0.017138337

GO:0008366	Axon ensheathment	SCN8A/NF1/PRX/TNFRSF1B/PTN/NRG1/TG	0.001721415	0.017138337
GO:0006879	Cellular iron ion homeostasis	IFNG/HMOX2/CP/SLC39A8/BMP6	0.001735066	0.017229607
GO:0060537	Muscle tissue development	TLL2/ARNTL/FGF3/EFNB2/SMAD3/NF1/TGFB1/TGFBR2/VGLL4/FGF2/FOXP2/NRG1/ADRA1A	0.001793218	0.017613849
GO:0019722	Calcium-mediated signaling	GRIN2B/CACNA1C/AVPR1A/GRIN2A/CHRM3/TRPM2/CX3CR1/P2RY12/NRG1	0.001810857	0.017613849
GO:0003085	Negative regulation of systemic arterial blood pressure	TRPV1/SOD2/ADRA1A	0.00181501	0.017613849
GO:0010875	Positive regulation of cholesterol efflux	LRP1/ABCG1/PON1	0.00181501	0.017613849
GO:0048670	Regulation of collateral sprouting	BDNF/LRP1/DCC	0.00181501	0.017613849
GO:0086014	Atrial cardiac muscle cell action potential	CACNB2/CACNA1C/SCN5A	0.00181501	0.017613849
GO:0086026	Atrial cardiac muscle cell to AV node cell signaling	CACNB2/CACNA1C/SCN5A	0.00181501	0.017613849
GO:0086066	Atrial cardiac muscle cell to AV node cell communication	CACNB2/CACNA1C/SCN5A	0.00181501	0.017613849
GO:0098703	Calcium ion import across plasma membrane	TRPV2/TRPV1/TRPM2	0.00181501	0.017613849
GO:0043279	Response to alkaloid	DRD2/HTR2A/TRPV1/SLC6A3/SDK1/PENK	0.001844867	0.017844209
GO:0002292	T cell differentiation involved in immune response	STAT6/IFNG/RORA/IL18R1/IL12B	0.001848034	0.017844209
GO:0030323	Respiratory tube development	MAP2K1/CYP1A2/TGFBR2/FGF2/FOXP2/PTN/NOS3/TNC	0.00188402	0.018146083
GO:0098656	Anion transmembrane transport	SLC25A3/SLC24A4/GABRB3/CLCN6/SLC24A3/SLC12A5/GABRB1/SLC39A8/ANKH/CLIC5/CLIC1	0.001930503	0.018515964
GO:0021549	Cerebellum development	KNDC1/RORA/MAP2K1/SCN5A/FOXP2/PTN	0.001936877	0.018515964
GO:1902414	Protein localization to cell junction	DLG2/GRIN2A/NLGN2/SNAP25/CACNG2/MAPK10	0.001936877	0.018515964
GO:0090288	Negative regulation of cellular response to growth factor stimulus	HTRA1/SMAD3/TGFB1/PRDM16/TGFBR2/VEPH1/FGF2/IL12B	0.001950144	0.018596538

GO:0010631	Epithelial cell migration	NRP1/IFNG/EFNB2/MIR132/NF1/PRKCA/TGFB1/DPP4/TGFBR2/FGF2/PTN/NOS3	0.002020095	0.019215908
GO:0071868	Cellular response to monoamine stimulus	DRD2/HTR2A/RGS9/CHRM3/GNAS/CHRM2	0.002032318	0.019236943
GO:0071870	Cellular response to catecholamine stimulus	DRD2/HTR2A/RGS9/CHRM3/GNAS/CHRM2	0.002032318	0.019236943
GO:0044060	Regulation of endocrine process	GALR1/GNAS/POMC/BMP6	0.002089519	0.01957262
GO:0010893	Positive regulation of steroid biosynthetic process	IFNG/ABCG1/BMP6	0.00209834	0.01957262
GO:0030728	Ovulation	NRIP1/EREG/NOS3	0.00209834	0.01957262
GO:0030878	Thyroid gland development	MAP2K1/SMAD3/TG	0.00209834	0.01957262
GO:0035929	Steroid hormone secretion	GALR1/POMC/BMP6	0.00209834	0.01957262
GO:1902656	Calcium ion import into cytosol	TRPV2/TRPV1/TRPM2	0.00209834	0.01957262
GO:0007568	Aging	ARNTL/LRP1/HTR2A/MAP2K1/CASP9/TNFRSF1B/TGFBR2/SLC6A3/SOD2/ADRA1A/PENK	0.002129938	0.019783767
GO:0050905	Neuromuscular process	DRD2/GRIN2A/NLGN2/CTNNA2/SLC6A3/PENK	0.002131272	0.019783767
GO:0090132	Epithelium migration	NRP1/IFNG/EFNB2/MIR132/NF1/PRKCA/TGFB1/DPP4/TGFBR2/FGF2/PTN/NOS3	0.00216125	0.020013696
GO:2001259	Positive regulation of cation channel activity	CACNB2/IFNG/RGS9/CACNG2/SHANK3	0.002219274	0.020405961
GO:0045927	Positive regulation of growth	NRP1/BDNF/DRD2/LRP1/AVPR1A/TRPV2/PLCB1/TGFBR2/FGF2/SLC6A3	0.00223061	0.020405961
GO:0051147	Regulation of muscle cell differentiation	BDNF/EFNB2/DNMT1/TGFB1/CTNNA2/EREG/SOD2/NRG1	0.00223316	0.020405961
GO:0002062	Chondrocyte differentiation	SMAD3/ACAN/TGFB1/RUNX1/TGFBR2/BMP6	0.002233821	0.020405961
GO:0008593	Regulation of Notch signaling pathway	MAML2/WWP2/NOTCH3/MAML3/NOTCH4/NOS3	0.002233821	0.020405961
GO:0050920	Regulation of chemotaxis	NRP1/EFNB2/CYP19A1/SMAD3/TGFB1/DPP4/P2RY12/FGF2/PTN	0.00223547	0.020405961

GO:0046631	Alpha-beta T cell activation	STAT6/IFNG/RORA/RUNX1/IL18R1/TGFBR2/IL12B	0.00227556	0.020722694
GO:0010001	Glial cell differentiation	LRP1/IFNG/MAP2K1/NF1/PRX/TGFB1/TNFRSF1B/PTN/NRG1	0.002302107	0.02091489
GO:0018958	Phenol-containing compound metabolic process	DRD2/TPH2/HDC/GRIN2A/SLC6A3/TG	0.002340047	0.021209431
GO:0048844	Artery morphogenesis	NRP1/LRP1/EFNB2/NF1/NOTCH3	0.002354284	0.021288266
GO:0061337	Cardiac conduction	CACNB2/CACNA1C/KCND3/CACNG2/CACNA2D3/SCN5A/KCND2	0.002456708	0.022055481
GO:0070371	ERK1 and ERK2 cascade	NRP1/DRD2/LRP1/HTR2A/MAP2K1/PRKCA/TGFB1/HCRTR1/FGF2/PDGFC/ADRA1A	0.002460226	0.022055481
GO:0006367	Transcription initiation from RNA polymerase II promoter	TEAD1/MAML2/ESR2/ESRRB/RORA/NOTCH3/MAML3/NOTCH4	0.002465849	0.022055481
GO:0043534	Blood vessel endothelial cell migration	NRP1/EFNB2/MIR132/NF1/PRKCA/TGFB1/FGF2/NOS3	0.002465849	0.022055481
GO:0090130	Tissue migration	NRP1/IFNG/EFNB2/MIR132/NF1/PRKCA/TGFB1/DPP4/TGFBR2/FGF2/PTN/NOS3	0.002467826	0.022055481
GO:0015980	Energy derivation by oxidation of organic compounds	IFNG/ESRRB/CYP1A2/MC4R/PRDM16/GNAS/POMC/GPD2/IRS1/ENPP1	0.00247727	0.022088514
GO:0008306	Associative learning	RAG1/DRD2/GRIN2A/NF1/SNAP25	0.00249517	0.022094329
GO:0071229	Cellular response to acid chemical	AVPR1A/COL4A1/MMP2/DNMT1/PDGFC	0.00249517	0.022094329
GO:0072091	Regulation of stem cell proliferation	BDNF/DRD2/NF1/CX3CR1/PTN	0.00249517	0.022094329
GO:0043524	Negative regulation of neuron apoptotic process	NRP1/BDNF/LRP1/GABRB3/CX3CR1/SOD2/OXR1	0.002551346	0.022539823
GO:0006821	Chloride transport	GABRB3/CLCN6/SLC12A5/GABRB1/CLIC5/CLIC1	0.00256386	0.022598425
GO:0150076	Neuroinflammatory response	MMP3/LRP1/IFNG/TRPV1/TNFRSF1B	0.002642079	0.023140058
GO:0008542	Visual learning	RAG1/DRD2/GRIN2A/NF1	0.002667459	0.023140058
GO:0014075	Response to amine	DRD2/GRIN2A/RGS9/CNR2	0.002667459	0.023140058

GO:0022602	Ovulation cycle process	MAP2K6/NRIP1/EREG/NOS3	0.00266745<br>9	0.023140058
GO:0035722	Interleukin-12-mediated signaling pathway	IFNG/PLCB1/IL12B/SOD2	0.00266745<br>9	0.023140058
GO:0046638	Positive regulation of alpha-beta T cell differentiation	IFNG/RUNX1/TGFBR2/IL12B	0.00266745<br>9	0.023140058
GO:0060986	Endocrine hormone secretion	GALR1/GNAS/POMC/BMP6	0.00266745<br>9	0.023140058
GO:0005976	Polysaccharide metabolic process	EXT2/ESRRB/TGFB1/POMC/IRS1/ENPP1	0.00268161<br>4	0.02321046
GO:0021953	Central nervous system neuron differentiation	NRP1/KNDC1/DRD2/RORA/CSNK1D/DCC/SHANK3/GABRB1	0.00271712<br>7	0.023276731
GO:0048015	Phosphatidylinositol-mediated signaling	PLCB3/PIK3C2G/HTR2A/TSC2/NF1/PLCB1/IRS1/PDGFC	0.00271712<br>7	0.023276731
GO:0016048	Detection of temperature stimulus	HTR2A/TRPV1/TRPM8	0.00274378<br>3	0.023276731
GO:0031281	Positive regulation of cyclase activity	CACNA1C/NF1/NOS3	0.00274378<br>3	0.023276731
GO:0050951	Sensory perception of temperature stimulus	HTR2A/TRPV1/TRPM8	0.00274378<br>3	0.023276731
GO:0071305	Cellular response to vitamin D	PTN/PENK/TNC	0.00274378<br>3	0.023276731
GO:0086012	Membrane depolarization during cardiac muscle cell action potential	CACNB2/CACNA1C/SCN5A	0.00274378<br>3	0.023276731
GO:1903798	Regulation of production of mirnas involved in gene silencing by mirna	NCOR2/MAP2K1/TGFB1	0.00274378<br>3	0.023276731
GO:2000178	Negative regulation of neural precursor cell proliferation	BDNF/NF1/PTN	0.00274378<br>3	0.023276731
GO:0000271	Polysaccharide biosynthetic process	EXT2/ESRRB/TGFB1/IRS1/ENPP1	0.00279515<br>7	0.023486574
GO:0042310	Vasoconstriction	AVPR1A/HTR2A/CHRM3/DOCK4/ADRA1A	0.00279515<br>7	0.023486574
GO:0061045	Negative regulation of wound healing	PRKG1/SMAD3/AJAP1/FGF2/NOS3	0.00279515<br>7	0.023486574
GO:0001938	Positive regulation of endothelial cell proliferation	NRP1/MIR132/NF1/PRKCA/FGF2/BMP6	0.00280338	0.023486574

GO:1903510	Mucopolysaccharide metabolic process	CHST3/SPOCK2/ACAN/TGFB1/FGF2/SLC10A7	0.00280338	0.023486574
GO:0002793	Positive regulation of peptide secretion	FRMD4A/DRD2/LRP1/IFNG/NLGN2/TGFB1/ABCG1/BMP6	0.002805188	0.023486574
GO:0060562	Epithelial tube morphogenesis	NRP1/EFNB2/COL4A1/MTHFD1/SMAD3/TSC2/TGFB1/TGFBR2/FGF2/NOTCH4/TNC	0.002830849	0.023649895
GO:0042220	Response to cocaine	DRD2/HTR2A/SLC6A3/SDK1	0.00288216	0.02381563
GO:0048701	Embryonic cranial skeleton morphogenesis	MTHFD1/SMAD3/GNAS/TGFBR2	0.00288216	0.02381563
GO:0070169	Positive regulation of biomineral tissue development	SMAD3/TGFB1/BMP6/PTN	0.00288216	0.02381563
GO:0099637	Neurotransmitter receptor transport	EFNB2/SNAP25/CACNG2/MAPK10	0.00288216	0.02381563
GO:0110151	Positive regulation of biomineralization	SMAD3/TGFB1/BMP6/PTN	0.00288216	0.02381563
GO:0007411	Axon guidance	NRP1/BDNF/NCAM1/LRP1/EFNB2/PRKCA/DCC/NOTCH3/SHANK3/GFRA2	0.00288787	0.02381563
GO:0022037	Metencephalon development	KNDC1/RORA/MAP2K1/SCN5A/FOXP2/PTN	0.00292924	0.024053567
GO:0032411	Positive regulation of transporter activity	CACNB2/IFNG/RGS9/CACNG2/SHANK3/PON1	0.00292924	0.024053567
GO:0097485	Neuron projection guidance	NRP1/BDNF/NCAM1/LRP1/EFNB2/PRKCA/DCC/NOTCH3/SHANK3/GFRA2	0.002961304	0.02426501
GO:0043542	Endothelial cell migration	NRP1/EFNB2/MIR132/NF1/PRKCA/TGFB1/DPP4/FGF2/PTN/NOS3	0.003036222	0.02482596
GO:0031623	Receptor internalization	DRD2/LRP1/EFNB2/SNAP25/CACNG2/RAMP1	0.00305928	0.024886523
GO:0061351	Neural precursor cell proliferation	BDNF/DRD2/RORA/NF1/KIF1A/CX3CR1/PTN	0.003067315	0.024886523
GO:0048017	Inositol lipid-mediated signaling	PLCB3/PIK3C2G/HTR2A/TSC2/NF1/PLCB1/IRS1/PDGFC	0.003082801	0.024886523
GO:0021544	Subpallium development	DRD2/SHANK3/FOXP2	0.003107412	0.024886523
GO:0021697	Cerebellar cortex formation	KNDC1/RORA/MAP2K1	0.003107412	0.024886523

GO:0045821	Positive regulation of glycolytic process	IFNG/HTR2A/ESRRB	0.003107412	0.024886523
GO:0051349	Positive regulation of lyase activity	CACNA1C/NF1/NOS3	0.003107412	0.024886523
GO:1902993	Positive regulation of amyloid precursor protein catabolic process	SPON1/IFNG/ABCG1	0.003107412	0.024886523
GO:0035196	Production of mirnas involved in gene silencing by mirna	NCOR2/MAP2K1/SMAD3/TGFB1	0.003108387	0.024886523
GO:0071349	Cellular response to interleukin-12	IFNG/PLCB1/IL12B/SOD2	0.003108387	0.024886523
GO:0050773	Regulation of dendrite development	KNDC1/DCC/SHANK3/KIF1A/SDK1/CUX1/PTN	0.003179444	0.025402502
GO:0016049	Cell growth	NRP1/BDNF/LRP1/AVPR1A/ESR2/SMAD3/TRPV2/DCC/TGFB1/RAPH1/TGFBR2/VGLL4/ENPP1/ADRA1A	0.003211661	0.025606664
GO:0000187	Activation of MAPK activity	MAP2K1/MAP2K6/TGFA/MAPK10/FGF2/GRM1/NRG1	0.003294679	0.026160027
GO:0030902	Hindbrain development	KNDC1/RORA/MAP2K1/CTNNA2/SCN5A/FOXP2/PTN	0.003294679	0.026160027
GO:0022407	Regulation of cell-cell adhesion	PRKG1/RAG1/IFNG/EFNB2/PRKCA/TGFB1/RUNX1/DPP4/TGFBR2/IL12B/HLA-DPA1/NOTCH4/BMP6	0.003302042	0.026164429
GO:0003044	Regulation of systemic arterial blood pressure mediated by a chemical signal	AVPR1A/SOD2/NOS3/ADRA1A	0.003346435	0.02619216
GO:0045981	Positive regulation of nucleotide metabolic process	IFNG/HTR2A/ESRRB/NOS3	0.003346435	0.02619216
GO:0070671	Response to interleukin-12	IFNG/PLCB1/IL12B/SOD2	0.003346435	0.02619216
GO:0071715	Icosanoid transport	ABCC2/DRD2/ABCC4/MAP2K6	0.003346435	0.02619216
GO:1900544	Positive regulation of purine nucleotide metabolic process	IFNG/HTR2A/ESRRB/NOS3	0.003346435	0.02619216
GO:1901571	Fatty acid derivative transport	ABCC2/DRD2/ABCC4/MAP2K6	0.003346435	0.02619216
GO:2000377	Regulation of reactive oxygen species metabolic process	MMP3/IFNG/SMAD3/TRPV1/MIR132/TGFB1/CX3CR1/TGFBR2	0.003485707	0.027057631

GO:0014829	Vascular associated smooth muscle contraction	HTR2A/CHRM3/DOCK4	0.003499256	0.027057631
GO:0048169	Regulation of long-term neuronal synaptic plasticity	DRD2/NF1/SHANK3	0.003499256	0.027057631
GO:0070920	Regulation of production of small RNA involved in gene silencing by RNA	NCOR2/MAP2K1/TGFB1	0.003499256	0.027057631
GO:0097164	Ammonium ion metabolic process	TPH2/HDC/GRIN2A	0.003499256	0.027057631
GO:2000311	Regulation of AMPA receptor activity	NLGN2/CACNG2/SHANK3	0.003499256	0.027057631
GO:0072001	Renal system development	NRP1/BDNF/EFNB2/COL4A1/SMAD3/NF1/NOTCH3/CASP9/FGF2/BMP6	0.003518107	0.027148763
GO:0030168	Platelet activation	PRKG1/PRKCA/GNAS/P2RY12/CLIC1/DGKI/NOS3	0.003534691	0.027167638
GO:0030203	Glycosaminoglycan metabolic process	CHST3/SPOCK2/EXT2/ACAN/TGFB1/FGF2/SLC10A7	0.003534691	0.027167638
GO:0035176	Social behavior	AVPR1A/SHANK3/CX3CR1/ATXN1	0.003596596	0.027533304
GO:1902893	Regulation of pri-mirna transcription by RNA polymerase II	TEAD1/SMAD3/TGFB1/FGF2	0.003596596	0.027533304
GO:0031103	Axon regeneration	LRP1/MAP2K1/PTN/TNC	0.003859157	0.029426071
GO:0060425	Lung morphogenesis	MAP2K1/TGFBR2/FOXP2/TNC	0.003859157	0.029426071
GO:0006352	DNA-templated transcription, initiation	TEAD1/MAML2/ESR2/ESRRB/RORA/NOTCH3/MAML3/POLR1C/NOTCH4	0.003897546	0.029543098
GO:0035640	Exploration behavior	NLGN2/DPP4/PENK	0.003919975	0.029543098
GO:0055083	Monovalent inorganic anion homeostasis	ABCC2/SLC12A5/ENPP1	0.003919975	0.029543098
GO:0060390	Regulation of SMAD protein signal transduction	TGFB1/VEPH1/BMP6	0.003919975	0.029543098
GO:0140058	Neuron projection arborization	NRP1/PHACTR1/PTN	0.003919975	0.029543098

GO:0060541	Respiratory system development	MAP2K1/CYP1A2/TGFBR2/FGF2/FOXP2/PTN/NOS3/TNC	0.003928317	0.029543098
GO:1903708	Positive regulation of hemopoiesis	RAG1/IFNG/PRKCA/TGFB1/GNAS/RUNX1/TGFBR2/IL12B	0.003928317	0.029543098
GO:0042176	Regulation of protein catabolic process	ARNTL/LRP1/IFNG/SMAD3/GRIN2A/WWP2/CSNK1D/FAF1/TNFRSF1B/VGLL4/RNF144B/NRG1	0.003988717	0.029938749
GO:0009636	Response to toxic substance	ABCC2/DRD2/OPRD1/GABRB1/SOD2/AHR/PON1/NOS3/PENK	0.004000798	0.02997089
GO:0032092	Positive regulation of protein binding	NRP1/BDNF/SPON1/LRP1/RAMP1	0.004051791	0.030079619
GO:0045844	Positive regulation of striated muscle tissue development	ARNTL/EFNB2/TGFB1/FGF2/NRG1	0.004051791	0.030079619
GO:0048636	Positive regulation of muscle organ development	ARNTL/EFNB2/TGFB1/FGF2/NRG1	0.004051791	0.030079619
GO:0043467	Regulation of generation of precursor metabolites and energy	IFNG/HTR2A/ESRRB/PRDM16/POMC/IRS1/ENPP1	0.004054448	0.030079619
GO:1903305	Regulation of regulated secretory pathway	CACNB2/DRD2/HTR2A/CACNA1H/CHRM2/DGKI/ADRA1A	0.004054448	0.030079619
GO:0010811	Positive regulation of cell-substrate adhesion	NRP1/RSU1/SPOCK2/SMAD3/UTRN/PTN	0.004093789	0.030254676
GO:0045471	Response to ethanol	DRD2/GRIN2B/GRIN2A/SLC6A3/PENK/TNC	0.004093789	0.030254676
GO:0007632	Visual behavior	RAG1/DRD2/GRIN2A/NF1	0.004134401	0.030321573
GO:0048168	Regulation of neuronal synaptic plasticity	DRD2/NF1/SHANK3/CAMK2A	0.004134401	0.030321573
GO:0051703	Intraspecies interaction between organisms	AVPR1A/SHANK3/CX3CR1/ATXN1	0.004134401	0.030321573
GO:0061614	Pri-mirna transcription by RNA polymerase II	TEAD1/SMAD3/TGFB1/FGF2	0.004134401	0.030321573
GO:0034109	Homotypic cell-cell adhesion	PRKG1/PRKCA/GNAS/P2RY12/CLIC1	0.00425951	0.031002457
GO:0050772	Positive regulation of axonogenesis	NRP1/BDNF/LRP1/MAP2K1/TRPV2	0.00425951	0.031002457
GO:0097756	Negative regulation of blood vessel diameter	AVPR1A/HTR2A/CHRM3/DOCK4/ADRA1A	0.00425951	0.031002457

GO:1901863	Positive regulation of muscle tissue development	ARNTL/EFNB2/TGFB1/FGF2/NRG1	0.00425951	0.031002457
GO:0046942	Carboxylic acid transport	ABCC2/BDNF/DRD2/AVPR1A/ABCC4/TRPV1/NF1/MAP2K6/SNAP25/GRM7/SLC10A7	0.004311058	0.031318325
GO:0043535	Regulation of blood vessel endothelial cell migration	NRP1/MIR132/NF1/PRKCA/TGFB1/FGF2/NOS3	0.004335097	0.031333272
GO:0010632	Regulation of epithelial cell migration	NRP1/IFNG/MIR132/NF1/PRKCA/TGFB1/TGFBR2/FGF2/PTN/NOS3	0.004352915	0.031333272
GO:0006829	Zinc ion transport	SLC39A9/TRPM2/SLC39A8	0.004370188	0.031333272
GO:0045672	Positive regulation of osteoclast differentiation	IFNG/GNAS/IL12B	0.004370188	0.031333272
GO:0048668	Collateral sprouting	BDNF/LRP1/DCC	0.004370188	0.031333272
GO:0051953	Negative regulation of amine transport	DRD2/GRM7/P2RY12	0.004370188	0.031333272
GO:0099590	Neurotransmitter receptor internalization	EFNB2/SNAP25/CACNG2	0.004370188	0.031333272
GO:0045766	Positive regulation of angiogenesis	NRP1/MIR132/PRKCA/RUNX1/CX3CR1/TGFBR2/FGF2/NOS3	0.00441316	0.031358093
GO:0071222	Cellular response to lipopolysaccharide	ABCC2/PRKCA/TGFB1/TNFRSF1B/CX3CR1/IL12B/BMP6/NOS3	0.00441316	0.031358093
GO:0006584	Catecholamine metabolic process	DRD2/HDC/GRIN2A/SLC6A3	0.004422609	0.031358093
GO:0009712	Catechol-containing compound metabolic process	DRD2/HDC/GRIN2A/SLC6A3	0.004422609	0.031358093
GO:0031050	Dsrna processing	NCOR2/MAP2K1/SMAD3/TGFB1	0.004422609	0.031358093
GO:0070918	Production of small RNA involved in gene silencing by RNA	NCOR2/MAP2K1/SMAD3/TGFB1	0.004422609	0.031358093
GO:0045926	Negative regulation of growth	NRP1/TLL2/ESR2/SMAD3/DCC/TGFB1/TGFBR2/VGLL4/ENPP1	0.004435403	0.031390888
GO:0055072	Iron ion homeostasis	IFNG/HMOX2/CP/SLC39A8/BMP6	0.004474717	0.031495126
GO:1904705	Regulation of vascular associated smooth muscle cell proliferation	PRKG1/MMP2/DNMT1/FGF2/SOD2	0.004474717	0.031495126

GO:1990874	Vascular associated smooth muscle cell proliferation	PRKG1/MMP2/DNMT1/FGF2/SOD2	0.004474717	0.031495126
GO:0062012	Regulation of small molecule metabolic process	AVPR1A/IFNG/NCOR2/HTR2A/ESRRB/RORA/TGFB1/ABCG1/POMC/IRS1/BMP6/ENPP1/NOS3	0.004537851	0.0318469
GO:0035303	Regulation of dephosphorylation	DLG2/DRD2/IFNG/SMAD3/SMG6/TGFB1/PHACTR1/PTN	0.00454127	0.0318469
GO:0015849	Organic acid transport	ABCC2/BDNF/DRD2/AVPR1A/ABCC4/TRPV1/NF1/MAP2K6/SNAP25/GRM7/SLC10A7	0.004592739	0.032149174
GO:0001952	Regulation of cell-matrix adhesion	NRP1/LRP1/SMAD3/NF1/AJAP1/UTRN	0.004608445	0.032200462
GO:0002573	Myeloid leukocyte differentiation	IFNG/NF1/PRKCA/TGFB1/GNAS/RUNX1/TGFBR2/IL12B	0.004672221	0.032527802
GO:0006109	Regulation of carbohydrate metabolic process	IFNG/HTR2A/ESRRB/RORA/TGFB1/POMC/IRS1/ENPP1	0.004672221	0.032527802
GO:0046889	Positive regulation of lipid biosynthetic process	AVPR1A/IFNG/HTR2A/ABCG1/BMP6	0.004697557	0.03264505
GO:0002931	Response to ischemia	MAP2K6/CASP9/CX3CR1/CAMK2A	0.004724055	0.032769936
GO:0022011	Myelination in peripheral nervous system	NF1/PRX/NRG1	0.004850483	0.033345986
GO:0032292	Peripheral nervous system axon ensheathment	NF1/PRX/NRG1	0.004850483	0.033345986
GO:0045932	Negative regulation of muscle contraction	PRKG1/KCNMA1/DOCK4	0.004850483	0.033345986
GO:0095500	Acetylcholine receptor signaling pathway	CHRM3/PLCB1/CHRM2	0.004850483	0.033345986
GO:1902932	Positive regulation of alcohol biosynthetic process	IFNG/ABCG1/BMP6	0.004850483	0.033345986
GO:0030278	Regulation of ossification	SMAD3/TGFB1/GNAS/ANKH/CLIC1/BMP6/ENPP1/PTN	0.00494281	0.033859571
GO:1901215	Negative regulation of neuron death	NRP1/BDNF/LRP1/GABRB3/TNFRSF1B/CX3CR1/SOD2/OXR1	0.00494281	0.033859571
GO:0017015	Regulation of transforming growth factor beta receptor signaling pathway	HTRA1/SMAD3/TGFB1/PRDM16/TGFBR2/VEPH1	0.00497705	0.034033454

GO:0086002	Cardiac muscle cell action potential involved in contraction	CACNB2/CACNA1C/KCND3/SCN5A	0.005039011	0.034395951
GO:0050870	Positive regulation of T cell activation	RAG1/IFNG/EFNB2/RUNX1/DPP4/TGFBR2/IL12B/HLA-DPA1	0.005225254	0.035603994
GO:0034614	Cellular response to reactive oxygen species	MMP3/STAT6/MMP2/TRPM2/SOD2/NOS3/SIGMAR1	0.00526562	0.035812915
GO:0062197	Cellular response to chemical stress	ARNTL/MMP3/STAT6/MMP2/TRPM2/MAPK10/SOD2/NOS3/PENK/OXR1/SIGMAR1	0.005307064	0.035812915
GO:0010769	Regulation of cell morphogenesis involved in differentiation	NRP1/KNDC1/BDNF/LRP1/MAP2K1/TRPV2/DCC/SHANK3/KIF1A/CUX1	0.005336867	0.035812915
GO:0034599	Cellular response to oxidative stress	ARNTL/MMP3/STAT6/MMP2/TRPM2/SOD2/NOS3/PENK/OXR1/SIGMAR1	0.005336867	0.035812915
GO:0032373	Positive regulation of sterol transport	LRP1/ABCG1/PON1	0.005361408	0.035812915
GO:0032376	Positive regulation of cholesterol transport	LRP1/ABCG1/PON1	0.005361408	0.035812915
GO:0048679	Regulation of axon regeneration	LRP1/MAP2K1/PTN	0.005361408	0.035812915
GO:2000727	Positive regulation of cardiac muscle cell differentiation	EFNB2/TGFB1/NRG1	0.005361408	0.035812915
GO:1903844	Regulation of cellular response to transforming growth factor beta stimulus	HTRA1/SMAD3/TGFB1/PRDM16/TGFBR2/VEPH1	0.005366867	0.035812915
GO:0045747	Positive regulation of Notch signaling pathway	MAML2/MAML3/NOTCH4/NOS3	0.005367744	0.035812915
GO:0097120	Receptor localization to synapse	DLG2/SNAP25/CACNG2/MAPK10	0.005367744	0.035812915
GO:0098900	Regulation of action potential	CACNA1C/CNR2/SCN5A/ADRA1A	0.005367744	0.035812915
GO:0034103	Regulation of tissue remodeling	NF1/PRKCA/MC4R/TGFB1/IL12B	0.005413312	0.03586786
GO:0046849	Bone remodeling	NF1/PRKCA/MC4R/TGFB1/PTN	0.005413312	0.03586786
GO:0051952	Regulation of amine transport	DRD2/AVPR1A/HTR2A/GRM7/P2RY12	0.005413312	0.03586786

GO:2000177	Regulation of neural precursor cell proliferation	BDNF/DRD2/NF1/CX3CR1/PTN	0.005413312	0.03586786
GO:0030856	Regulation of epithelial cell differentiation	ARNTL/IFNG/AJAP1/RUNX1/FGF2/NOTCH4/BMP6	0.005434232	0.035944496
GO:0051651	Maintenance of location in cell	DRD2/CACNA1C/HTR2A/TRPV1/TRPM2/FGF2/SYNE1/NPSR1	0.005519885	0.036448313
GO:0006022	Aminoglycan metabolic process	CHST3/SPOCK2/EXT2/ACAN/TGFB1/FGF2/SLC10A7	0.005606862	0.036959125
GO:0045582	Positive regulation of T cell differentiation	RAG1/IFNG/RUNX1/TGFBR2/IL12B	0.005668119	0.037298938
GO:0043551	Regulation of phosphatidylinositol 3-kinase activity	TGFB1/IRS1/P2RY12/FGF2	0.005710514	0.037513686
GO:0046887	Positive regulation of hormone secretion	DRD2/LRP1/CYP19A1/NLGN2/GALR1/BMP6	0.005778594	0.037799755
GO:0021543	Pallium development	LRP1/NF1/PLCB1/P2RY12/TACC3/PHACTR1/FOXP2	0.005783569	0.037799755
GO:0099173	Postsynapse organization	NRP1/DLG2/GRIN2B/NLGN2/SHANK3/KIF1A/PTN	0.005783569	0.037799755
GO:0005979	Regulation of glycogen biosynthetic process	ESRRB/IRS1/ENPP1	0.005903479	0.03812953
GO:0010962	Regulation of glucan biosynthetic process	ESRRB/IRS1/ENPP1	0.005903479	0.03812953
GO:0048265	Response to pain	LPAR5/TRPV1/TACR1	0.005903479	0.03812953
GO:0070168	Negative regulation of biomineral tissue development	TGFB1/ENPP1/NOS3	0.005903479	0.03812953
GO:0110150	Negative regulation of biomineralization	TGFB1/ENPP1/NOS3	0.005903479	0.03812953
GO:1905144	Response to acetylcholine	CHRM3/PLCB1/CHRM2	0.005903479	0.03812953
GO:1905145	Cellular response to acetylcholine	CHRM3/PLCB1/CHRM2	0.005903479	0.03812953
GO:0061097	Regulation of protein tyrosine kinase activity	BDNF/TGFA/EREG/NRG1/ADRA1A	0.005931272	0.0381807
GO:1903035	Negative regulation of response to wounding	PRKG1/SMAD3/AJAP1/FGF2/NOS3	0.005931272	0.0381807

GO:0010595	Positive regulation of endothelial cell migration	NRP1/MIR132/PRKCA/TGFB1/FGF2/NOS3	0.005992891	0.038512846
GO:0031102	Neuron projection regeneration	LRP1/MAP2K1/PTN/TNC	0.00606758	0.038798188
GO:0031663	Lipopolysaccharide-mediated signaling pathway	PRKCA/TGFB1/BMP6/NOS3	0.00606758	0.038798188
GO:0042306	Regulation of protein import into nucleus	IFNG/SMAD3/NF1/TGFB1	0.00606758	0.038798188
GO:0050680	Negative regulation of epithelial cell proliferation	EFNB2/MIR132/NF1/TGFB1/EREG/IL12B/PTN	0.006149449	0.039256367
GO:0010660	Regulation of muscle cell apoptotic process	IFNG/DNMT1/CAMK2A/IL12B/SOD2	0.006202913	0.039530259
GO:0090101	Negative regulation of transmembrane receptor protein serine/threonine kinase signaling pathway	HTRA1/SMAD3/TGFB1/PRDM16/TGFBR2/VEPH1	0.006212926	0.039530259
GO:0071241	Cellular response to inorganic substance	DLG2/MMP3/TPH2/CYP1A2/CACNA1H/TRPM2/SCN5A/BMP6	0.006311963	0.039556619
GO:0031348	Negative regulation of defense response	HTRA1/DRD2/CYP19A1/RORA/SMAD3/CNR2/TNFRSF1B/IL1R2/IL12B	0.006401776	0.039556619
GO:0002676	Regulation of chronic inflammatory response	CYP19A1/LTA	0.006402346	0.039556619
GO:0006547	Histidine metabolic process	MTHFD1/HDC	0.006402346	0.039556619
GO:0006930	Substrate-dependent cell migration, cell extension	NRP1/P2RY12	0.006402346	0.039556619
GO:0014877	Response to muscle inactivity involved in regulation of muscle adaptation	SCN5A/UTRN	0.006402346	0.039556619
GO:0014894	Response to denervation involved in regulation of muscle adaptation	SCN5A/UTRN	0.006402346	0.039556619
GO:0032341	Aldosterone metabolic process	CACNA1H/BMP6	0.006402346	0.039556619
GO:0032342	Aldosterone biosynthetic process	CACNA1H/BMP6	0.006402346	0.039556619
GO:0035933	Glucocorticoid secretion	GALR1/POMC	0.006402346	0.039556619

GO:0042428	Serotonin metabolic process	TPH2/GRIN2A	0.006402346	0.039556619
GO:0044557	Relaxation of smooth muscle	PRKG1/KCNMA1	0.006402346	0.039556619
GO:0051967	Negative regulation of synaptic transmission, glutamatergic	DRD2/HTR2A	0.006402346	0.039556619
GO:0060502	Epithelial cell proliferation involved in lung morphogenesis	MAP2K1/FOXP2	0.006402346	0.039556619
GO:0098598	Learned vocalization behavior or vocal learning	SHANK3/FOXP2	0.006402346	0.039556619
GO:1901203	Positive regulation of extracellular matrix assembly	SMAD3/TGFB1	0.006402346	0.039556619
GO:2000048	Negative regulation of cell-cell adhesion mediated by cadherin	NOTCH4/BMP6	0.006402346	0.039556619
GO:2000169	Regulation of peptidyl-cysteine S-nitrosylation	MIR132/OXR1	0.006402346	0.039556619
GO:0042093	T-helper cell differentiation	STAT6/RORA/IL18R1/IL12B	0.006439194	0.039572277
GO:0014044	Schwann cell development	NF1/PRX/NRG1	0.006477178	0.039572277
GO:0045940	Positive regulation of steroid metabolic process	IFNG/ABCG1/BMP6	0.006477178	0.039572277
GO:0017157	Regulation of exocytosis	CACNB2/DRD2/IFNG/HTR2A/CACNA1H/CHRM2/DGKI/ADRA1A	0.006480229	0.039572277
GO:0071219	Cellular response to molecule of bacterial origin	ABCC2/PRKCA/TGFB1/TNFRSF1B/CX3CR1/IL12B/BMP6/NOS3	0.006480229	0.039572277
GO:1903725	Regulation of phospholipid metabolic process	HTR2A/TGFB1/IRS1/P2RY12/FGF2	0.006483181	0.039572277
GO:0009416	Response to light stimulus	RAG1/DRD2/MTA1/TIPIN/GRIN2A/NF1/CASP9/MAPK10/IL12B/PTN	0.006487258	0.039572277
GO:0051961	Negative regulation of nervous system development	NRP1/BDNF/LRP1/EFNB2/TRPV1/NF1/DCC/NOTCH3/PTN/NRG1	0.006487258	0.039572277
GO:0003170	Heart valve development	TGFB1/TNFRSF1B/TGFBR2/NOS3	0.006825603	0.041570196
GO:0045732	Positive regulation of protein catabolic process	LRP1/IFNG/WWP2/CSNK1D/FAF1/TNFRSF1B/VGLL4/RNF144B	0.007005485	0.042464567

GO:0001667	Ameboidal-type cell migration	NRP1/IFNG/EFNB2/MIR132/NF1/PRKCA/TGFB1/DPP4/TGFBR2/P2RY12/FGF2/PTN/NOS3	0.007007548	0.042464567
GO:1901216	Positive regulation of neuron death	GRIN2B/IFNG/EFNB2/NF1/CASP9	0.00707015	0.042464567
GO:0003176	Aortic valve development	TGFB1/TNFRSF1B/NOS3	0.007082953	0.042464567
GO:0038128	ERBB2 signaling pathway	PRKCA/EREG/NRG1	0.007082953	0.042464567
GO:0051968	Positive regulation of synaptic transmission, glutamatergic	NLGN2/CACNG2/SHANK3	0.007082953	0.042464567
GO:0070570	Regulation of neuron projection regeneration	LRP1/MAP2K1/PTN	0.007082953	0.042464567
GO:0071295	Cellular response to vitamin	PTN/PENK/TNC	0.007082953	0.042464567
GO:0086019	Cell-cell signaling involved in cardiac conduction	CACNB2/CACNA1C/SCN5A	0.007082953	0.042464567
GO:0097106	Postsynaptic density organization	DLG2/NLGN2/SHANK3	0.007082953	0.042464567
GO:0030534	Adult behavior	DRD2/HTR2A/OPRD1/SHANK3/SDK1/OXR1	0.007152177	0.042812798
GO:0044706	Multi-multicellular organism process	ABCC2/ARNTL/AVPR1A/MMP2/TGFBR2/CLIC5/DDO/PTN	0.007187531	0.042957514
GO:0055025	Positive regulation of cardiac muscle tissue development	EFNB2/TGFB1/FGF2/NRG1	0.00722705	0.042960447
GO:0140115	Export across plasma membrane	ABCC2/ABCC4/RGS9/KCND3	0.00722705	0.042960447
GO:1904589	Regulation of protein import	IFNG/SMAD3/NF1/TGFB1	0.00722705	0.042960447
GO:0042110	T cell activation	RAG1/STAT6/IFNG/EFNB2/RORA/SMAD3/TNFRSF1B/RUNX1/IL18R1/DPP4/TGFBR2/IL12B/HLA-DPA1	0.007243916	0.042960447
GO:0051051	Negative regulation of transport	FRMD4A/DRD2/HTR2A/WWP2/NF1/TNFRSF1B/IRS1/GRM7/P2RY12/IL12B/ENPP1/NOS3/NRG1	0.007243916	0.042960447
GO:0010657	Muscle cell apoptotic process	IFNG/DNMT1/CAMK2A/IL12B/SOD2	0.007377127	0.043683047
GO:0048705	Skeletal system morphogenesis	FGF6/MTHFD1/SMAD3/ACAN/MMP2/GNAS/TGFBR2/BMP6	0.007562319	0.044586367

GO:0046635	Positive regulation of alpha-beta T cell activation	IFNG/RUNX1/TGFBR2/IL12B	0.007643772	0.044586367
GO:2000514	Regulation of CD4-positive, alpha-beta T cell activation	IFNG/RUNX1/TGFBR2/IL12B	0.007643772	0.044586367
GO:0015837	Amine transport	DRD2/AVPR1A/HTR2A/GRM7/P2RY12	0.007693278	0.044586367
GO:0010039	Response to iron ion	DRD2/SLC6A3/BMP6	0.007721222	0.044586367
GO:0010464	Regulation of mesenchymal cell proliferation	TGFBR2/FOXP2/PTN	0.007721222	0.044586367
GO:0021696	Cerebellar cortex morphogenesis	KNDC1/RORA/MAP2K1	0.007721222	0.044586367
GO:0046627	Negative regulation of insulin receptor signaling pathway	TSC2/IRS1/ENPP1	0.007721222	0.044586367
GO:0120255	Olefinic compound biosynthetic process	CYP19A1/CACNA1H/BMP6	0.007721222	0.044586367
GO:1902003	Regulation of amyloid-beta formation	SPON1/IFNG/ABCG1	0.007721222	0.044586367
GO:0050807	Regulation of synapse organization	BDNF/DRD2/GRIN2B/NLGN2/SHANK3/CTNNA2/KIF1A/PTN	0.007755146	0.044586367
GO:0014870	Response to muscle inactivity	SCN5A/UTRN	0.007761717	0.044586367
GO:0035865	Cellular response to potassium ion	DLG2/CACNA1H	0.007761717	0.044586367
GO:0048642	Negative regulation of skeletal muscle tissue development	TLL2/TGFB1	0.007761717	0.044586367
GO:0048672	Positive regulation of collateral sprouting	BDNF/LRP1	0.007761717	0.044586367
GO:0048680	Positive regulation of axon regeneration	LRP1/PTN	0.007761717	0.044586367
GO:0060439	Trachea morphogenesis	MAP2K1/TGFBR2	0.007761717	0.044586367
GO:0061299	Retina vasculature morphogenesis in camera-type eye	NRP1/COL4A1	0.007761717	0.044586367
GO:0098917	Retrograde trans-synaptic signaling	BDNF/PLCB1	0.007761717	0.044586367

GO:1903800	Positive regulation of production of mirnas involved in gene silencing by mirna	MAP2K1/TGFB1	0.007761717	0.044586367
GO:0045165	Cell fate commitment	NRP1/STAT6/RORA/NOTCH3/MYT1L/FGF2/IL12B/NOTCH4/NRG1	0.007868185	0.045130502
GO:0060048	Cardiac muscle contraction	CACNB2/CACNA1C/MAP2K6/KCND3/SCN5A/ADRA1A	0.00792109	0.045366243
GO:0001936	Regulation of endothelial cell proliferation	NRP1/MIR132/NF1/PRKCA/FGF2/IL12B/BMP6	0.008013178	0.045825359
GO:0046637	Regulation of alpha-beta T cell differentiation	IFNG/RUNX1/TGFBR2/IL12B	0.008076001	0.046116008
GO:0001649	Osteoblast differentiation	SMAD3/NF1/MRC2/GNAS/CLIC1/BMP6/PENK/TNC	0.008151879	0.046411367
GO:0048738	Cardiac muscle tissue development	FGF3/EFNB2/TGFB1/TGFBR2/VGLL4/FGF2/NRG1/ADRA1A	0.008151879	0.046411367
GO:0044264	Cellular polysaccharide metabolic process	EXT2/ESRRB/POMC/IRS1/ENPP1	0.008353639	0.047429245
GO:0030431	Sleep	DRD2/HTR2A/GRIN2A	0.008392372	0.047429245
GO:0048854	Brain morphogenesis	NF1/SHANK3/CTNNA2	0.008392372	0.047429245
GO:0098664	G protein-coupled serotonin receptor signaling pathway	HTR2A/CHRM3/CHRM2	0.008392372	0.047429245
GO:0099084	Postsynaptic specialization organization	DLG2/NLGN2/SHANK3	0.008392372	0.047429245
GO:0032729	Positive regulation of interferon-gamma production	IL18R1/IL12B/HLA-DPA1/LTA	0.008523964	0.048102196
GO:0016054	Organic acid catabolic process	HDC/TGFB1/FAAH/CBS/IRS1/FGF2/DDO/PON1/NOS3	0.008596671	0.04832614
GO:0046395	Carboxylic acid catabolic process	HDC/TGFB1/FAAH/CBS/IRS1/FGF2/DDO/PON1/NOS3	0.008596671	0.04832614
GO:0006066	Alcohol metabolic process	PLCB3/IFNG/SPTLC2/CYP1A2/CACNA1H/PLCB1/ABCG1/GPD2/FGF2/BMP6/PON1	0.008601374	0.04832614
GO:0018209	Peptidyl-serine modification	SPOCK2/BDNF/IFNG/MIR132/PRKCA/CSNK1D/TGFB1/OPRD1/TGFBR2/CAMK2A	0.008649971	0.048528233

GO:0060840	Artery development	NRP1/LRP1/EFNB2/NF1/NOTCH3	0.008698114	0.048656265
GO:0060996	Dendritic spine development	SLC12A5/SHANK3/KIF1A/CAMK2A/SDK1	0.008698114	0.048656265
GO:0001822	Kidney development	NRP1/BDNF/EFNB2/SMAD3/NF1/NOTCH3/CASP9/FGF2/BMP6	0.00878655	0.049079524
