# Supplementary material for: Impaired Molecular Mechanisms Contributing to Chronic Pain in Patients with Hidradenitis Suppurativa: Exploring Potential Biomarkers and Therapeutic Targets
Source: Int J Mol Sci. 2025 Jan 25;26(3):1039. doi: 10.3390/ijms26031039 (PMC11817842; doi:10.3390/ijms26031039)
Supplement: Supplementary file 1 [file ijms-26-01039-s001.zip › Supplementary Table S5.pdf]

Table S5. Comprehensive List of Gene Ontology (GO) Terms for Cellular Component (CC)

ID	Description	Gene ID	p-value	p. adjust
GO:0045211	Postsynaptic membrane	NRP1/KCNMA1/DLG2/DRD2/GRIN2B/CACNA1C/HTR2A/EFNB2/GABRB3/GRIN2A/NLGN2/TRPV1/RGS9/DCC/KCND3/OPRD1/CHRM3/CACNG2/SHANK3/CTNNA2/GRM7/GABRB1/SLC6A3/GABBR1/UTRN/GRM1/SYNE1/KCND2/CHRM2/DGKI/ADRA1A/SIGMAR1	5.11952E-22	1.68432E-19
GO:0097060	Synaptic membrane	NRP1/KCNMA1/DLG2/DRD2/GRIN2B/CACNA1C/HTR2A/EFNB2/GABRB3/GRIN2A/NLGN2/TRPV1/RGS9/DCC/KCND3/OPRD1/CHRM3/SNAP25/CACNG2/SHANK3/CTNNA2/GRM7/GABRB1/SLC6A3/GABBR1/UTRN/GRM1/SYNE1/KCND2/CHRM2/DGKI/ADRA1A/SIGMAR1	1.03908E-18	1.70929E-16
GO:0034702	Ion channel complex	CACNB2/KCNMA1/DLG2/GRIN2B/SCNN1A/CACNA1C/SCN8A/GABRB3/GRIN2A/CACNA1H/KCNAB3/KCND3/SNAP25/CACNG2/SHANK3/SCN3A/CACNA2D3/SCN11A/SCN5A/GABRB1/CLIC5/CLIC1/KCNQ5/KCND2/KCNB2	3.31947E-14	3.64035E-12
GO:1902495	Transmembrane transporter complex	CACNB2/KCNMA1/DLG2/GRIN2B/SCNN1A/CACNA1C/SCN8A/GABRB3/GRIN2A/CACNA1H/KCNAB3/KCND3/SNAP25/CACNG2/SHANK3/SCN3A/CACNA2D3/SCN11A/SCN5A/GABRB1/CLIC5/CLIC1/KCNQ5/KCND2/KCNB2	1.78983E-13	1.47214E-11
GO:1990351	Transporter complex	CACNB2/KCNMA1/DLG2/GRIN2B/SCNN1A/CACNA1C/SCN8A/GABRB3/GRIN2A/CACNA1H/KCNAB3/KCND3/SNAP25/CACNG2/SHANK3/SCN3A/CACNA2D3/SCN11A/SCN5A/GABRB1/CLIC5/CLIC1/KCNQ5/KCND2/KCNB2	3.11027E-13	2.04656E-11
GO:0099634	Postsynaptic specialization membrane	DLG2/GRIN2B/CACNA1C/EFNB2/GRIN2A/NLGN2/RGS9/DCC/KCND3/OPRD1/CHRM3/CACNG2/GRM1/KCND2/DGKI/SIGMAR1	1.01189E-12	5.54856E-11
GO:0034703	Cation channel complex	CACNB2/KCNMA1/DLG2/GRIN2B/SCNN1A/CACNA1C/SCN8A/GRIN2A/CACNA1H/KCNAB3/KCND3/SNAP25/CACNG2/SCN3A/CACNA2D3/SCN11A/SCN5A/KCNQ5/KCND2/KCNB2	3.16676E-12	1.43927E-10
GO:0098984	Neuron to neuron synapse	DLG2/DRD2/GRIN2B/CACNA1C/EFNB2/GRIN2A/TSC2/NLGN2/RGS9/DCC/KCND3/OPRD1/CHRM3/CACNG2/SYN3/SHANK3/CTNNA2/GRM7/CAMK2A/GRM1/KCND2/CHRM2/DGKI/PENK/SIGMAR1	3.49974E-12	1.43927E-10
GO:0099055	Integral component of postsynaptic membrane	NRP1/DRD2/HTR2A/EFNB2/GRIN2A/NLGN2/DCC/KCND3/OPRD1/CHRM3/CACNG2/SLC6A3/KCND2/CHRM2/ADRA1A	1.20754E-11	4.41422E-10
GO:0098936	Intrinsic component of postsynaptic membrane	NRP1/DRD2/HTR2A/EFNB2/GRIN2A/NLGN2/DCC/KCND3/OPRD1/CHRM3/CACNG2/SLC6A3/KCND2/CHRM2/ADRA1A	2.23178E-11	7.34255E-10
GO:0032279	Asymmetric synapse	DLG2/DRD2/GRIN2B/CACNA1C/EFNB2/GRIN2A/TSC2/RGS9/DCC/KCND3/OPRD1/CHRM3/CACNG2/SYN3/SHANK3/CTNNA2/GRM7/CAMK2A/GRM1/KCND2/CHRM2/DGKI/SIGMAR1	3.49228E-11	1.04451E-09
GO:0098839	Postsynaptic density membrane	DLG2/GRIN2B/CACNA1C/EFNB2/GRIN2A/RGS9/DCC/OPRD1/CHRM3/CACNG2/GRM1/DGKI/SIGMAR1	8.36304E-11	2.29287E-09

GO:0099572	Postsynaptic specialization	DLG2/DRD2/GRIN2B/CACNA1C/EFNB2/GRIN2A/TSC2/NLGN2/RGS9/DCC/KCND3/OPRD1/CHRM3/CACNG2/SYN3/SHANK3/CTNNA2/CAMK2A/GRM1/KCND2/DGKI/SIGMAR1	5.87556E-10	1.48582E-08
GO:0099699	Integral component of synaptic membrane	NRP1/DRD2/HTR2A/EFNB2/GRIN2A/NLGN2/DCC/KCND3/OPRD1/CHRM3/CACNG2/SLC6A3/KCND2/CHRM2/ADRA1A	6.32265E-10	1.48582E-08
GO:0014069	Postsynaptic density	DLG2/DRD2/GRIN2B/CACNA1C/EFNB2/GRIN2A/TSC2/RGS9/DCC/KCND3/OPRD1/CHRM3/CACNG2/SYN3/SHANK3/CTNNA2/CAMK2A/GRM1/KCND2/DGKI/SIGMAR1	9.85599E-10	2.16175E-08
GO:0098982	GABA-ergic synapse	DRD2/GABRB3/ACAN/NLGN2/KCND3/PLCB1/GABRB1/GABBR1/KCND2/NRG1/ADRA1A	1.40041E-09	2.87959E-08
GO:0099240	Intrinsic component of synaptic membrane	NRP1/DRD2/HTR2A/EFNB2/GRIN2A/NLGN2/DCC/KCND3/OPRD1/CHRM3/CACNG2/SLC6A3/KCND2/CHRM2/ADRA1A	1.80591E-09	3.49497E-08
GO:0042734	Presynaptic membrane	DRD2/HTR2A/EFNB2/GRIN2A/NLGN2/OPRD1/CHRM3/SNAP25/CTNNA2/SLC6A3/GABBR1/CHRM2/DGKI/ADRA1A	6.94873E-09	1.27007E-07
GO:0098978	Glutamatergic synapse	NRP1/DLG2/DRD2/HTR2A/EFNB2/ACAN/GRIN2A/RGS9/CHRM3/SNAP25/PLCB1/CACNG2/SYN3/MAPK10/GRM1/KCND2/CHRM2/DGKI/NRG1/ADRA1A	1.83993E-08	3.18599E-07
GO:0099060	Integral component of postsynaptic specialization membrane	EFNB2/GRIN2A/NLGN2/DCC/KCND3/OPRD1/CHRM3/CACNG2/KCND2	2.69686E-07	4.43633E-06
GO:0098948	Intrinsic component of postsynaptic specialization membrane	EFNB2/GRIN2A/NLGN2/DCC/KCND3/OPRD1/CHRM3/CACNG2/KCND2	3.81425E-07	5.97566E-06
GO:0043204	Perikaryon	KNDC1/DLG2/DRD2/CACNA1C/CNR2/ASTN1/TRPM2/MAPK10/PDE10A/KCND2/PENK/KCNB2	4.58194E-07	6.85209E-06
GO:0034706	Sodium channel complex	SCNN1A/SCN8A/CACNA1H/SCN3A/SCN11A/SCN5A	5.58397E-07	7.98751E-06
GO:0005901	Caveola	KCNMA1/HTR2A/IRS1/SCN5A/TGFBR2/P2RY12/SLC6A3/NOS3/ADRA1A	6.57439E-07	9.0124E-06
GO:0044853	Plasma membrane raft	KCNMA1/HTR2A/IRS1/SCN5A/TGFBR2/P2RY12/SLC6A3/KCND2/NOS3/ADRA1A	1.17728E-06	1.54931E-05
GO:0001518	Voltage-gated sodium channel complex	SCN8A/CACNA1H/SCN3A/SCN11A/SCN5A	1.37602E-06	1.7412E-05
GO:0099056	Integral component of presynaptic membrane	DRD2/HTR2A/EFNB2/OPRD1/CHRM3/SLC6A3/CHRM2/ADRA1A	3.1263E-06	3.80945E-05
GO:0098889	Intrinsic component of presynaptic membrane	DRD2/HTR2A/EFNB2/OPRD1/CHRM3/SLC6A3/CHRM2/ADRA1A	7.45093E-06	8.75484E-05
GO:0008076	Voltage-gated potassium channel complex	KCNMA1/DLG2/KCNAB3/KCND3/SNAP25/KCNQ5/KCND2/KCNB2	1.15275E-05	0.000130777
GO:0045121	Membrane raft	KCNMA1/STAT6/HTR2A/OPRD1/TNFRSF1B/DPP4/IRS1/TRPM8/SCN5A/TGFBR2/P2RY12/SLC6A3/KCND2/NOS3/ADRA1A	1.25257E-05	0.000137365
GO:0098857	Membrane microdomain	KCNMA1/STAT6/HTR2A/OPRD1/TNFRSF1B/DPP4/IRS1/TRPM8/SCN5A/TGFBR2/P2RY12/SLC6A3/KCND2/NOS3/ADRA1A	1.29826E-05	0.000137783

GO:0098691	Dopaminergic synapse	DRD2/NLGN2/SLC6A3/ADRA1A	1.37916E-05	0.000141795
GO:0098589	Membrane region	KCNMA1/STAT6/HTR2A/OPRD1/TNFRSF1B/DPP4/IRS1/TRPM8/SCN5A/TGFBR2/P2RY12/SLC6A3/KCND2/NOS3/ADRA1A	2.04222E-05	0.000203603
GO:0034705	Potassium channel complex	KCNMA1/DLG2/KCNAB3/KCND3/SNAP25/KCNQ5/KCND2/KCNB2	2.18856E-05	0.000211775
GO:0044298	Cell body membrane	CX3CR1/P2RY12/SLC6A3/KCND2/KCNB2	3.28973E-05	0.000309234
GO:0099061	Integral component of postsynaptic density membrane	EFNB2/GRIN2A/DCC/OPRD1/CHRM3/CACNG2	3.39158E-05	0.000309952
GO:0099146	Intrinsic component of postsynaptic density membrane	EFNB2/GRIN2A/DCC/OPRD1/CHRM3/CACNG2	4.71875E-05	0.000419586
GO:0089717	Spanning component of membrane	SLC24A4/NLGN2/AJAP1/SLC24A3	7.06057E-05	0.000611297
GO:0005891	Voltage-gated calcium channel complex	CACNB2/CACNA1C/CACNA1H/CACNG2/CACNA2D3	0.000165582	0.00139683
GO:0008021	Synaptic vesicle	BDNF/DRD2/GRIN2A/OPRD1/SNAP25/SYN3/KIF1A/ICA1/DGKI/PENK	0.000208092	0.001711553
GO:0008328	Ionotropic glutamate receptor complex	DLG2/GRIN2B/GRIN2A/CACNG2/SHANK3	0.00030922	0.002481302
GO:0032809	Neuronal cell body membrane	CX3CR1/SLC6A3/KCND2/KCNB2	0.000342378	0.002606187
GO:0032280	Symmetric synapse	NLGN2/CHRM2/PENK	0.000356469	0.002606187
GO:0044214	Spanning component of plasma membrane	SLC24A4/AJAP1/SLC24A3	0.000356469	0.002606187
GO:0098992	Neuronal dense core vesicle	OPRD1/KIF1A/PENK	0.000356469	0.002606187
GO:0098878	Neurotransmitter receptor complex	DLG2/GRIN2B/GRIN2A/CACNG2/SHANK3	0.000373513	0.002671429
GO:0070382	Exocytic vesicle	BDNF/DRD2/GRIN2A/OPRD1/SNAP25/SYN3/KIF1A/ICA1/DGKI/PENK	0.000407142	0.002807425
GO:0150034	Distal axon	NRP1/DRD2/LRP1/TRPV2/OPRD1/CHRM3/SNAP25/UTRN/CHRM2/DGKI/PENK/SIGMAR1	0.000409594	0.002807425
GO:0098685	Schaffer collateral - CA1 synapse	EFNB2/DCC/CACNG2/GABBR1/GRM1/DGKI	0.000549174	0.00368731
GO:0016323	Basolateral plasma membrane	DLG2/LRP1/ABCC4/AJAP1/CHRM3/CTNNA2/TGFA/P2RY12/SLC39A8/ENPP1	0.000870837	0.005730105
GO:0042383	Sarcolemma	CACNB2/FGF6/CACNA1C/KCND3/SCN5A/UTRN/ADRA1A	0.001307561	0.008305985
GO:0034704	Calcium channel complex	CACNB2/CACNA1C/CACNA1H/CACNG2/CACNA2D3	0.0013128	0.008305985
GO:0043197	Dendritic spine	DRD2/GRIN2A/KCND3/OPRD1/SHANK3/CAMK2A/KCND2/DGKI	0.001353769	0.008403584

GO:0044309	Neuron spine	DRD2/GRIN2A/KCND3/OPRD1/SHANK3/CAMK2A/KCND2/DGKI	0.00145515 8	0.008865687
GO:0034707	Chloride channel complex	GABRB3/GABRB1/CLIC5/CLIC1	0.00312789 9	0.018710522
GO:0030315	T-tubule	CACNB2/CACNA1C/SCN5A/ADRA1A	0.00413670 1	0.023925562
GO:0031045	Dense core granule	OPRD1/KIF1A/PENK	0.00414515 8	0.023925562
GO:0098802	Plasma membrane signaling receptor complex	DLG2/GRIN2B/GRIN2A/CACNG2/SHANK3/IL18R1/IRS1/RAMP1/GABBR1/ GRM1	0.00438486 4	0.02474959
GO:0030133	Transport vesicle	BDNF/DRD2/GRIN2A/OPRD1/SNAP25/SYN3/TGFA/KIF1A/HLA-DPA1/ICA1/DGKI/PENK	0.00443837 6	0.02474959
GO:0035579	Specific granule membrane	HMOX2/SLC44A2/TNFRSF1B/SNAP25/TRPM2	0.00500364 8	0.027436669
GO:0043679	Axon terminus	DRD2/OPRD1/CHRM3/CHRM2/DGKI/PENK	0.00509089 3	0.02745744
GO:0017146	NMDA selective glutamate receptor complex	GRIN2B/GRIN2A	0.00748234 5	0.0397047
GO:1990454	L-type voltage-gated calcium channel complex	CACNB2/CACNA1C	0.00890752	0.046517047
GO:0044306	Neuron projection terminus	DRD2/OPRD1/CHRM3/CHRM2/DGKI/PENK	0.00970703 6	0.049900232
