# Supplementary material for: Impaired Molecular Mechanisms Contributing to Chronic Pain in Patients with Hidradenitis Suppurativa: Exploring Potential Biomarkers and Therapeutic Targets
Source: Int J Mol Sci. 2025 Jan 25;26(3):1039. doi: 10.3390/ijms26031039 (PMC11817842; doi:10.3390/ijms26031039)
Supplement: Supplementary file 1 [file ijms-26-01039-s001.zip › Supplementary Table S6.pdf]

Supplementary Table S6. Comprehensive List of Gene Ontology (GO) Terms for Molecular Function (MF)

ID	Description	Gene ID	p-value	p. adjust
GO:0022836	Gated channel activity	CACNB2/KCNMA1/GRIN2B/SCNN1A/CACNA1C/SCN8A/GABRB3/GRIN2A/CACNA1H/KCNAB3/TRPV1/KCND3/CLCN6/SNAP25/TRPM2/CACNG2/SCN3A/TRPM8/CACNA2D3/SCN11A/SCN5A/GABRB1/CLIC5/CLIC1/KCNQ5/KCND2/KCNB2	5.161E-14	1.4668E-11
GO:0046873	Metal ion transmembrane transporter activity	CACNB2/KCNMA1/GRIN2B/SCNN1A/CACNA1C/SCN8A/SLC39A9/SLC24A4/GRIN2A/CACNA1H/ATP2C2/TRPV2/KCNAB3/TRPV1/KCND3/SLC24A3/SNAP25/SLC12A5/TRPM2/CACNG2/SCN3A/TRPM8/CACNA2D3/SCN11A/SCN5A/SLC39A8/SLC6A3/KCNQ5/KCND2/KCNB2	6.4403E-14	1.4668E-11
GO:0005216	Ion channel activity	CACNB2/KCNMA1/GRIN2B/SCNN1A/CACNA1C/SCN8A/SLC24A4/GABRB3/GRIN2A/CACNA1H/TRPV2/KCNAB3/TRPV1/KCND3/CLCN6/SLC24A3/SNAP25/TRPM2/CACNG2/SCN3A/TRPM8/CACNA2D3/SCN11A/SCN5A/GABRB1/CLIC5/CLIC1/KCNQ5/KCND2/KCNB2	7.7334E-14	1.4668E-11
GO:0005244	Voltage-gated ion channel activity	CACNB2/KCNMA1/GRIN2B/CACNA1C/SCN8A/GRIN2A/CACNA1H/KCNAB3/KCND3/CLCN6/SNAP25/CACNG2/SCN3A/CACNA2D3/SCN11A/SCN5A/CLIC5/CLIC1/KCNQ5/KCND2/KCNB2	1.505E-13	1.7127E-11
GO:0022832	Voltage-gated channel activity	CACNB2/KCNMA1/GRIN2B/CACNA1C/SCN8A/GRIN2A/CACNA1H/KCNAB3/KCND3/CLCN6/SNAP25/CACNG2/SCN3A/CACNA2D3/SCN11A/SCN5A/CLIC5/CLIC1/KCNQ5/KCND2/KCNB2	1.505E-13	1.7127E-11
GO:0015267	Channel activity	CACNB2/KCNMA1/GRIN2B/SCNN1A/CACNA1C/SCN8A/SLC24A4/GABRB3/GRIN2A/CACNA1H/TRPV2/KCNAB3/TRPV1/KCND3/CLCN6/SLC24A3/SNAP25/TRPM2/CACNG2/SCN3A/TRPM8/CACNA2D3/SCN11A/SCN5A/GABRB1/CLIC5/CLIC1/KCNQ5/KCND2/KCNB2	1.1847E-12	1.0156E-10
GO:0022803	Passive transmembrane transporter activity	CACNB2/KCNMA1/GRIN2B/SCNN1A/CACNA1C/SCN8A/SLC24A4/GABRB3/GRIN2A/CACNA1H/TRPV2/KCNAB3/TRPV1/KCND3/CLCN6/SLC24A3/SNAP25/TRPM2/CACNG2/SCN3A/TRPM8/CACNA2D3/SCN11A/SCN5A/GABRB1/CLIC5/CLIC1/KCNQ5/KCND2/KCNB2	1.2494E-12	1.0156E-10
GO:0005261	Cation channel activity	CACNB2/KCNMA1/GRIN2B/SCNN1A/CACNA1C/SCN8A/SLC24A4/GRIN2A/CACNA1H/TRPV2/KCNAB3/TRPV1/KCND3/SLC24A3/SNAP25/TRPM2/CACNG2/SCN3A/TRPM8/CACNA2D3/SCN11A/SCN5A/KCNQ5/KCND2/KCNB2	2.1408E-12	1.5227E-10

GO:0015085	Calcium ion transmembrane transporter activity	CACNB2/GRIN2B/CACNA1C/SLC24A4/GRIN2A/CACNA1H/ATP2C2/TRPV2/TRPV1/SLC24A3/TRPM2/CACNG2/TRPM8/CACNA2D3	2.4923E-09	1.5757E-07
GO:0072509	Divalent inorganic cation transmembrane transporter activity	CACNB2/GRIN2B/CACNA1C/SLC24A4/GRIN2A/CACNA1H/ATP2C2/TRPV2/TRPV1/SLC24A3/TRPM2/CACNG2/TRPM8/CACNA2D3/SLC39A8	3.5778E-09	2.0358E-07
GO:0022843	Voltage-gated cation channel activity	CACNB2/KCNMA1/GRIN2B/CACNA1C/GRIN2A/CACNA1H/KCNAB3/KCND3/SNAP25/CACNG2/CACNA2D3/KCNQ5/KCND2/KCNB2	4.4293E-09	2.2912E-07
GO:0005262	Calcium channel activity	CACNB2/GRIN2B/CACNA1C/SLC24A4/GRIN2A/CACNA1H/TRPV2/TRPV1/SLC24A3/TRPM2/CACNG2/TRPM8/CACNA2D3	4.8822E-09	2.315E-07
GO:0015077	Monovalent inorganic cation transmembrane transporter activity	KCNMA1/SCNN1A/SCN8A/SLC25A3/SLC24A4/CACNA1H/ATP2C2/KCNAB3/KCND3/SLC24A3/SNAP25/SLC12A5/TRPM2/SCN3A/SCN11A/SCN5A/SLC39A8/SLC6A3/KCNQ5/KCND2/KCNB2	3.2118E-08	1.4058E-06
GO:0008528	G protein-coupled peptide receptor activity	AVPR1A/MC2R/MC4R/GALR1/HCRTR1/OPRD1/TACR1/RAMP1/CX3CR1/NPSR1/OPRK1/SIGMAR1	5.7785E-07	2.3486E-05
GO:0001653	Peptide receptor activity	AVPR1A/MC2R/MC4R/GALR1/HCRTR1/OPRD1/TACR1/RAMP1/CX3CR1/NPSR1/OPRK1/SIGMAR1	7.6948E-07	2.9189E-05
GO:0008083	Growth factor activity	BDNF/FGF3/FGF6/TGFB1/TGFA/EREG/FGF2/PDGFC/IL12B/BMP6/PTN/NRG1	1.5156E-06	5.0909E-05
GO:0005272	Sodium channel activity	SCNN1A/SCN8A/CACNA1H/TRPM2/SCN3A/SCN11A/SCN5A	1.521E-06	5.0909E-05
GO:0030594	Neurotransmitter receptor activity	DRD2/GRIN2B/HTR2A/GABRB3/GRIN2A/CHRM3/GABRB1/GABBR1/GRM1/CHRM2	1.9697E-06	6.2263E-05
GO:0099528	G protein-coupled neurotransmitter receptor activity	CHRM3/GABBR1/GRM1/CHRM2	5.6317E-06	0.00016865
GO:0005248	Voltage-gated sodium channel activity	SCN8A/CACNA1H/SCN3A/SCN11A/SCN5A	1.0184E-05	0.00028975
GO:0099604	Ligand-gated calcium channel activity	GRIN2B/GRIN2A/TRPV1/TRPM2/TRPM8	1.9282E-05	0.00052245
GO:0098960	Postsynaptic neurotransmitter receptor activity	DRD2/GABRB3/CHRM3/GABRB1/GABBR1/GRM1/CHRM2	2.1887E-05	0.00056607
GO:0048018	Receptor ligand activity	BDNF/FGF3/FGF6/IFNG/TGFB1/IL19/TGFA/POMC/DPP4/EREG/FGF2/PDGFC/IL12B/LTA/BMP6/PTN/NRG1/PENK/TG	2.3641E-05	0.00058485
GO:0030546	Signaling receptor activator activity	BDNF/FGF3/FGF6/IFNG/TGFB1/IL19/TGFA/POMC/DPP4/EREG/FGF2/PDGFC/IL12B/LTA/BMP6/PTN/NRG1/PENK/TG	2.7188E-05	0.00064458
GO:0015081	Sodium ion transmembrane transporter activity	SCNN1A/SCN8A/SLC24A4/CACNA1H/SLC24A3/TRPM2/SCN3A/SCN11A/SCN5A/SLC6A3	3.2126E-05	0.00073119

GO:0015079	Potassium ion transmembrane transporter activity	KCNMA1/SLC24A4/KCNAB3/KCND3/SLC24A3/SNAP25/SLC12A5/KCNQ5/KCND2/KCNB2	4.012E-05	0.00087802
GO:0015103	Inorganic anion transmembrane transporter activity	GABRB3/CLCN6/SLC12A5/GABRB1/SLC39A8/ANKH/SLC6A3/CLIC5/CLIC1	0.00012488	0.00263174
GO:0005249	Voltage-gated potassium channel activity	KCNMA1/KCNAB3/KCND3/SNAP25/KCNQ5/KCND2/KCNB2	0.00015474	0.00314447
GO:0005245	Voltage-gated calcium channel activity	CACNB2/CACNA1C/CACNA1H/CACNG2/CACNA2D3	0.00026283	0.00515696
GO:0035254	Glutamate receptor binding	DRD2/GNAS/CACNG2/SHANK3/CAMK2A	0.00032478	0.00615996
GO:0042923	Neuropeptide binding	MC4R/GALR1/OPRD1/OPRK1	0.00034007	0.00624198
GO:0015108	Chloride transmembrane transporter activity	GABRB3/CLCN6/SLC12A5/GABRB1/SLC6A3/CLIC5/CLIC1	0.00038571	0.00678108
GO:0008066	Glutamate receptor activity	GRIN2B/GRIN2A/GRM7/GRM1	0.00039514	0.00678108
GO:0019955	Cytokine binding	NRP1/TNFRSF1B/IL1R2/IL18R1/CX3CR1/TGFBR2/FGF2/IL12B	0.0004052	0.00678108
GO:0019838	Growth factor binding	NRP1/HTRA1/COL4A1/PCSK6/IL1R2/SCN5A/TGFBR2/PTN	0.00042575	0.00692149
GO:0035255	Ionotropic glutamate receptor binding	DRD2/GNAS/CACNG2/SHANK3	0.0004563	0.00721209
GO:0015276	Ligand-gated ion channel activity	GRIN2B/SCNN1A/GABRB3/GRIN2A/TRPV1/TRPM2/TRPM8/GABRB1	0.00049255	0.00737533
GO:0022834	Ligand-gated channel activity	GRIN2B/SCNN1A/GABRB3/GRIN2A/TRPV1/TRPM2/TRPM8/GABRB1	0.00049255	0.00737533
GO:0001223	Transcription coactivator binding	RORA/SMAD3/VGLL4/AHR	0.00052391	0.00750846
GO:0017046	Peptide hormone binding	AVPR1A/MC4R/GALR1/HCRTR1/RAMP1	0.00052784	0.00750846
GO:0022804	Active transmembrane transporter activity	ABCC2/SLC25A3/ABCC4/SLC24A4/ATP2C2/CLCN6/SLC24A3/SLC12A5/ABCG1/SLC39A8/SLC10A7/ANKH/SLC6A3	0.0006284	0.00872097
GO:0008509	Anion transmembrane transporter activity	ABCC2/SLC24A4/GABRB3/CLCN6/SLC24A3/SLC12A5/GABRB1/SLC39A8/SLC10A7/ANKH/SLC6A3/CLIC5/CLIC1	0.0008845	0.01183867
GO:0035259	Glucocorticoid receptor binding	NCOR2/SMAD3/NRIP1	0.00089466	0.01183867
GO:0005267	Potassium channel activity	KCNMA1/KCNAB3/KCND3/SNAP25/KCNQ5/KCND2/KCNB2	0.00101931	0.01318157
GO:0008081	Phosphoric diester hydrolase activity	MPPED2/PLCB3/CHRM3/PLCB1/ENPP1/PDE10A	0.00110508	0.01380124
GO:0097110	Scaffold protein binding	MAP2K1/CACNA1H/SHANK3/SCN5A/NOS3	0.00111574	0.01380124

GO:0004708	MAP kinase kinase activity	MAP2K1/MAP2K6/MAPK10	0.00155868	0.01887
GO:0042277	Peptide binding	GRIN2B/LRP1/AVPR1A/GRIN2A/MC4R/GALR1/HCRTR1/OPRD1/RAMP1/HLA-DPA1/OPRK1	0.00252415	0.02987047
GO:0070851	Growth factor receptor binding	FGF3/FGF6/TGFA/EREG/FGF2/PDGFC/IL12B	0.00257233	0.02987047
GO:0015293	Symporter activity	SLC25A3/SLC24A4/SLC24A3/SLC12A5/SLC39A8/SLC10A7/SLC6A3	0.00278342	0.02991806
GO:0008188	Neuropeptide receptor activity	MC2R/GALR1/TACR1/NPSR1	0.00279145	0.02991806
GO:0015296	Anion:cation symporter activity	SLC12A5/SLC39A8/SLC6A3	0.00283043	0.02991806
GO:0016917	GABA receptor activity	GABRB3/GABRB1/GABBR1	0.00283043	0.02991806
GO:0005230	Extracellular ligand-gated ion channel activity	GRIN2B/GABRB3/GRIN2A/TRPV1/GABRB1	0.00283932	0.02991806
GO:0099094	Ligand-gated cation channel activity	GRIN2B/SCNN1A/GRIN2A/TRPV1/TRPM2/TRPM8	0.00296496	0.03058686
GO:0005254	Chloride channel activity	GABRB3/CLCN6/GABRB1/CLIC5/CLIC1	0.00301031	0.03058686
GO:0005160	Transforming growth factor beta receptor binding	SMAD3/TGFB1/TGFBR2	0.00364913	0.0364273
GO:0015291	Secondary active transmembrane transporter activity	SLC25A3/SLC24A4/CLCN6/SLC24A3/SLC12A5/SLC39A8/SLC10A7/ANKH/SLC6A3	0.00398303	0.03907485
GO:0005104	Fibroblast growth factor receptor binding	FGF3/FGF6/FGF2	0.0041071	0.03960913
GO:0001221	Transcription cofactor binding	RORA/SMAD3/VGLL4/AHR	0.00440511	0.04109024
GO:0008227	G protein-coupled amine receptor activity	HTR2A/CHRM3/CHRM2/ADRA1A	0.00440511	0.04109024
GO:0004435	Phosphatidylinositol phospholipase C activity	PLCB3/CHRM3/PLCB1	0.00459845	0.04199971
GO:0004879	Nuclear receptor activity	ESR2/ESRRB/RORA/AHR	0.00472404	0.04199971
GO:0098531	Ligand-activated transcription factor activity	ESR2/ESRRB/RORA/AHR	0.00472404	0.04199971
GO:0042562	Hormone binding	AVPR1A/MC4R/GALR1/HCRTR1/RAMP1	0.00489903	0.04226338
GO:0005516	Calmodulin binding	PLCB3/CACNA1C/TRPV1/PLCB1/SCN5A/CAMK2A/KCNQ5/NOS3	0.00490225	0.04226338
GO:0016705	Oxidoreductase activity, acting on paired donors, with incorporation or reduction of molecular oxygen	KDM2A/TPH2/CYP19A1/CYP1A2/HMOX2/FTO/NOS3	0.00551416	0.0468292

GO:0005253	Anion channel activity	GABRB3/CLCN6/GABRB1/CLIC5/CLIC1	0.00568204	0.04687215
GO:0004629	Phospholipase C activity	PLCB3/CHRM3/PLCB1	0.00568397	0.04687215
GO:0005080	Protein kinase C binding	GRK5/AVPR1A/PARD6G/IRS1	0.00577309	0.04692701
