# Supplementary material for: Impaired Molecular Mechanisms Contributing to Chronic Pain in Patients with Hidradenitis Suppurativa: Exploring Potential Biomarkers and Therapeutic Targets
Source: Int J Mol Sci. 2025 Jan 25;26(3):1039. doi: 10.3390/ijms26031039 (PMC11817842; doi:10.3390/ijms26031039)
Supplement: Supplementary file 1 [file ijms-26-01039-s001.zip › Supplementary Table S7.pdf]

Supplementary Table S7. Comprehensive List of Enriched KEGG Pathways

ID	Description	Gene ID	p-value	p.adjust
hsa04020	Calcium signaling pathway	PLCB3/FGF3/GRIN2B/FGF6/CACNA1C/AVPR1A/HTR2A/GRIN2A/CACNA1H/PRKCA/CHRM3/PLCB1/GNAS/TACR1/TGFA/FGF2/PDGFC/CAMK2A/GRM1/CHRM2/NOS3/ADRA1A	8.62638E-10	2.26011E-07
hsa04080	Neuroactive ligand-receptor interaction	DRD2/GRIN2B/AVPR1A/HTR2A/GABRB3/GRIN2A/TRPV1/MC2R/MC4R/GALR1/ADORA3/HCRTR1/OPRD1/CNR2/CHRM3/TACR1/POMC/GRM7/GABRB1/GABBR1/GRM1/NPSR1/CHRM2/ADRA1A/OPRK1/PENK	1.85965E-09	2.43614E-07
hsa04010	MAPK signaling pathway	CACNB2/BDNF/FGF3/FGF6/CACNA1C/TAOK3/MAP2K1/CACNA1H/NF1/PRKCA/MAP2K6/TGFB1/CACNG2/TGFA/CACNA2D3/TGFBR2/EREG/MAPK10/FGF2/PDGFC	4.23953E-07	3.70252E-05
hsa04540	Gap junction	PRKG1/PLCB3/DRD2/HTR2A/MAP2K1/PRKCA/CSNK1D/PLCB1/GNAS/PDGFC/GRM1	7.3313E-07	4.802E-05
hsa04015	Rap1 signaling pathway	PLCB3/FGF3/DRD2/GRIN2B/LPAR5/FGF6/MAP2K1/GRIN2A/PRKCA/MAP2K6/PARD6G/PLCB1/GNAS/FGF2/PDGFC/DOCK4	1.30358E-06	6.73238E-05
hsa04750	Inflammatory mediator regulation of TRP channels	PLCB3/HTR2A/TRPV2/TRPV1/PRKCA/MAP2K6/PLCB1/GNAS/TRPM8/MAPK10/CAMK2A	1.54177E-06	6.73238E-05
hsa04720	Long-term potentiation	PLCB3/GRIN2B/CACNA1C/MAP2K1/GRIN2A/PRKCA/PLCB1/CAMK2A/GRM1	2.89182E-06	0.000108237
hsa04728	Dopaminergic synapse	ARNTL/PLCB3/DRD2/GRIN2B/CACNA1C/GRIN2A/PRKCA/PLCB1/GNAS/MAPK10/SLC6A3/CAMK2A	4.36574E-06	0.000142978
hsa04912	GnRH signaling pathway	PLCB3/CACNA1C/MAP2K1/MMP2/PRKCA/MAP2K6/PLCB1/GNAS/MAPK10/CAMK2A	6.31226E-06	0.000177774
hsa04726	Serotonergic synapse	PLCB3/CACNA1C/TPH2/HTR2A/GABRB3/MAP2K1/PRKCA/PLCB1/GNAS/GABRB1/KCND2	6.78526E-06	0.000177774
hsa04713	Circadian entrainment	PRKG1/PLCB3/GRIN2B/CACNA1C/GRIN2A/CACNA1H/PRKCA/PLCB1/GNAS/CAMK2A	9.23493E-06	0.000219959
hsa04933	AGE-RAGE signaling pathway in diabetic complications	PLCB3/COL4A1/SMAD3/MMP2/PRKCA/TGFB1/PLCB1/TGFBR2/MAPK10/NOS3	1.32598E-05	0.000275274
hsa04024	cAMP signaling pathway	BDNF/DRD2/GRIN2B/CACNA1C/ABCC4/MAP2K1/GRIN2A/MC2R/GNAS/POMC/MAPK10/CAMK2A/GABBR1/PDE10A/CHRM2	1.36586E-05	0.000275274
hsa04072	Phospholipase D signaling pathway	PLCB3/LPAR5/AVPR1A/MAP2K1/TSC2/PRKCA/PLCB1/GNAS/GRM7/PDGFC/GRM1/DGKI	1.52258E-05	0.000284939

hsa04929	GnRH secretion	PLCB3/CACNA1C/ESR2/MAP2K1/CACNA1H/PRKCA/PLCB1/GABBR1	2.02771E-05	0.000303438
hsa05321	Inflammatory bowel disease	STAT6/IFNG/RORA/SMAD3/TGFB1/IL18R1/IL12B/HLA-DPA1	2.02771E-05	0.000303438
hsa04921	Oxytocin signaling pathway	CACNB2/PLCB3/CACNA1C/MAP2K1/PRKCA/PLCB1/GNAS/TRPM2/CACNG2/CACNA2D3/CAMK2A/NOS3	2.12493E-05	0.000303438
hsa04926	Relaxin signaling pathway	PLCB3/COL4A1/MAP2K1/MMP2/PRKCA/TGFB1/PLCB1/GNAS/TGFBR2/MAPK10/NOS3	2.19494E-05	0.000303438
hsa04911	Insulin secretion	KCNMA1/PLCB3/CACNA1C/PRKCA/CHRM3/SNAP25/PLCB1/GNAS/CAMK2A	2.31986E-05	0.000303438
hsa04659	Th17 cell differentiation	STAT6/IFNG/RORA/SMAD3/TGFB1/RUNX1/TGFBR2/MAPK10/HLA-DPA1/AHR	2.39783E-05	0.000303438
hsa05030	Cocaine addiction	BDNF/DRD2/GRIN2B/GRIN2A/RGS9/GNAS/SLC6A3	2.52518E-05	0.000303438
hsa05210	Colorectal cancer	MAP2K1/SMAD3/DCC/TGFB1/CASP9/TGFA/TGFBR2/EREG/MAPK10	2.54795E-05	0.000303438
hsa04270	Vascular smooth muscle contraction	PRKG1/KCNMA1/PLCB3/CACNA1C/AVPR1A/MAP2K1/PRKCA/PLCB1/GNAS/RAMP1/ADRA1A	2.91739E-05	0.000332329
hsa04915	Estrogen signaling pathway	PLCB3/ESR2/MAP2K1/MMP2/PLCB1/GNAS/TGFA/POMC/GABBR1/GRM1/NOS3	4.10229E-05	0.000447834
hsa04724	Glutamatergic synapse	PLCB3/GRIN2B/CACNA1C/GRIN2A/PRKCA/PLCB1/GNAS/SHANK3/GRM7/GRM1	4.46267E-05	0.000467688
hsa04925	Aldosterone synthesis and secretion	PLCB3/CACNA1C/CACNA1H/PRKCA/MC2R/PLCB1/GNAS/POMC/CAMK2A	6.59865E-05	0.000664941
hsa04730	Long-term depression	PRKG1/PLCB3/MAP2K1/PRKCA/PLCB1/GNAS/GRM1	9.6104E-05	0.000911762
hsa05146	Amoebiasis	PLCB3/IFNG/COL4A1/PRKCA/TGFB1/PLCB1/GNAS/IL1R2/IL12B	9.74402E-05	0.000911762
hsa04261	Adrenergic signaling in cardiomyocytes	CACNB2/PLCB3/CACNA1C/PRKCA/PLCB1/GNAS/CACNG2/CACNA2D3/SCN5A/CAMK2A/ADRA1A	0.000104474	0.000943868
hsa04742	Taste transduction	PLCB3/SCNN1A/CACNA1C/CHRM3/PLCB1/SCN3A/GABBR1/GRM1	0.000154607	0.001350239
hsa04927	Cortisol synthesis and secretion	PLCB3/CACNA1C/CACNA1H/MC2R/PLCB1/GNAS/POMC	0.000160621	0.001357505
hsa04022	cGMP-PKG signaling pathway	PRKG1/KCNMA1/PLCB3/CACNA1C/MAP2K1/ADORA3/OPRD1/PLCB1/IRS1/NOS3/ADRA1A	0.000203386	0.001665222
hsa04725	Cholinergic synapse	PLCB3/CACNA1C/MAP2K1/PRKCA/CHRM3/PLCB1/CAMK2A/KCNQ5/CHRM2	0.000227009	0.001802315
hsa05031	Amphetamine addiction	GRIN2B/CACNA1C/GRIN2A/PRKCA/GNAS/SLC6A3/CAMK2A	0.000234314	0.001805596
hsa04658	Th1 and Th2 cell differentiation	MAML2/STAT6/IFNG/NOTCH3/MAPK10/MAML3/IL12B/HLA-DPA1	0.000247483	0.001852585
hsa05017	Spinocerebellar ataxia	PLCB3/GRIN2B/RORA/GRIN2A/PRKCA/KCND3/PLCB1/MAPK10/ATXN1/GRM1	0.000272303	0.001981759

hsa04151	PI3K-Akt signaling pathway	BDNF/FGF3/LPAR5/FGF6/COL4A1/MAP2K1/TSC2/PRKCA/CASP9/TGFA/IRS1/EREG/FGF2/PDGFC/CHRM2/NOS3/TNC	0.000284294	0.00201311
hsa04071	Sphingolipid signaling pathway	PLCB3/SPTLC2/MAP2K1/PRKCA/ADORA3/OPRD1/PLCB1/MAPK10/NOS3	0.000353579	0.002332034
hsa04935	Growth hormone synthesis, secretion and action	PLCB3/CACNA1C/MAP2K1/PRKCA/MAP2K6/PLCB1/GNAS/IRS1/MAPK10	0.000353579	0.002332034
hsa04970	Salivary secretion	PRKG1/KCNMA1/PLCB3/PRKCA/CHRM3/PLCB1/GNAS/ADRA1A	0.000356036	0.002332034
hsa05212	Pancreatic cancer	MAP2K1/SMAD3/TGFB1/CASP9/TGFA/TGFBR2/MAPK10	0.000462861	0.002957794
hsa04934	Cushing syndrome	PLCB3/CACNA1C/MAP2K1/CACNA1H/MC2R/PLCB1/GNAS/POMC/CAMK2A/AHR	0.000490955	0.003029646
hsa05143	African trypanosomiasis	PLCB3/IFNG/PRKCA/PLCB1/IL12B	0.000497232	0.003029646
hsa05417	Lipid and atherosclerosis	PLCB3/MMP3/PRKCA/MAP2K6/CASP9/PLCB1/ABCG1/MAPK10/CAMK2A/IL12B/SOD2/NOS3	0.000533753	0.003112335
hsa05142	Chagas disease	PLCB3/IFNG/TGFB1/PLCB1/GNAS/TGFBR2/MAPK10/IL12B	0.000534561	0.003112335
hsa01521	EGFR tyrosine kinase inhibitor resistance	MAP2K1/NF1/PRKCA/TGFA/FGF2/PDGFC/NRG1	0.00058439	0.003328481
hsa04068	FoxO signaling pathway	RAG1/MAP2K1/SMAD3/TGFB1/IRS1/TGFBR2/MAPK10/GRM1/SOD2	0.000666324	0.003714401
hsa04330	Notch signaling pathway	MAML2/NCOR2/NOTCH3/MAML3/NOTCH4/ATXN1	0.000850634	0.004643042
hsa04012	ErbB signaling pathway	MAP2K1/PRKCA/TGFA/EREG/MAPK10/CAMK2A/NRG1	0.000903031	0.004828449
hsa04940	Type I diabetes mellitus	IFNG/IL12B/HLA-DPA1/LTA/ICA1	0.001006296	0.005272989
hsa05022	Pathways of neurodegeneration - multiple diseases	BDNF/CAPN1/PLCB3/GRIN2B/CACNA1C/MAP2K1/GRIN2A/PRKCA/MAP2K6/CASP9/TNFRSF1B/CHRM3/PLCB1/MAPK10/SLC6A3/CAMK2A/ATXN1/GRM1/SIGMAR1	0.00111813	0.005744119
hsa05415	Diabetic cardiomyopathy	PLCB3/SMAD3/MMP2/PRKCA/TGFB1/PLCB1/IRS1/TGFBR2/MAPK10/CAMK2A/NOS3	0.001209092	0.006091963
hsa04014	Ras signaling pathway	BDNF/FGF3/GRIN2B/FGF6/MAP2K1/GRIN2A/NF1/PRKCA/TGFA/MAPK10/FGF2/PDGFC	0.001250563	0.006121614
hsa05032	Morphine addiction	GRK5/GABRB3/PRKCA/GNAS/GABRB1/GABBR1/PDE10A	0.001261707	0.006121614
hsa04723	Retrograde endocannabinoid signaling	PLCB3/CACNA1C/GABRB3/PRKCA/FAAH/PLCB1/GABRB1/MAPK10/GRM1	0.001494096	0.007117328
hsa04919	Thyroid hormone signaling pathway	PLCB3/MAP2K1/TSC2/PRKCA/NOTCH3/CASP9/PLCB1/NOTCH4	0.001621572	0.007586641
hsa01522	Endocrine resistance	ESR2/MAP2K1/MMP2/NOTCH3/GNAS/MAPK10/NOTCH4	0.002057791	0.009295538
hsa05231	Choline metabolism in cancer	MAP2K1/TSC2/PRKCA/SLC44A2/MAPK10/PDGFC/DGKI	0.002057791	0.009295538
hsa04390	Hippo signaling pathway	TEAD1/DLG2/SMAD3/CSNK1D/PARD6G/TGFB1/CTNNA2/TGFBR2/BMP6	0.002143872	0.009520245

hsa04916	Melanogenesis	PLCB3/MAP2K1/PRKCA/PLCB1/GNAS/POMC/CAMK2A	0.00230715	0.010074557
hsa04971	Gastric acid secretion	PLCB3/PRKCA/CHRM3/PLCB1/GNAS/CAMK2A	0.002458539	0.010559627
hsa05161	Hepatitis B	STAT6/MAP2K1/SMAD3/PRKCA/MAP2K6/TGFB1/CASP9/TGFBR2/MAPK10	0.002765444	0.011686231
hsa05145	Toxoplasmosis	IFNG/MAP2K6/TGFB1/CASP9/MAPK10/IL12B/HLA-DPA1	0.003921784	0.016309641
hsa04727	GABAergic synapse	CACNA1C/GABRB3/PRKCA/SLC12A5/GABRB1/GABBR1	0.005394636	0.02208429
hsa05207	Chemical carcinogenesis - receptor activation	FGF3/FGF6/CACNA1C/ESR2/MAP2K1/CYP1A2/PRKCA/GNAS/FGF2/AHR	0.005593147	0.022544685
hsa04668	TNF signaling pathway	MMP3/MAP2K1/MAP2K6/TNFRSF1B/IL18R1/MAPK10/LTA	0.005738989	0.022655008
hsa05226	Gastric cancer	FGF3/FGF6/MAP2K1/SMAD3/TGFB1/CTNNA2/TGFBR2/FGF2	0.005793456	0.022655008
hsa05033	Nicotine addiction	GRIN2B/GABRB3/GRIN2A/GABRB1	0.006210677	0.023929373
hsa04924	Renin secretion	KCNMA1/PLCB3/CACNA1C/PLCB1/GNAS	0.008069313	0.030639999
hsa05215	Prostate cancer	MMP3/MAP2K1/CASP9/TGFA/IL1R2/PDGFC	0.008560957	0.03204244
hsa05218	Melanoma	FGF3/FGF6/MAP2K1/FGF2/PDGFC	0.010182793	0.037575942
hsa05010	Alzheimer disease	CAPN1/PLCB3/GRIN2B/CACNA1C/LRP1/SLC39A9/MAP2K1/GRIN2A/CASP9/CHRM3/PLCB1/IRS1/MAPK10/SLC39A8	0.011235614	0.04080729
hsa04918	Thyroid hormone synthesis	PLCB3/PRKCA/PLCB1/GNAS/TG	0.01136997	0.04080729
hsa05414	Dilated cardiomyopathy	CACNB2/CACNA1C/TGFB1/GNAS/CACNG2/CACNA2D3	0.011812257	0.041821774
hsa04972	Pancreatic secretion	KCNMA1/PLCB3/PRKCA/CHRM3/PLCB1/GNAS	0.012339021	0.043104315
hsa05220	Chronic myeloid leukemia	MAP2K1/SMAD3/TGFB1/RUNX1/TGFBR2	0.012648254	0.043603193
